# Multiscale dynamic immunomodulation by a nanoemulsified Trojan-TLR7/8 adjuvant for robust protection against heterologous pandemic and endemic viruses

**DOI:** 10.1038/s41423-025-01306-6

**Published:** 2025-06-25

**Authors:** Yeon Jeong Yoo, Suhyeon Kim, Asha Wickramasinghe, Jaemoo Kim, JuA Song, Young-Il Kim, Juryeon Gil, Young-Woock Noh, Min-Ho Lee, Sang-Seok Oh, Myeong-Mi Lee, Yebin Seong, Jong-Soo Lee, Young Ki Choi, Yong Taik Lim

**Affiliations:** 1 SKKU Advanced Institute of Nanotechnology (SAINT), Department of Nano Science and Technology, Department of Nano Engineering, School of Chemical Engineering, and Biomedical Institute for Convergence at SKKU, Sungkyunkwan University, Suwon, Republic of Korea; 2 College of Veterinary Medicine, Chungnam National University, Daejeon, Republic of Korea; 3 Center for Study of Emerging and Re-emerging Viruses, Korea Virus Research Institute, Institute for Basic Science (IBS), Daejeon, Republic of Korea; 4 College of Medicine and Medical Research Institute, Chungbuk National University, Cheongju, Republic of Korea; 5 New Drug Development Center, Osong Medical Innovation Foundation, Cheongju, Republic of Korea; 6 College of Pharmacy, Chungbuk National University, Cheongju, Republic of Korea

**Keywords:** Dynamic immunomodulation, Vaccine adjuvant, Nanoemulsion, TLR7/8 agonist, Heterologous viruses, Adjuvants, Immunotherapy, Infectious diseases

## Abstract

The demand for safe vaccines that ensure long-term and broad protection against multiple viral variants has dramatically increased after the emergence of catastrophic infectious diseases such as COVID-19. To ensure long-term and broad protection against heterologous virus variants, antigen-specific polyfunctional T cells should be orchestrated with the activation of follicular helper T (T_FH_) cells and germinal center (GC) B cells. Herein, we suggest a novel engineered nanoadjuvant (SE(Trojan-TLR7/8a)) that enhances the migration of nonexhausted antigen-presenting cells (APCs) into lymph nodes and elicits the activation of T_FH_ cells, the generation of GC B cells, and polyfunctional T cells via multiscale dynamic immunomodulation through squalene nanoemulsion (SE)-mediated macroscopic control of vaccine delivery and Trojan-TLR7/8a-enabled dynamic and sustained activation of APCs at the cellular level. SE(Trojan-TLR7/8a) can be lyophilized, reduce systemic toxicity, and outperform current commercial vaccine adjuvants (Alum or AS03) and mRNA vaccines. SE(Trojan-TLR7/8a) ensures cross-protection against diverse influenza and SARS-CoV-2 variants, providing 100% protection while maintaining a healthy state. SE(Trojan-TLR7/8a) also sustains a potent T-cell response in an aged ferret model of SFTSV infection. SE(Trojan-TLR7/8a) suggested herein provides a novel vaccine design principle for dynamic modulation at the multiscale level and demonstrates long-term and broad protective immunity against emerging pandemic and endemic infectious viruses.

## Introduction

Persistent outbreaks of emerging infectious diseases such as the COVID-19 pandemic and recent concerns about the spread of H5N1 avian influenza from cattle to humans have increased the importance of innovative vaccines that can not only adapt to rapidly mutating viruses but also induce a durable immune response [1, 2]. While mRNA vaccines have marked a paradigm shift in our fight against severe acute respiratory syndrome coronavirus 2 (SARS-CoV-2), garnering FDA approval as the frontrunners, there are still challenges to be addressed regarding their long-term and broad protection against various SARS-CoV-2 variants and storage conditions [3, 4]. Subunit vaccines are among the safest types of vaccines and have been proven to be highly effective against a variety of infectious diseases because premade proteins are used as antigens rather than genetic materials, which should be translated inside the body [5]. However, owing to the inherently low immunogenicity of subunit antigens, the use of immunostimulatory adjuvants to increase the magnitude and persistence of the immune response is necessary [6]. However, current subunit vaccines using clinically approved adjuvants such as squalene-based oils and aluminum hydroxide (Alum) still have limited protective efficacy because of the multiple variants of the influenza virus and SARS-CoV-2 [7, 8].

**Fig. 1:**
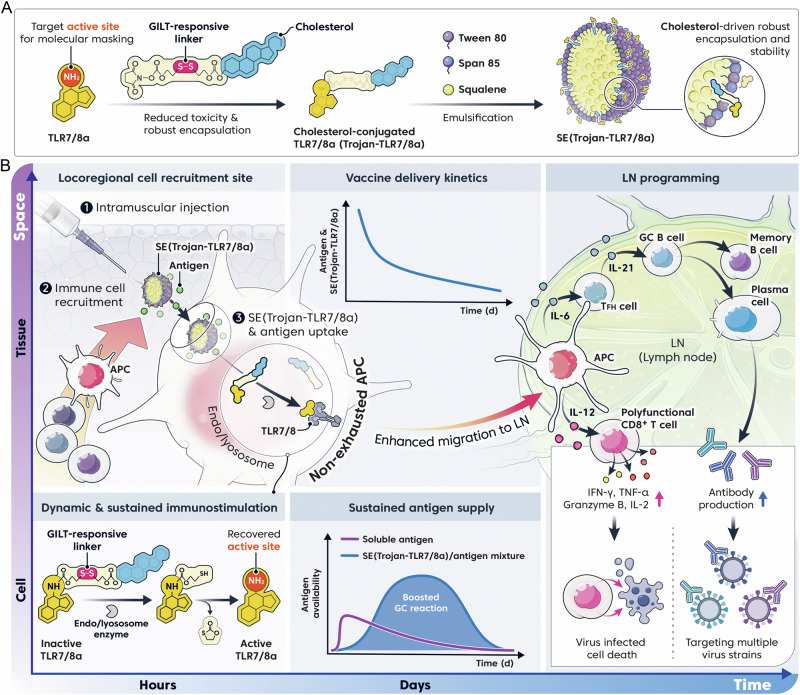
Schematic of multiscale dynamic immunomodulation by a lyophilizable squalene-based nanoemulsion loaded with a Trojan-TLR7/8 agonist (SE(Trojan-TLR7/8a)) to enhance broad and sustained protection. **A** Synthesis process of SE(Trojan-TLR7/8a). **B** Mechanism of action in promoting sustained cellular and humoral responses

Vaccine-induced immunity highly depends on the spatiotemporal profile of antigens and adjuvants [9]. For example, compared with conventional bolus immunization strategies, slow delivery immunization via an osmotic pump enhances neutralizing antibody and germinal center (GC) reactions in response to HIV Env protein immunization [10]. Therefore, we hypothesize that the programmed immune modulation strategy at the optimal time and location can provide efficient protective immunity against heterologous variants of viruses. Furthermore, recent data suggest that antigen-specific polyfunctional T cells should be orchestrated with the activation of follicular helper T (T_FH_) cells and GC B cells to ensure long-term and broad protection against heterologous virus variants [11–13]. In this study, we suggest a multiscale dynamic immunomodulation strategy that can enhance not only the breadth, magnitude, and persistence of antibody responses but also potent CD8^+^ T-cell responses through the combination of controlled vaccine delivery kinetics at the macroscopic level and timely activation of antigen-presenting cells (APCs) at the cellular level. Among the various Toll-like receptor (TLR) agonists, we have selected TLR7/8 agonists (TLR7/8a) because of their outstanding ability to enhance cellular immunity and activate T_FH_ cells, which play crucial roles in stimulating GC B cells that can generate broad neutralizing antibodies [14–16]. However, their rapid dissemination from the injection site into the bloodstream can cause systemic toxicity [17]. To address this issue, we designed and synthesized a timely activated TLR7/8a, which has “Trojan horse-like” characteristics (Trojan-TLR7/8a) in which the putative active site (C4 amine) is transiently obscured by a cholesterol block linked to an enzymatically cleavable moiety [18]. Considering the pivotal role of cholesterol in enhancing nanoparticle stability, the cholesterol moiety in Trojan-TLR7/8a enables high and stable incorporation into the squalene nanoemulsion (SE(Trojan-TLR7/8a)), ensuring robust loading and improved stability (Fig. 1A). At the cellular level, Trojan-TLR7/8a can be reactivated by cleavage of the masking moiety by gamma-interferon-inducible lysosomal thiol reductase (GILT) after uptake into APCs, minimizing systemic toxicity, which is a major hurdle for the clinical translation of the small molecule TLR7/8a (Fig. 1B). Multiscale dynamic immunomodulation by SE(Trojan-TLR7/8a) can control the kinetics of vaccine delivery at the macroscopic level via clinically approved SE and timely activation of APCs at the cellular level via Trojan-TLR7/8a (Fig. 1B). SE(Trojan-TLR7/8a) enhances the locoregional recruitment of innate immune cells and the cellular uptake of vaccines, inducing nonexhausted APCs via the timely activation of Trojan-TLR7/8a, which secretes IL-12(p70) in a sustained manner, thereby enhancing the migratory capacity of APCs. This promotes the efficient delivery of SE(Trojan-TLR7/8a) and antigens to lymph nodes (LNs), thereby enhancing both humoral and cellular responses, including increased activity of T_FH_ cells and GC B cells and increased antigen-specific polyfunctional T-cell responses. SE(Trojan-TLR7/8a) demonstrated cross-protection in BALB/c mice against divergent influenza (H1N1, H5N2, H7N3, H9N2, and H3N2) strains via sM2HA2 as the antigen and SARS-CoV-2 (Alpha, Beta, Wuhan, Delta, and Omicron BA.2) variants via spike-stabilized trimers from the BA.2 variant as the antigen. This strategy achieved 100% protection and complete recovery of body weight postinfection by increasing the production of neutralizing antibodies and polyfunctional CD8^+^ T cells. Additionally, in a ferret model of severe fever with thrombocytopenia syndrome virus (SFTSV) infection, a strong T-cell response was noted even at 30 weeks postimmunization.

## Results

### Design and characterization of SE(Trojan-TLR7/8a)

**Fig. 2:**
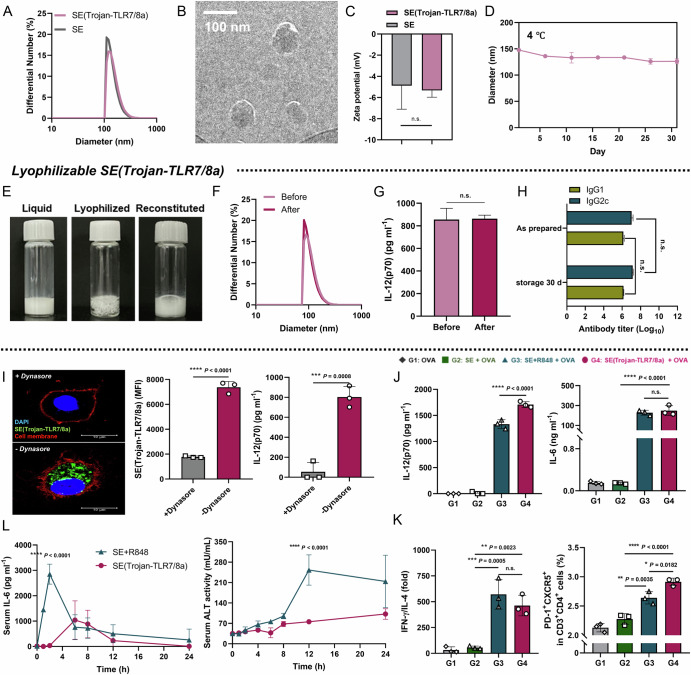
Fabrication and characteristics of SE(Trojan-TLR7/8a). Hydrodynamic size distribution (**A**) and representative transmission electron microscopy image (**B**) of SE(Trojan-TLR7/8a), showing a uniform size and morphology (scale bar, 100 nm). **C** Zeta potentials of SE and SE(Trojan-TLR7/8a) were measured via dynamic light scattering. **D** Average size of SE(Trojan-TLR7/8a) stored at 4 °C for over one month, demonstrating storage stability (*n* = 2). **E** Morphology of SE(Trojan-TLR7/8a) as a liquid (left), lyophilized (middle), and reconstituted (right) form. **F** Hydrodynamic size distribution of SE(Trojan-TLR7/8a) before and after lyophilization. **G** Comparison of IL-12(p70) secretion by SE(Trojan-TLR7/8a) before and after lyophilization (*n* = 3). **H** Comparison of antibody titers in serum after intramuscular immunization with SE(Trojan-TLR7/8a) dispersed immediately after freeze-drying and after 30 days of storage (*n* = 3). **I** Representative confocal laser scanning microscopy (CLSM) image and the mean fluorescence intensity of SE(Trojan-TLR7/8a) and IL-12(p70) secretion in the absence or presence of dynasore (an endocytosis inhibitor, 40 μM) for 24 h (*n* = 3). Scale bar, 10 μm. **J**, **K** Bone marrow-derived dendritic cells (BMDCs) were treated with OVA or SE (the same volume used as SE(Trojan-TLR7/8a), SE + R848 (3.18 μM) or SE(Trojan-TLR7/8a) (3.18 μM) mixed with OVA (10 μg ml^-1^). **J** IL-12(p70) and IL-6 concentrations were measured via ELISA (*n* = 3). **K** Naïve CD4^+^ T cells were cocultured with BMDCs treated for 12 h for 3 days. The ratio of the concentrations of IFN-γ/IL-4 secreted from the coculture supernatants was measured via ELISA, and the percentage of differentiated follicular helper T cells (T_FH_ cells, PD-1^+^ CXCR5^+^ in CD3^+^ CD4^+^) was measured via flow cytometry (*n* = 3). **L** C57BL/6 mice were immunized intramuscularly with SE + R848 (25 μg, 79.5 nmol) or SE(Trojan-TLR7/8a) (72.1 μg, 79.5 nmol), each mixed with OVA (20 μg). Blood was collected at 1, 2, 4, 6, 8, 12, and 24 h following immunization. The concentrations of IL-6 (*n* = 3 mice per group) and ALT (*n* = 2 mice per group) in the serum were quantified via ELISA. The data are presented as the mean ± standard deviation (s.d.). In **J**, **K**, analysis was performed via one-way ANOVA with Tukey’s multiple comparison test; in **H**, **I**, analysis was performed via two-way ANOVA with Tukey’s multiple comparison test; and in **C**, **G**, **I**, analysis was performed via a two-tailed unpaired t test. *P* values are indicated (n.s. not significant; *P < 0.05, **P < 0.01, ***P < 0.001, ****P < 0.0001)

SE(Trojan-TLR7/8a) was fabricated by emulsifying an aqueous phase with Tween 80 and an oil phase comprising a mixture of Trojan-TLR7/8a, squalene, and Span-85 (Fig. 1, Supplementary Fig. S1). Analysis of the physical properties of SE(Trojan-TLR7/8a), including size, shape, and zeta potential, via dynamic light scattering and transmission electron microscopy revealed that the size of SE(Trojan-TLR7/8a) remained stable at approximately 100 nm even after long-term storage (Fig. 2A–D, Supplementary Fig. S2, Supplementary Table S1). Additionally, SE(Trojan-TLR7/8a) can be freeze-dried, providing advantages in terms of storing and distributing heat-sensitive products where cold chain management is challenging (Fig. 2E) [19]. SE(Trojan-TLR7/8a) preserved its size and immunostimulatory function when redispersed immediately or after 30 days of storage at −20 °C following lyophilization (Fig. 2F–H, Supplementary Table S2). To investigate the intracellular mechanism of action of SE(Trojan-TLR7/8a), bone marrow-derived dendritic cells (BMDCs) were pretreated with the endocytosis inhibitor dynasore®. After treatment, SE(Trojan-TLR7/8a) was not detected in the intracellular compartments, and IL-12(p70) secretion was reduced, indicating that SE(Trojan-TLR7/8a) could be delivered into endosomes via endocytosis (Fig. 2I).

Moreover, significant BMDC activation was observed only in the groups treated with SE(Trojan-TLR7/8a) or a mixture of SE and R848 (SE + R848), both of which contained TLR7/8a (Supplementary Fig. S3). Notably, there was a significant increase in the levels of IL-12(p70), which is crucial for Th1 differentiation and initiates an adaptive immune response, and IL-6, which is essential for T_FH_ cell (CXCR5^+^ PD-1^+^ in CD3^+^CD4^+^) differentiation that supports GC B-cell functions, including class-switch recombination and somatic hypermutation (Fig. 2J) [20–22]. Consequently, naïve CD4^+^ T cells cocultured with DCs treated with SE(Trojan-TLR7/8a) biased toward Th1 cells through increased secretion of IFN-γ/IL-4 and increased differentiation into T_FH_ cells (Fig. 2K). Next, we assessed systemic toxicity by measuring the serum levels of IL-6, TNF-α, ALT, and AST. Compared with SE + R848, SE(Trojan-TLR7/8a) administration resulted in reduced systemic toxicity, as indicated by the lower serum levels of these markers (Supplementary Fig. S4). This reduction in toxicity is due to the cholesterol-blocking structure of Trojan-TLR7/8a, which maintains a stable particle form by preventing release and temporarily masks the active site, enabling selective activation within specific cells (Supplementary Figs. S1 and S2f). We further evaluated long-term drug toxicity via hematological and clinical biochemical analyses and detected no observable changes due to the administration of SE(Trojan-TLR7/8a) (Supplementary Tables S3–5).

### Multiscale dynamic immunomodulation by SE(Trojan-TLR7/8a)

#### Macroscopic vaccine kinetics by SE

**Fig. 3:**
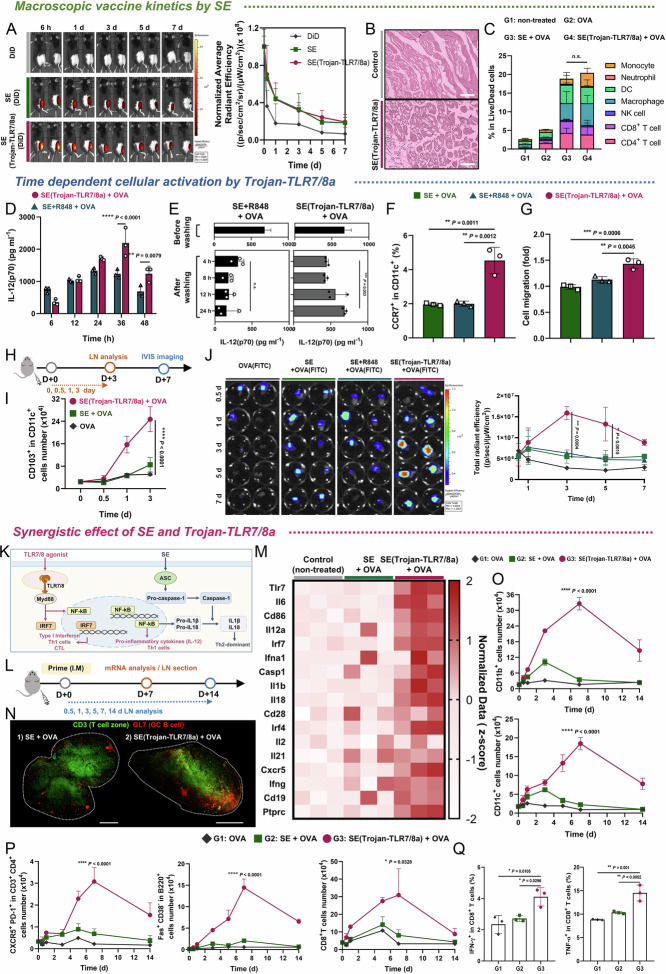
Multiscale dynamic immunomodulation by SE(Trojan-TLR7/8a). **A** C57BL/6 mice were immunized intramuscularly with soluble DiD, SE(DiD), or SE(Trojan-TLR7/8a)(DiD) mixed with OVA on day 0. Representative whole-body fluorescence in vivo imaging system (IVIS) images and normalized average radiant efficiency at the injection site over time (*n* = 2 mice per group). **B** Representative images of H&E-stained muscle tissue showing cell recruitment by SE(Trojan-TLR7/8a) at 24 h. Scale bar: 200 μm. **C** Proportions of monocytes (Ly6G^−^ Ly6C^+^), neutrophils (Ly6G^+^ Ly6C^−^), CD11c^+^, CD11b^+^, NK cells (CD3^−^ NK1.1^+^), CD8^+^ T cells (CD3^+^ CD8^+^), and CD4^+^ T cells (CD3^+^ CD4^+^) recruited into the muscle at 24 h (*n* = 3 mice per group). **D**, **E** Bone marrow-derived dendritic cells (BMDCs) were treated with OVA, SE (the same volume used as SE(Trojan-TLR7/8a)), SE + R848 (3.18 μM) or SE(Trojan-TLR7/8a) (3.18 μM) mixed with OVA (10 μg ml^-1^). **D** Supernatants were harvested at 6, 12, 24, 36, and 48 h, and the kinetics of IL-12(p70) secretion were measured via ELISA (*n* = 3). **E** Secretion of IL-12(p70) by BMDCs was measured after treatment with SE + R848 or SE(Trojan-TLR7/8a) mixed with OVA for 12 h (before washing) and then changed to fresh medium (after washing) (*n* = 3). Expression of CCR7 in BMDCs treated with the indicated samples for 12 h (**F**) and assessment of the cell migration capacity compared with that of the control group via a Transwell assay (**G**) (*n* = 3). **H**, **L** C57BL/6 mice were immunized intramuscularly with OVA (FITC) or OVA alone or in combination with SE (the same volume used as SE(Trojan-TLR7/8a)), SE + R848 (79.5 nmol), or SE(Trojan-TLR7/8a) (79.5 nmol) (*n* = 3 mice per group). **I** The total number of migratory DCs (CD103^+^) in the inguinal LNs (iLNs) on days 0.5, 1, and 3 (*n* = 3 mice per group). **J** OVA(FITC) fluorescence images and total radiant efficiency of excised iLNs at 0.5, 1, 3, 5, and 7 days postadministration were obtained via IVIS (*n* = 2). **K** Synergistic intracellular signaling of TLR7/8a and SE induced by SE(Trojan-TLR7/8a). **M** The expression levels of 17 genes related to humoral and cellular immunity in the iLNs at 7 days postadministration (*n* = 3 mice per group). **N** Immunofluorescence images of sectioned iLNs on day 7 showing the distribution of T cells (CD3, green) and germinal center (GC) B cells (GL7, red) via CLSM observation. Scale bar: 500 μm. Total numbers of CD11b^+^, CD11c^+^ (**O**), T_FH_, GC B (Fas^+^ CD38^−^ in B220^+^), and CD8^+^ T cells (**P**) in the iLNs measured on days 0.5, 1, 3, 5, 7 and 14 (*n* = 3 mice per group). **Q** The total number of antigen-specific CD8^+^ T cells secreting IFN-γ or TNF-α was evaluated on day 7 (*n* = 3 mice per group). The data are presented as the means ± s.d. In **E**–**G**, **Q**, analysis was performed via one-way ANOVA with Tukey’s multiple comparison test; in **C**, **D**, **I**, **J**, **O**, **P**, analysis was performed via two-way ANOVA with Tukey’s multiple comparison test; *P* values are indicated (n.s. not significant; *P < 0.05, **P < 0.01, ***P < 0.001, ****P < 0.0001)

Upon intramuscular administration, SE is known to elicit danger-associated molecular pattern signals, attracting immune cells from the bloodstream to the injection site [23]. In vivo trafficking demonstrated that DiD-loaded SEs and SE(Trojan-TLR7/8a) presented sustained fluorescence intensity at the injection site compared with free DiD, with the signal lasting up to 7 days postimmunization (Fig. 3A). To confirm whether SE(Trojan-TLR7/8a) facilitates immune cell recruitment to muscle, muscle tissue was analyzed one day after intramuscular injection. Hematoxylin and eosin (H&E)-stained muscle tissue images demonstrated that SE(Trojan-TLR7/8a) significantly increased cell recruitment (Fig. 3B). Further cellular analysis revealed that the muscles from the groups containing SE had increased proportions of various immune cells, monocytes (Ly6C^+^ Ly6G^−^), neutrophils (Ly6C^−^ Ly6G^+^), CD11c^+^, CD11b^+^, NK cells (CD3^−^ NK1.1^+^), CD8^+^ T cells (CD3^+^ CD8^+^), and CD4^+^ T cells (CD3^+^ CD4^+^) (Fig. 3C, Supplementary Figs. S5 and 6).

#### Time-dependent cellular activation by Trojan-TLR7/8a

We hypothesized that once immune cells congregate in the muscles, they subsequently migrate to the LNs after the uptake of antigen and SE(Trojan-TLR7/8a) at the injection site. First, we aimed to evaluate the in vitro effects of SE(Trojan-TLR7/8a) on immune cells and determine its specific actions. The timely activation of SE(Trojan-TLR7/8a) resulted in prolonged and sustained activation of BMDCs, facilitated by the GILT-responsive linker designed for intracellular cleavage, as indicated by the continuous secretion of IL-12(p70) (Fig. 3D, Supplementary Fig. S3a). After 12 h of incubation followed by media replacement, SE + R848-treated DCs ceased IL-12(p70) secretion within 4 h (exhausted DCs), whereas SE(Trojan-TLR7/8a)-treated DCs continued to secrete cytokines for 24 h (nonexhausted DCs) (Fig. 3E). Moreover, SE(Trojan-TLR7/8a) increased CCR7 expression, and CCR7 signaling plays a key role in the migration of DCs from the injection site to the LNs (Fig. 3F). Transwell assays demonstrated that SE(Trojan-TLR7/8a)-treated DCs (nonexhausted DCs) exhibited greater migration toward chemoattractants (CCL21 and CCL19) than SE- or SE + R848-treated DCs did (Fig. 3G, Supplementary Fig. S7). On the basis of these in vitro experimental results, we hypothesized that SE(Trojan-TLR7/8a) induces semimature DCs that continuously secrete cytokines, thereby increasing DC migration and antigen delivery to LNs. We found that the total number of migratory DCs (CD103^+^) and resident DCs (CD8α^+^) increased in the SE(Trojan-TLR7/8a) group for up to 3 days (Fig. 3H, I; Supplementary Figs. S8, 9). To study in vivo antigen trafficking, each sample was mixed with FITC-labeled OVA (OVA(FITC)) and administered via intramuscular injection. Compared with that in the SE group, the fluorescence intensity of OVA(FITC) in the inguinal LNs (iLNs) in the SE(Trojan-TLR7/8a)-injected group was greater for up to 7 days (Fig. 3J, Supplementary Fig. S10).

#### Synergistic effects of SE and Trojan-TLR7/8a

Upon administration of SE(Trojan-TLR7/8a), increased DC migration from the muscles to the LNs was observed, indicating that antigen and nanoemulsion delivery was enhanced, thereby resulting in increased immune responses in the LNs. On the basis of these results, we hypothesized that SE(Trojan-TLR7/8a), which involves both the NLRP3-independent ASC activation pathway and the TLR-dependent MyD88 activation pathway, might induce both humoral and cellular immune responses (Fig. 3K) [24]. Compared with SE, SE(Trojan-TLR7/8a) upregulated the expression of cytokines and surface markers that are indicative of DC activation (Il6, CD86, and Il12a), cellular responses (Irf7, Ifna1, and Ifng) and humoral responses (IL18 and IL1β) (Fig. 3L, M) [24, 25]. To determine whether these increases enhance GC formation, which is crucial for the humoral response, we conducted immunofluorescence staining of mouse iLNs 7 days after a single immunization. We confirmed that GC B cells, identified as GL7^+^ cell clusters, represented bona fide GC structures on the basis of their location in the B-cell zone, which was distinct from that of the T-cell zone according to CD3 staining. SE(Trojan-TLR7/8a) markedly increased the number of GC B cells (Fig. 3L, N, Supplementary Fig. S11) [14, 26]. Next, to examine the enhanced activation of immune cells induced by SE(Trojan-TLR7/8a), we analyzed the dynamics of various immune cells associated with both humoral and cellular responses. In the kinetics of CD11b^+^ cells and CD11c^+^ cells, which are innate immune cells, SE(Trojan-TLR7/8a) peaked on day 7, whereas in the other groups, it peaked on day 3 and subsequently declined (Fig. 3O). Compared with those in the other groups, the number of T_FH_ cells, which are critical modulators of GC responses, increased approximately 7-fold on day 7 in the SE(Trojan-TLR7/8a) group. Moreover, the kinetics of GC B (CD38^−^ Fas^+^ in B220^+^) and CD8^+^ T cells also exhibited similar trends, peaking on day 7 before declining (Fig. 3P). We also analyzed the kinetics of neutrophils (Ly6C^−^ Ly6G^+^ in CD11b^+^ CD11c^−^), monocytes (Ly6C^+^ Ly6G^−^ in CD11b^+^ CD11c^−^), cDC2s (XCR1^−^ CD11b^+^ in MHCII^+^ CD11c^+^), and cDC1s (XCR1^+^ CD11b^−^ in MHCII^+^ CD11c^+^) and observed a greater magnitude in the SE(Trojan-TLR7/8a) group (Supplementary Figs. S12–14). Furthermore, the responses of antigen-specific CD8^+^ T cells that secrete IFN-γ, TNF-α and polyfunctional (IFN-γ^+^ TNF-α^+^) CD8^+^ T cells, which are vital for virus clearance, were significantly augmented (Fig. 3Q, Supplementary Figs. S15, 16) [27].

### Increased local and systemic immunity after prime-boost immunization

**Fig. 4:**
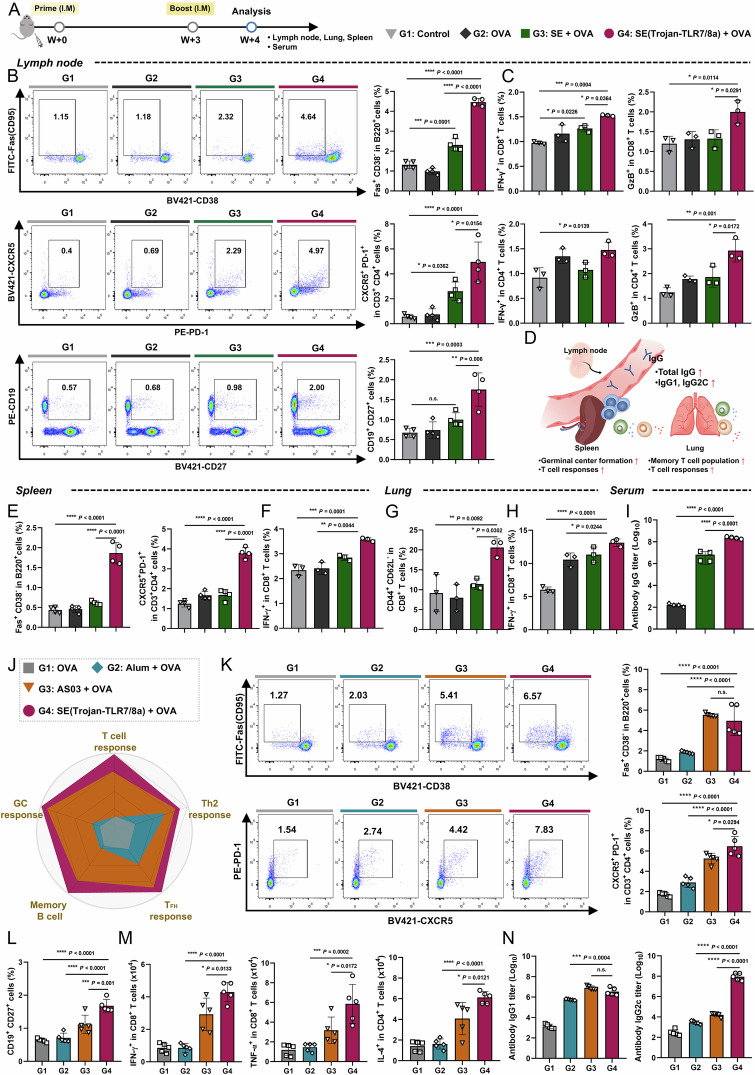
Compared with conventional vaccines, SE(Trojan-TLR7/8a) elicits a high-magnitude immune response. **A**–**I** C57BL/6 mice were immunized intramuscularly with OVA (20 μg) alone or in combination with SE (with the same volume used for SE(Trojan-TLR7/8a)) or SE(Trojan-TLR7/8a) (79.5 nmol) twice at 3-week intervals, after which the iLNs, spleens, and lungs were analyzed according to the same schedule (3‒4 mice per group). **B** Representative flow cytometry plots and analysis of the percentages of GC B cells, T_FH_ cells, and memory B cells in the iLNs (*n* = 4). **C** Percentages of antigen-specific CD8^+^ T cells secreting IFN-γ or granzyme B (GzB) and CD4^+^ T cells secreting IFN-γ or GzB (*n* = 3). **D** Schematic of the systemic responses of the spleen, lung, and blood to SE(Trojan-TLR7/8a). Percentages of GC B cells and T_FH_ cells (**E**) (*n* = 4) and antigen-specific CD8^+^ T cells secreting IFN-γ (**F**) in splenocytes (*n* = 3). Percentages of effector memory (CD44^+^ CD62L^−^) CD8^+^ T cells (**G**) and antigen-specific CD8^+^ T cells secreting IFN-γ (**H**) in the lungs (*n* = 3). **I** Serum samples were collected at 4 weeks, and the titer of OVA-specific IgG was assessed via ELISA (*n* = 4). **J**–**N** C57BL/6 mice were intramuscularly immunized with OVA (20 μg) alone or in combination with alum (200 μg), AS03 (with the same volume used for SE(Trojan-TLR7/8a)), or SE(Trojan-TLR7/8a) (79.5 nmol) twice at 3-week intervals, and then the iLNs and serum were analyzed according to schedule (*n* = 5 mice per group). **J** A diagram comparing the effectiveness of SE(Trojan-TLR7/8a) with that of other squalene-based nanoemulsions (SE and AS03) and alum. **K**, **L** Representative flow cytometry plots and percentages of GC B and T_FH_ cells (**K**), as well as the percentage of memory B cells (**L**) in the iLNs. **M** Total numbers of antigen-specific CD8^+^ T cells secreting IFN-γ or TNF-α and CD4^+^ T cells secreting IL-4 in the iLNs. **N** Serum samples were collected at 4 weeks, and the titer of OVA-specific IgG1 or IgG2c was assessed via ELISA. The data are presented as the means ± s.d. In **B**, **C**, **E**–**I**, **K**–**N**, analysis was performed via one-way ANOVA with Tukey’s multiple comparison test; *P* values are indicated (n.s. not significant; *P < 0.05, **P < 0.01, ***P < 0.001, ****P < 0.0001)

To investigate whether booster immunization could potentiate immune responses, we administered intramuscular immunization twice at 3-week intervals, with analyses performed one week after booster immunization (Fig. 4A, Supplementary Figs. S17 and 18). SE(Trojan-TLR7/8a) significantly increased the percentages of GC B cells, T_FH_ cells, and memory B cells in the iLNs (Fig. 4B). In addition, compared with other treatments, SE(Trojan-TLR7/8a) immunization induced a high magnitude of immune cells involved in humoral immunity, including B cells (CD19^+^ B220^+^), follicular dendritic cells (FDCs) (CD21/35^+^), and plasma cells (B220^−^ CD138^+^) (Supplementary Fig. S19). Similarly, the percentage of antigen-specific CD8^+^ and CD4^+^ T cells secreting IFN-γ, granzyme B (GzB), or IL-4, which are involved in cellular immunity, was also significantly increased (Fig. 4C, Supplementary Fig. S20). By analyzing the spleen, lung, and serum, we further established that SE(Trojan-TLR7/8a) elicits not only local but also systemic immune responses (Fig. 4D). SE(Trojan-TLR7/8a) also had a notable effect on the spleen by enhancing GC B, T_FH_, and antigen-specific cytokine-secreting T-cell responses and increasing plasma and memory B-cell responses (Fig. 4E, F; Supplementary Figs. S21 and 22). We analyzed effector memory (CD44^+^ CD62L^−^) T cells that were primed for an immediate response to pathogens only in the lungs and detected them at the highest abundance in the SE(Trojan-TLR7/8a)-vaccinated group (Fig. 4G, Supplementary Fig. S23). Additionally, the antigen-specific T-cell responses in the lungs paralleled those in the LNs and spleens (Fig. 4H, Supplementary Fig. S24). OVA-specific IgG antibody titers, including those of IgG1- and IgG2c, which are indicative of Th2- or Th1-biased responses, respectively, were also highest in the serum of the SE(Trojan-TLR7/8a)-vaccinated group (Fig. 4I, Supplementary Fig. S25**)**. Taken together, these results demonstrated that prime-boost immunization with SE(Trojan-TLR7/8a) effectively induced robust localized and systemic humoral and cellular immune responses.

### A comparative study of commercialized vaccine formulations

#### Comparison with vaccine adjuvants

To further evaluate the clinical translational potential of SE(Trojan-TLR7/8a), we compared it with that of the FDA-approved vaccine adjuvants Alum and AS03. C57BL/6 mice were immunized with alum, AS03, or SE(Trojan-TLR7/8a) mixed with OVA twice at 3-week intervals. The performance of SE(Trojan-TLR7/8a), a squalene-based nanoemulsion incorporating TLR7/8a, was compared with that of AS03, which enhances cellular immunity by adding α-tocopherol to squalene-based emulsions (Fig. 4J–L) [23, 24]. Compared with alum, which is known to induce a strong humoral response, SE(Trojan-TLR7/8a) increased the proportion of humoral immune cells (T_FH_, GC B, and memory B cells) in the iLNs (Fig. 4J–L). SE(Trojan-TLR7/8a) also elicited higher levels of antigen-specific cytokine-secreting T cells and antigen-specific antibody responses, especially IgG2c, than did AS03, highlighting the potential benefit of TLR7/8a integration (Fig. 4M, N).

#### Comparison with the mRNA vaccine

**Fig. 5:**
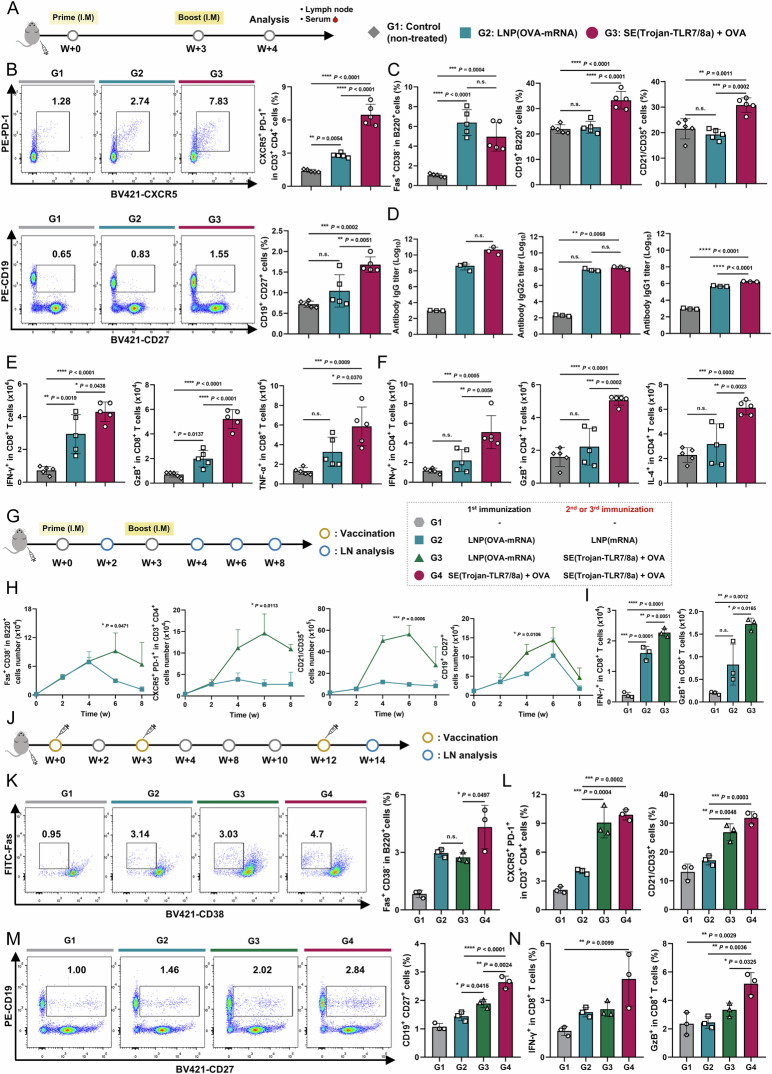
Compared with the mRNA vaccine, SE(Trojan-TLR7/8a) elicits a high-magnitude immune response. **A** C57BL/6 mice were intramuscularly immunized twice at 3-week intervals, after which the iLNs and serum were analyzed according to the schedule. The experimental groups included a nontreated control group and mice receiving LNP (OVA-mRNA) (OVA-mRNA 5 μg) or SE(Trojan-TLR7/8a) (79.5 nmol) with OVA (20 μg). **B** Representative flow cytometry plots and percentages of T_FH_ cells and memory B cells in the iLNs (*n* = 5 mice per group). **C** Percentages of GC B cells, B cells (CD19^+^ B220^+^), and FDCs (CD21/35^+^) in iLNs (*n* = 5 mice per group). **D** Serum OVA-specific IgG, IgG2c, or IgG1 titers (*n* = 3 mice per group). Total numbers of antigen-specific CD8^+^ (**E**) and CD4^+^ (**F**) T cells secreting IFN-γ, GzB, TNF-α, or IL-4 in the iLNs (*n* = 5 mice per group). **G**–**I** C57BL/6 mice were intramuscularly immunized with a homologous (LNP (OVA-mRNA)) or heterologous (SE(Trojan-TLR7/8a)) booster, and the iLNs were analyzed according to the schedule (*n* = 3 mice per group). **H** Kinetics of GC B cells, T_FH_ cells_,_ FDCs, and memory B cells in the iLNs. **I** Total number of antigen-specific CD8^+^ T cells secreting IFN-γ or GzB in the iLNs at 6 weeks. **J**–**N** C57BL/6 mice were intramuscularly immunized three times, and the iLNs were analyzed according to schedule (*n* = 3 mice per group). Representative flow cytometry plots and percentages of GC B cells (**K**) and percentages of T_FH_ cells and FDCs (**L**) in the iLNs at 12 weeks. **M** Representative flow cytometry plots and percentages of memory B cells in iLNs. **N** Percentage of antigen-specific CD8^+^ T cells secreting IFN-γ or GzB. The data are presented as the means ± s.d. In **B**–**F**, **I**, **K**–**N**, analysis was performed via one-way ANOVA with Tukey’s multiple comparison test; in **H**, analysis was performed via two-way ANOVA with Tukey’s multiple comparison test; *P* values are indicated (n.s., not significant; *P < 0.05, **P < 0.01, ***P < 0.001, ****P < 0.0001)

Next, the immune response elicited by SE(Trojan-TLR7/8a) was compared with that elicited by an mRNA vaccine, which was recently clinically approved for use as a SARS-CoV-2 vaccine. C57BL/6 mice were immunized with SE(Trojan-TLR7/8a) mixed with OVA or LNP (OVA-mRNA) twice at 3-week intervals (Fig. 5A). SE(Trojan-TLR7/8a) also significantly enhanced the immune defense system by markedly increasing the proportion of immune cells involved in the humoral response (T_FH_, memory B cells, GC B, B cells, and FDCs) (Fig. 5B, C). OVA-specific antibody titers (IgG, IgG2c, and IgG1) were also significantly greater with SE(Trojan-TLR7/8a) (Fig. 5D). Furthermore, SE(Trojan-TLR7/8a) substantially increased the numbers of antigen-specific CD8^+^ and CD4^+^ T cells secreting IFN-γ, granzyme B (GzB), or IL-4, indicating a significant improvement in immune effectiveness over the mRNA vaccine (Fig. 5E, F).

Heterologous vaccination, also known as “mix-and-match” vaccination, involves administering a series of vaccines from different platforms to an individual [28]. Recent studies suggest that combining an mRNA vaccine with a vaccine based on a different platform can increase immunogenicity, broaden the spectrum of immune responses, and possibly improve the durability of protection compared with homologous vaccination strategies. We compared the immunological effects of homologous LNP (OVA-mRNA) vaccination with those of heterologous vaccination with SE(Trojan-TLR7/8a) after LNP (OVA-mRNA) (Fig. 5G). Notably, heterologous vaccination significantly increased the numbers of GC B, T_FH_, and memory B cells and FDCs for up to 6 weeks, whereas homologous vaccination peaked at 4 weeks (Fig. 5H). Additionally, the number of antigen-specific cytokine-secreting CD8^+^ T cells was significantly greater after heterologous vaccination (Fig. 5I). Compared with homologous vaccination, the heterologous vaccination approach with SE(Trojan-TLR7/8a) is a more promising strategy for enhancing long-term immunogenicity by improving the durability of the immune response. Nine weeks after the second immunization (Fig. 5J), significantly greater levels of key indicators of humoral and cellular responses, including GC B, T_FH_, and memory B cells and FDCs (Fig. 5K–M), and antigen-specific CD8^+^ T-cell responses were observed in the SE(Trojan-TLR7/8a) group (Fig. 5N). This finding underscores the superior immunogenicity of SE(Trojan-TLR7/8a), highlighting its potential to induce robust and comprehensive immune responses (Supplementary Fig. S26).

### Cross-protection against SARS-CoV-2 and influenza

**Fig. 6:**
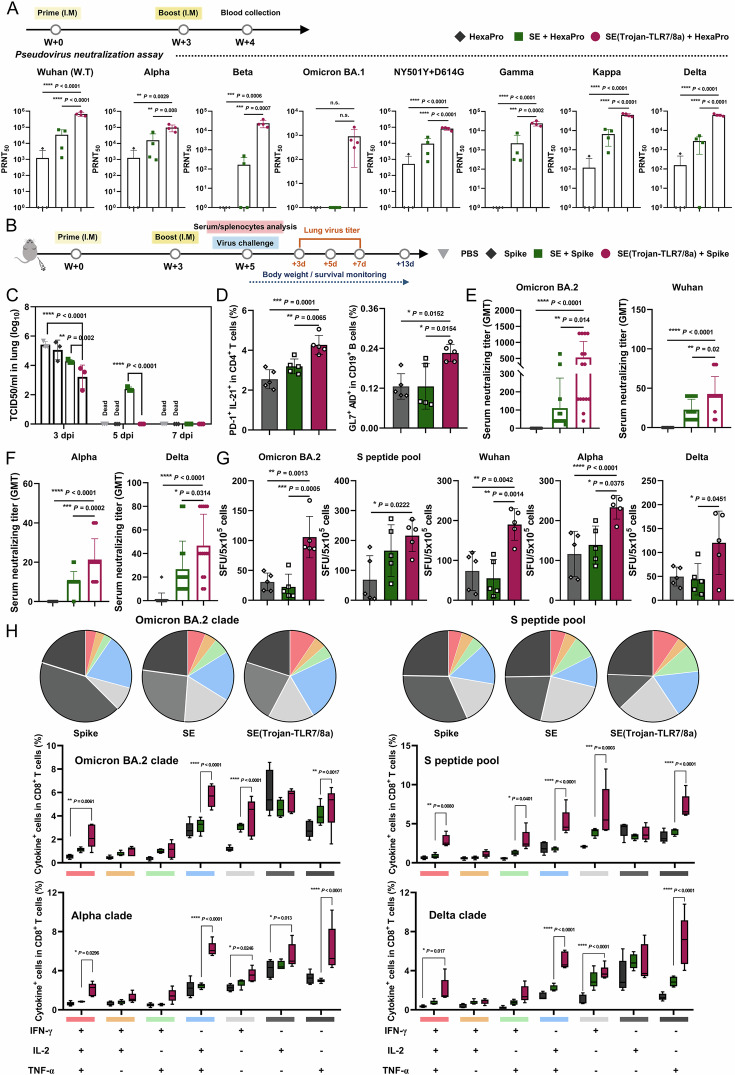
SE(Trojan-TLR7/8a) ensures cross-protection against SARS-CoV-2. **A** C57BL/6 mice were intramuscularly immunized with HexaPro (prime, 1 μg; boost, 5 μg) alone or combined with SE (with the same volume used for SE(Trojan-TLR7/8a)) or SE(Trojan-TLR7/8a) (79.5 nmol) twice at 3-week intervals, after which the serum was collected according to the same schedule (*n* = 4 mice per group). Neutralizing activity against SARS-CoV-2 variants was assessed via a microneutralization assay, which involved infecting HEK293T-ACE2 cells with pseudoviruses and analyzing serum samples collected 21 days after the initial immunization. **B**–**H** BALB/c mice were administered SARS-CoV-2 spike-stabilized trimer protein (spike protein, 0.5 μg) alone or in combination with SE or SE(Trojan-TLR7/8a) twice at 3-week intervals according to the schedule. **C** Mice were infected with 100 LD_50_ of the mouse-adapted SARS-CoV-2 virus, and infectious virus titration was confirmed from lung homogenates of the mice (*n* = 9 mice per group) at 3, 5, and 7 dpi. **D** Percentages of T_FH_ cells (PD-1^+^ IL-21^+^ in CD4^+^ T cells) and GC B cells (GL7^+^ AID^+^ in CD19^+^ cells) among splenocytes at 5 weeks (*n* = 5 mice per group). **E**, **F** A serum neutralization assay was performed against the Omicron BA.2, Wuhan, Alpha, and Delta strains, and the results are presented as the geometric mean titers (*n* = 15 mice per group). **G** IFN-γ spot-forming units (SFUs) were enumerated via ELISPOT assays after stimulating the splenocytes with the S peptide pool or inactivated with Omicron BA.2, Wuhan, Alpha, or Delta variant for 72 h. **H** Polyfunctional antigen-specific CD8^+^ T cells were analyzed by flow cytometry after stimulating the splenocytes with the S peptide pool or inactivated with Omicron BA.2, Alpha, or Delta variant in the presence of monensin for 12 h (*n* = 5 mice per group). The data are presented as the means ± s.d. In **A**, **D**–**G**, analysis was performed via one-way ANOVA with Tukey’s multiple comparison test; in **C**, **H**, analysis was performed via two-way ANOVA with Tukey’s multiple comparison test. *P* values are indicated (n.s., not significant; *P < 0.05, **P < 0.01, ***P < 0.001, ****P < 0.0001)

To verify that the promising SE(Trojan-TLR7/8a)-adjuvanted vaccine can effectively generate neutralizing antibodies and provide protection against SARS-CoV-2, the levels of these antibodies were measured via a pseudovirus neutralization assay. A mixture of SE(Trojan-TLR7/8a) and the HexaPro antigen, which is a prefusion-stabilized spike ectodomain, displayed excellent neutralizing antibody titers against all the variants tested (Fig. 6A) [29]. For further evaluation, we administered spike-stabilized trimers from the BA.2 variant as antigens to BALB/c mice, either alone or combined with SE or SE(Trojan-TLR7/8a), via intramuscular injection at weeks 0 and 3. Following the last immunization, the mice from each group were challenged with 100 LD_50_ of mouse-adapted SARS-CoV-2 (Wuhan strain) to assess immunogenicity and cross-protective responses (Fig. 6B). The groups receiving the spike protein with SE(Trojan-TLR7/8a) demonstrated complete protection against SARS-CoV-2 infection, as evidenced by the 100% survival rate and minimal body weight loss, in contrast to the spike protein-only group, which demonstrated 100% mortality within five days (Supplementary Fig. S27). By day 3 postchallenge, the SE(Trojan-TLR7/8a) group presented significantly reduced viral loads in the lungs, achieving complete viral clearance by day 5, in contrast to the persistent presence of viruses in the SE group (Fig. 6C). Additionally, the numbers of T_FH_ (PD-1^+^ IL-21^+^ in CD4^+^) and GC B cells (GL7^+^ AID^+^ in CD19^+^) were markedly greater in the SE(Trojan-TLR7/8a) group than in the other groups, indicating improved antibody production (Fig. 6D). Consequently, SE(Trojan-TLR7/8a) significantly increased neutralizing titers against both homologous (BA.2) and heterologous variants (Fig. 6E, F). Notably, the SE(Trojan-TLR7/8a) group exhibited superior CD8^+^ cytotoxic T lymphocyte (CTL) activity, which is essential for eradicating virus-infected cells, as evidenced by the increase in IFN-γ spot-forming units in response to homologous (BA.2) and heterologous (S peptide pool and inactivated SARS-CoV-2 variants) variants (Fig. 6G). Furthermore, the polyfunctional capacity of CTLs to clear the virus rapidly and provide long-term immunity was highest in the SE(Trojan-TLR7/8a) group (Fig. 6H, Supplementary Fig. S28). These findings underscore the powerful role of SE(Trojan-TLR7/8a) in amplifying both humoral and cellular immune defenses against SARS-CoV-2, allowing rapid clearance of viruses from the lung and acting as a significant enhancer of vaccine-induced protection against viruses.

**Fig. 7:**
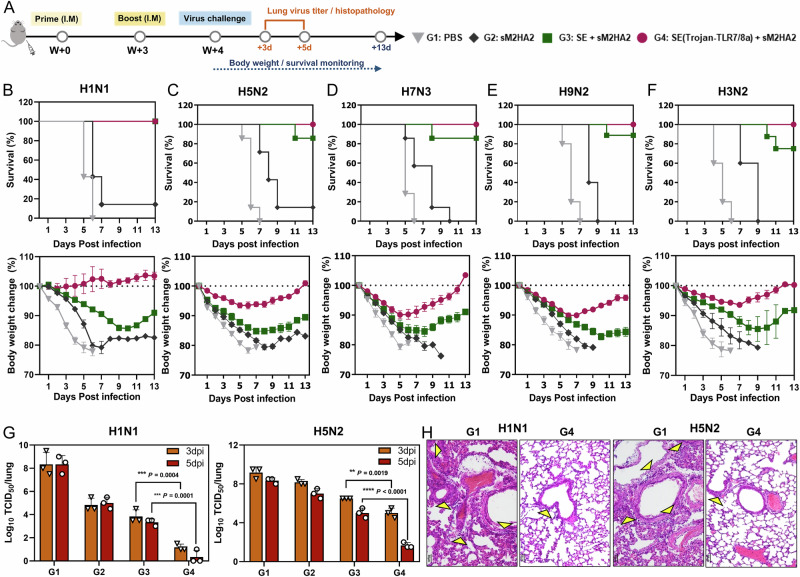
SE(Trojan-TLR7/8a) ensures cross-protection against influenza. **A** BALB/c mice were intramuscularly immunized with sM2HA2 (15 μg) alone or in combination with SE (with the same volume used for SE(Trojan-TLR7/8a)) or SE(Trojan-TLR7/8a) (79.5 nmol) twice in weeks 0 and 3 and then challenged with 10 LD_50_ of the mouse-adapted influenza subtypes one week after the second immunization according to the schedule. The survival rate (*upper panel*) and changes in body weight (*bottom panel*) after lethal infection with H1N1 (**B**), H5N2 (**C**), H7N3 (**D**), H9N2 (**E**), and H3N2 (**F**) were monitored for 13 days (*n* = 7 mice per group). **G** Lung virus titers were determined according to the TCID_50_ in MDCK cells at 3 and 5 days after H1N1 and H5N2 infection (*n* = 3 mice per group). **H** H&E-stained images of lung sections collected 5 days after H1N1 and H5N2 infection. The arrows indicate inflammatory cell infiltration. The data are presented as the means ± s.d. In **G**, analysis was performed via two-way ANOVA with Tukey’s multiple comparison test. *P* values are indicated (n.s. not significant; *P < 0.05, **P < 0.01, ***P < 0.001, ****P < 0.0001)

Next, we evaluated the efficacy of the SE(Trojan-TLR7/8a) adjuvant against influenza through intramuscular immunization in combination with sM2HA2, a promising antigen that targets a broad range of influenza subtypes and is composed of influenza matrix protein 2 (sM2) and the stalk domain of hemagglutinin protein (HA2) [30]. The sM2HA2 used in this study comprises the conserved extracellular (residues 1–24) and cytoplasmic (residues 44–97) domains of influenza sM2 from H5N1, H1N1, and H9N2 subtypes, as well as the stalk domain (residues 15-137) of HA2 from the A/EM/Korea/W149/06 (H5N1) strain [31]. Immunization with SE(Trojan-TLR7/8a) has been found to elicit sM2HA2-specific humoral and cell-mediated immunity, both locally and systemically, indicating potential protection against lethal influenza infection (Supplementary Figs. S29–31). To assess the protective ability of SE(Trojan-TLR7/8a), immunized mice were challenged with a 10 LD_50_ dose of various mouse-adapted influenza A virus subtypes (H1N1, H5N2, H7N3, H9N2, or H3N2) (Fig. 7A). Body weights and survival rates were then monitored until 13 days post infection (dpi) (Fig. 7B–F). All control mice died from infection between days 5 and 7, resulting in significant body weight loss of more than 20%. However, all the mice immunized with SE(Trojan-TLR7/8a) demonstrated complete protection against diverse influenza subtypes, with rapid reversal of body weight loss and notably, no weight loss upon challenge with H1N1. The virus titers in the lungs of the mice challenged with H1N1 or H5N2 were significantly lower in the SE(Trojan-TLR7/8a) group than in the control group within 5 dpi (Fig. 7G). Furthermore, in mice immunized with SE(Trojan-TLR7/8a), virus clearance from the lungs was correlated with histopathological analysis (Fig. 7H, Supplementary Fig. S32). Pathologic signs such as alveolar wall thickening and inflammatory cell infiltration (yellow arrows) were rarely observed in the SE(Trojan-TLR7/8a) group.

### Long-term protection against influenza and SFTS viruses

**Fig. 8:**
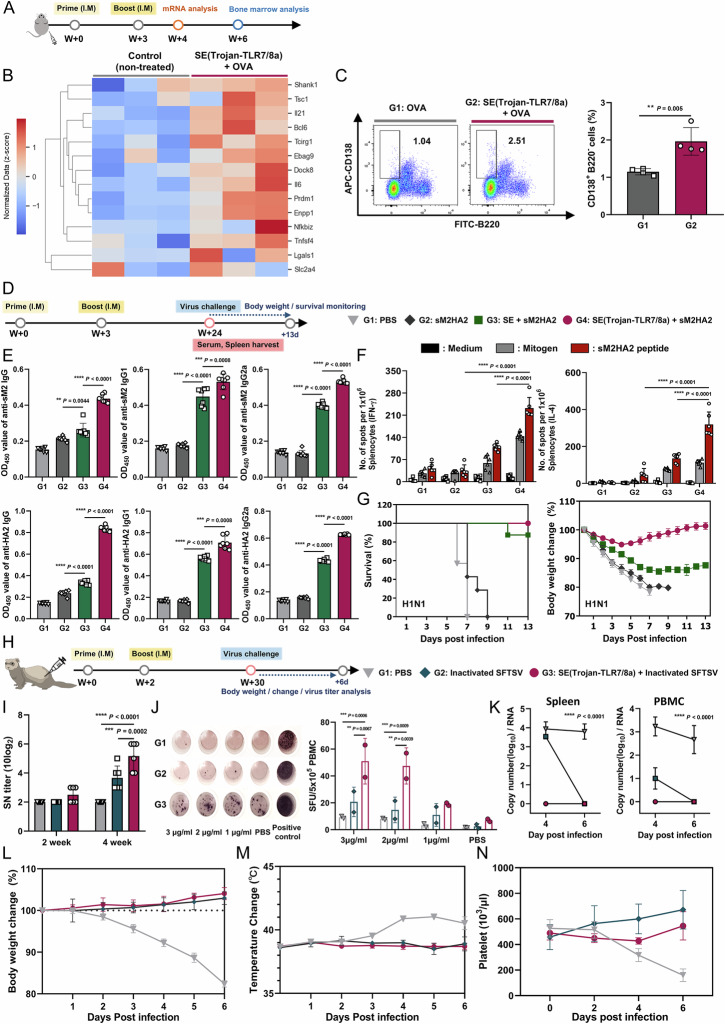
SE(Trojan-TLR7/8a) promotes long-term protective immune responses against influenza and SFTSV. **A** C57BL/6 mice were immunized intramuscularly with OVA (20 μg) alone or in combination with SE(Trojan-TLR7/8a) (79.5 nmol), and then the iLNs and bone marrow were analyzed according to the schedule. **B** Heatmap comparing the expression levels of genes related to the long-term immune response in independent isolations of LNs (*n* = 3 mice per group). **C** Representative flow cytometry plots and percentages demonstrating that SE(Trojan-TLR7/8a) increased the number of plasma cells (CD138^+^ B220^−^) in the bone marrow at 6 weeks postimmunization (*n* = 4 mice per group). **D**–**G** Long-lasting immune response and protective efficacy of SE(Trojan-TLR7/8a)-adjuvanted sM2HA2. BALB/c mice were immunized intramuscularly twice at 0 and 3 weeks and challenged with 10 LD_50_ of mouse-adapted H1N1 21 weeks after the second immunization according to the schedule. **E** Serum IgG, IgG1 and IgG2a antibody titers specific to sM2 or HA2 at a 1:100 serum dilution ratio 24 weeks after the first immunization (*n* = 7 mice per group). **F** The numbers of sM2HA2-specific IFN-γ SFU and IL-4 SFU were measured via an ELISPOT assay (*n* = 6 mice per group). **G** The survival rate (*left panel*) and percentage changes in initial body weight (*right panel*) after infection with the H1N1 virus were monitored for 13 days (*n* = 7 mice per group). **H**–**N** Aged ferrets were immunized intramuscularly with inactivated SFTSV alone or combined with SE(Trojan-TLR7/8a) (79.5 nmol) twice at 0 and 2 weeks and challenged with SFTSV 30 weeks after the initial immunization according to the schedule. **I** Serum neutralizing titers were analyzed at 2 and 4 weeks (*n* = 6 ferrets per group). **J** Representative image and numerical value of IFN-γ SFU in PBMCs, analyzed at 30 weeks, confirmed by an ELISPOT assay (*n* = 2 ferrets per group). **K** On days 4 and 6 after SFTSV infection, the spleen and PBMCs were collected, and the viral load was quantified via qRT‒PCR (*n* = 3 ferrets per day per group). **L**–**N** Changes in ferret body weight (**L**), body temperature (**M**), and platelet count (**N**) were measured until 6 dpi (*n* = 6 for days 1–4 and *n* = 3 for days 5–6). The data are presented as the mean ± s.d. In **E**, analysis was performed via one-way ANOVA with Tukey’s multiple comparison test; in **F**, **I**–**K**, analysis was performed via two-way ANOVA with Tukey’s multiple comparison test; in **C**, analysis was performed via a two-tailed unpaired *t* test. *P* values are indicated (n.s., not significant; *P < 0.05, **P < 0.01, ***P < 0.001, ****P < 0.0001)

To determine whether SE(Trojan-TLR7/8a) can sustain enhanced immune responses, we conducted mRNA sequencing to observe the upregulation of genes associated with long-term immunity, including genes related to the proliferation and differentiation of memory T cells (Tsc1), GC B cells (Bcl6 and Il21), and plasma cells (Prdm1, Lgals1, and Nfkbiz) (Fig. 8A, B) [32]. Given that GC B cells play a crucial role in generating sustained and high-quality antibody responses, we sought to assess whether SE(Trojan-TLR7/8a), which enhances GC B-cell responses, also facilitates differentiation into long-lived plasma cells (LLPCs) and influences sustained antigen-specific antibody secretion. Differentiated LLPCs migrate to the bone marrow, where they reside, produce antibodies, and maintain serum antibody levels against pathogens. At six weeks post-immunization, a significant increase in the LLPC population was observed in the bone marrow of the SE(Trojan-TLR7/8a)-immunized mice (Fig. 8A, C).

To assess the effectiveness of a vaccine, evaluating its ability to elicit durable immune responses and provide sustained protection against pathogens is crucial. BALB/c mice were immunized with SE(Trojan-TLR7/8a)-adjuvanted sM2HA2 at 3-week intervals (Fig. 8D). Twenty-four weeks after the initial immunization, the measured serum IgG levels and ELISPOT results for IFN-γ and IL-4 in splenocytes, all of which were specific to sM2HA2, sM2, or HA2, demonstrated that SE(Trojan-TLR7/8a) induces long-lasting humoral and cellular immune responses (Fig. 8E, F; Supplementary Figs. S33, 34). At that point, the mice were challenged with H1N1, after which the mice in the SE(Trojan-TLR7/8a) group fully recovered, with a 100% survival rate for 13 days, which was comparable to the survival rate immediately following immunization (Fig. 8G).

SFTSV is associated with increased morbidity and mortality in elderly patients [33]. Similarly, aged ferrets (≥3 years old) infected with SFTSV exhibit mortality with clinical symptoms resembling those observed in human cases [34, 35]. To evaluate the efficacy of the SE(Trojan-TLR7/8a) vaccine, aged ferrets (3 years old), an established immunocompetent model for SFTSV infection, were intramuscularly administered an inactivated SFTSV vaccine with or without SE(Trojan-TLR7/8a) twice at two-week intervals (Fig. 8H). Serum was collected two weeks after each immunization to assess neutralizing antibody (SN) production, with the highest SN titer observed with SE(Trojan-TLR7/8a) after boosting (Fig. 8I). At 30 weeks postpriming, the results of the IFN-γ ELISPOT assays revealed stronger SFTSV-specific T-cell responses in the SE(Trojan-TLR7/8a) and vaccine combination groups than in the vaccine alone group (Fig. 8J). To evaluate the protective efficacy of SE(Trojan-TLR7/8a), ferrets were challenged with a lethal dose (10^6^ TCID_50_) of the CB1/2014 strain. Viral RNA was present in all unvaccinated ferret tissues at 4 and 6 dpi but was undetectable in all animals in the SE(Trojan-TLR7/8a) vaccine group, indicating a faster rate of virus clearance (Fig. 8K, Supplementary Fig. S35). Additionally, the ferrets were monitored for clinical signs of infection, changes in body weight and temperature, platelet count, and serum neutralization (SN) titers until 6 dpi (Supplementary Fig. S35). The unvaccinated group presented high fever, 20% body weight loss, and severe thrombocytopenia. In contrast, the SE(Trojan-TLR7/8a) group presented no clinical symptoms (Fig. 8L–N). Overall, the SE(Trojan-TLR7/8a) vaccine enhanced SFTSV-specific T-cell responses, providing sufficient protection against lethal challenges and enabling rapid virus clearance even after long-term vaccination.

## Conclusion

Here, we propose a novel vaccine adjuvant design that can induce robust protective immunity against heterologous variants of pandemic viruses such as influenza virus, SARS-CoV-2 and endemic SFTSV via multiscale (i.e., macroscopic and cellular level) dynamic immunomodulation that effectively induces GC B cells and polyfunctional T cells. We developed an engineered nanoadjuvant, SE(Trojan-TLR7/8a), by incorporating Trojan-TLR7/8a, which can activate APCs in a time-dependent manner, into a clinically approved squalene-based nanoemulsion that can control the kinetics of vaccine delivery at the macroscopic level. We found that the controlled delivery of the vaccine components in the SE(Trojan-TLR7/8a)-adjuvanted vaccine improved the locoregional attraction of innate immune cells and enhanced the cellular uptake of the vaccine. At the cellular level, timely activation of APCs via Trojan-TLR7/8a induces nonexhausted APCs, which secrete IL-12(p70) in a sustained manner, enhancing the migratory capacity of APCs into the LNs. Furthermore, the SE(Trojan-TLR7/8a) and antigens also migrated to the LNs directly in a sustained and continuous delivery manner. Multiscale dynamic immunomodulation by the SE(Trojan-TLR7/8a) adjuvant thereby enhances both humoral and cellular responses, including increased activity of T_FH_ cells and GC B cells and antigen-specific polyfunctional T-cell responses. We selected TLR7/8a for its multifaceted immunomodulatory functions to trigger innate immune responses and enhance T-cell-mediated immunity, which are crucial for eliminating infectious viruses, as well as activating T_FH_ cells, which are essential for stimulating GC B cells, thereby promoting high-affinity antibody production and fostering long-lasting immune memory. However, the systemic toxicity induced by uncontrolled immune stimulation and immune tolerance induced by repeated bolus administrations of conventional TLR7/8a negates their benefits [36, 37]. Trojan-TLR7/8a reduces off-target and non-selective stimulation, reducing systemic toxicity, which is a major hurdle for the clinical translation of the small molecule TLR7/8a. Notably, Trojan-TLR7/8a also induced nonexhausted APCs, which enhanced the migration of APCs into LNs through the time-dependent activation of APCs.

We used the well-conserved recombinant fusion protein sM2HA2 as the influenza vaccine antigen for cross-protection against influenza A subtypes and the prefusion-stabilized spike ectodomain HexaPro or SARS-CoV-2 spike-stabilized trimer protein for the COVID-19 subunit vaccine. Although the use of conserved domains as protein antigens is promising for developing universal vaccines, their low immunogenicity limits protection against heterologous virus variants, making the choice of adjuvant crucial for effectively enhancing and targeting the immune response. SE(Trojan-TLR7/8a) evoked broad and prolonged immunity against multiple influenza (H1N1, H5N2, H7N3, H9N2, and H3N2) and SARS-CoV-2 variants (Alpha, Beta, Wuhan, Delta, and Omicron BA.2) by activating GC B cells and polyfunctional T cells, leading to 100% protection and full body weight recovery postimmunization. Notably, immunization with SE(Trojan-TLR7/8a) also enhanced SFTSV-specific T-cell responses, providing sufficient protection against lethal challenges and enabling rapid virus clearance even after long-term vaccination in aged ferrets with immune systems similar to those of humans. The results suggested that SE(Trojan-TLR7/8a) can be used as a potent adjuvant for vaccination against various infectious diseases that are associated with increased morbidity and mortality in aged patients [29].

The ability of SE(Trojan-TLR7/8a) to promote antigen-specific CD8^+^ and CD4^+^ T-cell responses and enhance humoral immunity—including T_FH_ cell and GC B-cell activity—highlights its potential for clinical translation and its ability to contribute significantly to advancements in the field of vaccination. Comparative analysis with other FDA-approved vaccine adjuvants, such as alum and AS03, as well as with the mRNA vaccine, also demonstrated the superior ability of SE(Trojan-TLR7/8a) to induce both humoral and cellular immune responses. Our SE(Trojan-TLR7/8a)-adjuvanted subunit vaccine against SARS-CoV-2 using premade and safe proteins as antigens should be distinguished from Covaxin®, which uses whole inactivated virus as an antigen and alum-imidazoquinoline as an adjuvant authorized for human use [38]. Additionally, lyophilizing SE(Trojan-TLR7/8a) addresses critical challenges in vaccine storage and distribution by improving stability and extending shelf-life. Unlike mRNA vaccines, which often require ultralow temperatures during storage and transportation, the lyophilized form of SE(Trojan-TLR7/8a) can withstand a wider range of temperatures, making it less reliant on stringent cold chain requirements [39]. We believe that the concept of the SE(Trojan-TLR7/8a) vaccine platform, which evokes GC B cells and polyfunctional T cells via multiscale dynamic immunomodulation, can be used for the development of future vaccines to induce robust protective immunity against emerging heterologous variants of pandemic and endemic viruses [6, 26].

## Experimental section

### Fabrication of SE(Trojan-TLR7/8a)

All squalene-based nanoemulsions (SEs) were fabricated via a microtip probe sonicator (VCX 750, Sonics & Materials) for 1 min to emulsify the water and oil phases. For the fabrication of SE(Trojan-TLR7/8a), an SE loaded with a cholesterol-conjugated Toll-like receptor 7/8 agonist (TLR7/8a) (Trojan-TLR7/8a), 2 mg of Trojan-TLR7/8a (0.1% (w/v)), squalene (4.3% (w/v)), or Span-85 (0.5% (w/v)) was dissolved in 1 ml of chloroform. The mixture was then evaporated under vacuum in a desiccator to completely remove the chloroform. The resulting solution was dispersed in PBS or citrate buffer (Sigma), and Tween-80 (0.5% (w/v)) was added to obtain a final volume of 2 ml. SE was generated via the sonication of squalene (4.3% (w/v)), Span-85 (0.5% (w/v)), and Tween-80 (0.5% (w/v)) in a PBS solution to obtain a final volume of 2 ml. SE-R848 was fabricated via the same method as SE, but 10 mg of oleic acid was added. In the case of SE + R848, the two components of SE and R848 were simply mixed.

### Animals

The animal experiments were performed in full compliance with the outlines of the Guide for the Care and Use of Laboratory Animals of the National Institutes of Health (NIH). The animal study was reviewed and approved by the Institutional Animal Care and Use Committee (IACUC) of the Sungkyunkwan University School of Medicine (SKKUIACUC2022-05-09-1), Chungnam National University (202307A-CNU-118), the Osong Medical Innovation Foundation (KBIO-IACUC-2023-07), the Institute for Basic Science (IBS-2023-043), and Chungbuck National University (CBNUA-2130-23-02), which is accredited by the Association for Assessment and Accreditation of Laboratory Animal Care International (AAALAC International) and abides by the Institute of Laboratory Animal Resources (ILAR) guidelines. C57BL/6 and BALB/c female mice (6–8 weeks old) were purchased from Orient Bio or Hanil Laboratory Animal Center. All the animals were housed individually in well-ventilated cages under conditions of 30–70% humidity, 21–26 °C temperature, and a 12/12-h light/dark cycle, and the ferrets were housed in an animal biosafety level (ABSL3) facility at Chungbuk National University (Cheongju, South Korea). Every effort was made to minimize animal suffering and to use the minimum number of animals necessary to obtain valid scientific results. All the mice were treated humanely, and their welfare was monitored throughout the study.

### In vitro Transwell migration assay

The migratory ability of SE or SE(Trojan-TLR7/8a)-treated BMDCs was assessed through a migration assay using a 6-well Transwell^®^ with an 8.0 μm pore membrane (Thermo Fisher). BMDCs were plated in 6-well cell culture plates (1 × 10^6^ cells per well) and then treated with SE, SE + R848 (1 μg ml^−1^, 3.18 μM), or SE(Trojan-TLR7/8a) (2.9 μg ml^−1^, 3.18 μM), each mixed with OVA (10 μg ml^−1^), for 12 h before the migration assay. After 12 h, the BMDCs were harvested from the 6-well cell culture plates, and 4 × 10^5^ cells per well were plated into Transwell inserts. The chemoattractants CCL21 (50 ng ml^−1^, PeproTech) and CCL19 (50 ng ml^−1^, PeproTech) were added to the bottom chamber. Following incubation for 24 h, the number of BMDCs that migrated to the bottom chamber was counted via a Countess^TM^ II (Invitrogen).

### Toxicity analysis

Serum analysis C57BL/6 female mice (6 weeks old) were immunized intramuscularly with SE, SE + R848 (25 μg, 79.5 nmol), or SE(Trojan-TLR7/8a) (72.1 μg, 79.5 nmol), each mixed with OVA (20 μg) in the hind leg. Blood samples were collected at 1, 2, 4, 6, 8, 12, and 24 h following immunization and centrifuged at 10,000 × *g* for 10 min to separate the serum. The serum samples were analyzed via AST or ALT activity analysis kits (Sigma‒Aldrich) according to the manufacturer’s instructions. For analysis of cytokine levels in the serum, IL-6 and TNF-α were quantified via OptEIA ELISA kits (BD Biosciences) according to the manufacturer’s instructions.

### In vivo cell recruitment into muscle

C57BL/6 female mice (6 weeks old) were immunized intramuscularly with SE (with the same volume used for SE(Trojan-TLR7/8a)), SE + R848 (25 μg, 79.5 nmol) or SE(Trojan-TLR7/8a) (72.1 μg, 79.5 nmol), each mixed with OVA (20 μg) in the hind leg. The injected thigh muscles were excised on day 1. For flow cytometry analysis, single cells were labeled with the fixable viability dye eFluor™ 780 (1:2000, eBioscience). The cells were stained with PerCP/Cy5.5 anti-mouse/human CD11b (clone: M1/70), BV421 anti-mouse CD11c (clone: N418), Alexa 647 anti-mouse Ly6G (clone: 1A8), APC-anti-mouse CD4 (clone: GK1.5), BV510-anti-mouse CD3 (17A2), BV421-anti-mouse NK-1.1 (PK136), PE anti-mouse CD8α (clone: 53-6.7) (BioLegend), and PE anti-mouse Ly6C (clone: AL-21) (BD Biosciences) antibodies. Flow cytometry data were analyzed via BD FACSCanto™ II (The BIORP of the Korea Basic Science Institute (KBSI)) and quantified via FlowJo v.10 software. The gating strategy used is provided in Supplementary Fig. 5.

### Immunofluorescence staining of mouse iLN sections

C57BL/6 female mice (6 weeks old) were immunized intramuscularly with SE (with the same volume used for SE(Trojan-TLR7/8a)), SE + R848 (25 μg, 79.5 nmol), or SE(Trojan-TLR7/8a) (72.1 μg, 79.5 nmol), each mixed with OVA (20 μg, Invitrogen) in the hind leg. For immunofluorescence imaging of iLN sections, the iLNs were collected 5 days after immunization. Cryostat sections embedded in iLNs were fixed in 4% paraformaldehyde (Biosesang) for 24 h and equilibrated in 30% sucrose (Sigma‒Aldrich) solution for an additional 24 h. After the iLNs were frozen at −80 °C, the iLNs were sectioned at a thickness of 10 μm via a Cryostar NX50 (Thermo Scientific). The sectioned tissues were placed on poly-L-lysine-coated glass slides and washed three times with PBS. The tissues were subsequently blocked with a 5% fetal bovine serum (FBS, HyClone) solution for 1 h. After three washes with PBS, the sections were stained with Alexa Fluor 488-conjugated anti-mouse CD3 (clone: 17A2) and Alexa Fluor 647-conjugated anti-mouse/human GL7 antigen (clone: GL7) (BioLegend) antibodies and incubated overnight at 4 °C. Following a final wash, the slides were mounted with mounting medium (Dako) and coverslips. The iLN sections were subjected to confocal laser scanning microscopy (CLSM) with a Leica TSC SP8 instrument equipped with a 20× objective (Leica Microsystems) (the BIORP of the Korea Basic Science Institute (KBSI)).

### Mouse immunization

C57BL/6 female mice (6 weeks old) were immunized intramuscularly with SE (with the same volume used for SE(Trojan-TLR7/8a)) or SE(Trojan-TLR7/8a) (72.1 μg, 79.5 nmol), each mixed with OVA (20 μg) in the hind leg at week 0 and boosted at week 3. iLNs, the spleens, and the lungs were dissected on day 7 after boosting immunization.

### Humoral response in iLNs and spleens

For flow cytometry analysis, single cells were labeled with the fixable viability dye eFluor™ 780 (1:2000, eBioscience) and stained with FITC anti-mouse Fas (CD95) (clone: SA367H8), BV421 anti-mouse CD38 (clone: 90), PE anti-mouse CD19 (clone: 1D3/CD19), APC/FITC anti-mouse/human CD45R/B220 (clone: RA3-6B2), APC anti-mouse CD138 (clone: 281-2), PE anti-mouse CD19 (clone: 1D3/CD19), BV421 anti-mouse/rat/human CD27 (clone: LG.3A10), APC anti-mouse CD21/CD35 (clone: 7E9), BV421 anti-mouse CXCR5 (clone: L138D7), PE anti-mouse PD-1 (clone: 29 F.1A12), BV510 anti-mouse CD3 (clone: 17A2), and APC anti-mouse CD4 (clone: GK1.5) (BioLegend) antibodies. Flow cytometry data were analyzed via BD FACSCanto™ II (The BIORP of the Korea Basic Science Institute (KBSI)) and quantified via FlowJo v.10 software. The gating strategy used is provided in Supplementary Fig. 18.

### Cellular response in iLNs, spleens, and lungs

For antigen-specific T-cell analysis, single cells (5 × 10^5^ cells per well) were plated in a round-bottom 96-well plate. The cells were then restimulated with OVA_257-264_ peptide for CD8^+^ T cells or OVA_323-339_ peptide (ISQAVHAAHAEINEAGR) for CD4^+^ T cells (10 μg ml^-1^, MIMOTOPES), IL-2 (30 ng ml^−1^, PeproTech), or GolgiPlug (protein transport inhibitor, 0.6 μg ml^-1^, BD Bioscience) for 12 h. After stimulation, the cells were collected and washed once. The cells were labeled with fixable viability dye eFluor™ 780 (1:2000, eBioscience) and stained with surface markers, including APC-conjugated anti-mouse CD4 (clone: GK1.5), BV510-conjugated anti-mouse CD3 (clone: 17A2), and PE-conjugated anti-mouse CD8a (clone: 53-6.7, BioLegend) antibodies, for 30 min at 4 °C. Then, the cells were washed and permeabilized with fixation/permeabilization solution for 20 min at 4 °C. For intracellular staining, fixed/permeabilized cells were washed once with BD Perm/Wash buffer (BD Biosciences) and stained with BV421 anti-human/mouse Granzyme B (clone: QA18A28), FITC anti-mouse IFN-γ (clone: XMG1.2), and PE anti-mouse IL-4 (clone: 11B11) (BioLegend) antibodies for 30 min at 4 °C. Flow cytometry data were analyzed via BD FACSCanto™ II (The BIORP of the Korea Basic Science Institute (KBSI)) and quantified via FlowJo v.10 software.

### Phenotypic and functional analysis of T cells in lungs

For analysis of T cells, cells from the lungs were collected and prepared as single-cell suspensions. Single cells were labeled with the fixable viability dye eFluor™ 780 (1:2000, eBioscience) and stained with PerCP anti-mouse CD3 (clone: 145–2C11), APC anti-mouse CD4 (clone: GK1.5), PE anti-mouse CD8a (clone: 53–6.7), BV510 anti-mouse/human CD44 (clone: IM7), and BV421 anti-mouse CD62L (clone: MEL-14) (BioLegend) antibodies. Flow cytometry data were analyzed via BD FACSCanto™ II (The BIORP of the Korea Basic Science Institute (KBSI)) and quantified via FlowJo v.10 software. The gating strategy used is provided in Supplementary Fig. 23.

### mRNA‒lipid nanoparticle (LNP) vaccine formulation and characterization

LNP (OVA-mRNA) was synthesized via a microfluidic device (NanoAssemblr® Ignite). An aqueous phase containing 1-methylpseudoridine (m1ψ)-modified OVA mRNA (TriLink, L7610) and an ethanol phase containing lipid and cholesterol components were mixed at a flow rate ratio of 3:1 and a lipid/RNA weight ratio of 10:1 in a microfluidic device. Briefly, the aqueous phase was prepared with 10 mM citrate buffer and m1ψ-modified OVA mRNA (0.1 mg ml^-1^). To prepare the ethanol phase, ALC-0315 (ionizable lipid) (MCE), ALC-0159 (PEGylated lipid) (MCE), 1,2-distearoyl-*sn*-glycero-3-phosphocholine (Avanti Polar Lipid), and cholesterol (Sigma‒Aldrich) were formulated into LNP (OVA-mRNA). Following the mixing step, LNP (OVA-mRNA) was dialyzed against 1× PBS for 16 h via a 10 kDa MWCO filter (Thermo Fisher). The efficiency of mRNA encapsulation was determined via a modified Quant-iT RiboGreen RNA assay (Invitrogen).

### Influenza viruses and challenge test

BALB/c female mice (6 weeks old) were immunized intramuscularly with SE (with the same volume used for SE(Trojan-TLR7/8a)) or SE(Trojan-TLR7/8a) (72.1 μg, 79.5 nmol), each mixed with sM2HA2 (15 μg) in the hind leg at week 0 and boosted at week 3. The mice were infected with mouse-adapted pathogenic avian influenza A subtype viruses, namely, A/Puerto Rico/8/34 (H1N1), A/Aquatic bird/Korea/W81/2005 (H5N2), A/Aquatic bird/Korea/W44/2005 (H7N3), A/Chicken/Korea/116/2004 (H9N2) or A/Philippines/2/2008 (H3N2). The viruses utilized in this study were generously provided by Dr. Young-Ki Choi (College of Medicine and Medical Research Institute, Chungbuk National University, Cheongju, Republic of Korea). One week after the boost immunization, the mice were anesthetized via an intraperitoneal immunization of ether and infected intranasally with 10 LD_50_ of the above viruses in 20 μl of PBS. The mice were monitored for 13 days at fixed time points to measure weight loss and survival. The survival rate was determined by death or at a cutoff of 20% body weight loss, at which point the animals were humanely euthanized via CO_2_ inhalation for 5 min, and all surviving mice were euthanized via this method.

### Lung histopathological analysis

For lung histopathology analysis, lung tissues were collected from the mice at 5 days post infection and fixed in a 10% neutral buffered formalin solution for 2 days on a shaker. The samples were subsequently embedded in paraffin wax, and 4–6 μm thick sections were prepared via a microtome. The sections were then mounted on slides and stained with hematoxylin and eosin. Histopathological changes were examined via light microscopy.

### Pseudovirus neutralization assay

Derivatives of 293T cells expressing ACE2 were generated by transducing 293 T cells with an ACE2 expression lentiviral vector (Addgene plasmid, 145839). Cells from a single-cell clone were derived by limiting dilution from the bulk populations. These 293T-ACE2 cells were seeded at a density of 2 × 10^4^ cells per well in 96-well luminometer-compatible tissue culture plates (SPL Life Science) 24 h before infection. For the neutralization assay, pseudoviruses (1.8 × 10^7^ copies) were mixed with serum diluted 50–984,150-fold and then added to the 293T-ACE2 cells. After incubation for 24 h, the inoculum was replaced with fresh medium, and luciferase activity was measured 72 h post infection. Briefly, the cells were lysed with 40 μl of Passive Lysis Buffer (Promega) per well. Luciferase activity in the lysates was measured via the Nano-Glo Luciferase Assay System (Promega). Specifically, 40 μl of the substrate in Nano-Glo buffer was mixed with 40 μl of cell lysate and incubated for 3 min at room temperature. NanoLuc luciferase activity was then measured via a GloMax® Navigator Microplate Luminometer (Promega) with a 300 ms integration time. The 50% neutralization titer (NT_50_) values were calculated via nonlinear regression (log[inhibitor] vs. response, with three parameters) in GraphPad Prism 9 (GraphPad Software, Inc.).

### SARS-CoV-2 challenge experiment

C57BL/6 female mice (6 weeks old) were immunized intramuscularly with SE (with the same volume used for SE(Trojan-TLR7/8a)) or SE(Trojan-TLR7/8a) (72.1 μg, 79.5 nmol), each mixed with SARS-CoV-2 spike-stabilized trimer protein (0.5 μg) in the hind leg at week 0 and boosted at week 3. Serum and splenocytes were collected 2 weeks after immunization. Two weeks after immunization, the mice were challenged intranasally with 100 LD_50_ of mouse-adapted SARS-CoV-2, and the survival rate and body weight were monitored for up to 7 days. Mice that lost more than 25% of their body weight reached the experimental endpoint. To examine the infectious viral titers in the virus-infected lungs, the lungs of the challenged mice were harvested and frozen at −80 °C at 3, 5, and 7 days after infection. Homogenates were prepared from the frozen tissues via TissueLyser II (Qiagen) and centrifuged at 15000 × g for 10 min. Vero-E6 cells were treated with serially diluted 10-fold homogenates for 1 h at 37 °C. The homogenates were removed and incubated with DMEM supplemented with 2% FBS for 72 h at 37 °C. Cytopathic effects (CPEs) were monitored at 3 dpi, and the viral titers were calculated via the Reed–Muench method and expressed as TCID_50_.

### Ferret immunizations and viral challenge

For immunization experiments, 3-year-old male ferrets were confirmed to have no preexisting antibodies against SFTSV. Naïve aged ferrets were immunized intramuscularly with PBS (300 µl) or the inactivated SFTSV (30 µg) vaccine alone or in combination with SE(Trojan-TLR7/8a) (79.5 nmol, 300 µl). Vaccines were administered twice, with a two-week interval, and serum was collected at 2-week intervals until 4 weeks after each first immunization. The ferrets were subsequently challenged with SFTSV (CB1/2014) intramuscularly at 10^6.0^ TCID ml^−1^ at 30 weeks after the initial immunization. Clinical signs of infection―viral load, platelet counts, body weight, and body temperature―were monitored at days 0, 2, 4, and 6 postchallenge. Hematological parameters were analyzed via EDTA-treated whole blood samples with a Celltac hematology analyzer (MEK-6550J/K, Nihon Kohden). Serum was collected from each ferret at two-day intervals post infection, and peripheral virus titers were determined. Three animals per group were sacrificed on days 4 and 6 to collect tissue samples (liver, spleen, kidney, bone marrow, and PBMCs) via individual scissors. The viral loads in the collected tissues and serum were quantified via qRT-PCR. Viruses were handled in an enhanced biosafety level 3 (BSL3) containment laboratory as approved by the Korean Centers for Disease Control and Prevention (KCDC-14-3-07).

### Statistics and reproducibility

All the statistical analyses were performed via GraphPad Prism 8 and Microsoft Excel 2016. P values (n.s.: not significant, *P < 0.05, **P < 0.01, ***P < 0.001, and ****P < 0.0001) were used to indicate statistical significance. The detailed statistical analysis methods are included in the figure legends.

## Supplementary information

Supplementary Information
