# Author Correction: Multiscale dynamic immunomodulation by a nanoemulsified Trojan-TLR7/8 adjuvant for robust protection against heterologous pandemic and endemic viruses

**DOI:** 10.1038/s41423-025-01328-0

**Published:** 2025-08-08

**Authors:** Yeon Jeong Yoo, Suhyeon Kim, Asha Wickramasinghe, Jaemoo Kim, JuA Song, Young-Il Kim, Juryeon Gil, Young-Woock Noh, Min-Ho Lee, Sang-Seok Oh, Myeong-Mi Lee, Yebin Seong, Jong-Soo Lee, Young Ki Choi, Yong Taik Lim

**Affiliations:** 1 Department of Nano Science and Technology, Department of Nano Engineering, SKKU Advanced Institute of Nanotechnology (SAINT), School of Chemical Engineering, and Biomedical Institute for Convergence at SKKU, Sungkyunkwan University, Suwon, Republic of Korea; 2 College of Veterinary Medicine, Chungnam National University, Daejeon, Republic of Korea; 3 Center for Study of Emerging and Re-emerging Viruses, Korea Virus Research Institute, Institute for Basic Science (IBS), Daejeon, Republic of Korea; 4 College of Medicine and Medical Research Institute, Chungbuk National University, Cheongju, Republic of Korea; 5 New Drug Development Center, Osong Medical Innovation Foundation, Cheongju, Republic of Korea; 6 College of Pharmacy, Chungbuk National University, Cheongju, Republic of Korea

**Keywords:** Adjuvants, Immunotherapy, Infectious diseases

Correction to: *Cellular & Molecular Immunology* 10.1038/s41423-025-01306-6, published online 25 June 2025

In this article the author’s name Young Ki Choi was incorrectly written as Yong Ki Choi. The original article has been corrected.

