# Supplementary material for: Multiscale dynamic immunomodulation by a nanoemulsified Trojan-TLR7/8 adjuvant for robust protection against heterologous pandemic and endemic viruses
Source: Cell Mol Immunol. 2025 Jun 25;22(9):1045–60. doi: 10.1038/s41423-025-01306-6 (PMC12398497; doi:10.1038/s41423-025-01306-6)
Supplement: Supplementary file 1 — Supplementary Information [file 41423_2025_1306_MOESM1_ESM.pdf]

Supplementary Information

Multiscale Dynamic Immunomodulation by a Nanoemulsified Trojan-TLR7/8 Adjuvant for Robust Protection Against Heterologous Pandemic and Endemic Viruses

Yeon Jeong Yoo, Suhyeon Kim, Asha Wickramasinghe, Jaemoo Kim, JuA Song, Young-Il Kim, Juryeon Gil, Young-Woock Noh, Min-Ho Lee, Sang-Seok Oh, Myeong-Mi Lee, Yebin Seong, Jong-Soo Lee,, Yong Ki Choi*, and Yong Taik Lim**

Additional supplementary materials and methods

Lyophilization of SE(Trojan-TLR7/8a). For lyophilization of SE(Trojan-TLR7/8a) or SE, they were prepared by the same method, except that squalene was dispersed in a 10% (w/v) sucrose-PBS solution instead of PBS. After the fabrication process, these solutions were followed by a two-step freezing process. They were frozen at -80 °C for 12 h after fabrication (first freezing). Then, they were frozen in liquid nitrogen for 5 min (second freezing). After the steps of freezing, they were lyophilized in a freeze-dryer (FDU-2100, EYELA) for 12 h under the conditions of 10 Pa and at -80 °C. The lyophilized SE(Trojan-TLR7/8a) or SE was then reconstituted with distilled water before use.

To confirm whether lyophilization affected the immunostimulatory effect of SE(Trojan-TLR7/8a), C57BL/6 female mice (6 weeks old) were immunized intramuscularly with SE(Trojan-TLR7/8a) that was fresh or lyophilized at week 0 and boosted at week 3. After 1 week following boosting immunization, the blood samples were collected and centrifuged at 10,000g for 10 min to separate serum for ELISA assays to evaluate OVA-specific antibodies.

Characterization of SE(Trojan-TLR7/8a) (DLS, encapsulation efficiency, stability). The differential number of size and zeta potential of the formulations were measured using dynamic light scattering (DLS, ELS-Z electrophoretic light scattering photometer). The loaded amount of Trojan-TLR7/8 agonist in SE(Trojan-TLR7/8a) was quantified by measuring UV absorbance at 324 nm (ultraviolet-visible light spectrometry, UV-1800). To compare the structural stability of R848 and Trojan-TLR7/8 agonist, both SE-R848 and SE(Trojan-TLR7/8a) were centrifuged at 5,000g, for 20 min using a 10 kDa MWCO centrifuge filter (Amicon, Merck). The filtered solutions were then collected to quantify the amount of each drug released from the formulations.

Differentiation of Bone marrow-derived dendritic cells (BMDCs) and bone marrow-derived macrophages (BMDMs). BMDCs and BMDMs were generated from the bone marrow of C57BL/6 female mice (6 weeks old). The femurs and tibias were collected and flushed with RPMI1640 medium (Thermo Fisher), supplemented with 10% heat-inactivated fetal bovine serum (FBS, Hyclone) and 1% penicillin-streptomycin, using a 26-gauge syringe. The red blood cells (RBCs) were removed with RBC lysis buffer (BioLegend). For BMDCs, 2×10^6 cells were suspended in RPMI1640 medium containing mGM-CSF (20 ng ml^{-1}, CreaGene). After 3 days, fresh medium containing mGM-CSF (20 ng ml^{-1}) was added. After 6

days, 10 ml of medium was collected, centrifuged at 488g for 3 min, and supernatant was removed. Then, the remaining cells were resuspended in a fresh medium containing mGM-CSF (20 ng ml^{-1}). For BMDMs, 5×10^5 cells were suspended in Dulbecco's Modified Eagle's Medium (DMEM, Thermo Fisher) containing mM-CSF (20 ng ml^{-1}, CreaGene). After 3 days, fresh medium containing mM-CSF (20 ng ml^{-1}) was added. After 6 days, all medium in corning dishes were removed and fresh medium containing mM-CSF (20 ng ml^{-1}) was added. Finally, after 7 days, differentiated BMDCs and BMDMs were harvested and used.

In vitro BMDCs cellular uptake assay (Endocytosis uptake: CLSM, IL-12). For assessing the cellular uptake mechanism of SE(Trojan-TLR7/8a), DiD-loaded SE(Trojan-TLR7/8a) was fabricated following the method previously described. To encapsulate DiD in squalene, a solution containing 0.1 mg of DiD (Invitrogen) and squalene was dissolved in chloroform, followed by complete evaporation of the chloroform. Immature BMDCs (2.5×10^4 cells per well) were harvested and seeded in an ibidi µ-slide 8-well microscopy chamber. After 1 h preincubation with Dynasore (40 µM, Sigma-Aldrich), the cells were incubated with SE(Trojan-TLR7/8a) for 24 h. The cells were washed with PBS twice and then fixed with 4% paraformaldehyde at 4 °C for 15 min. Following another round of PBS washing, wheat germ agglutinin Texas red (Thermo Fisher, 5 µg ml^{-1}) was added for cell membrane staining, and the cells were incubated at room temperature (RT) for 15 min. Following the staining of the cell membrane, Hoechst 33342 (Invitrogen, 1 µg ml^{-1}) was also added for nuclei staining at RT for 15 min. Cell imaging was accomplished using a confocal laser scanning microscopy (CLSM) with a Leica TSC SP8 equipped with a 100x objective (Leica Microsystems). The filter sets (excitation (nm)/emission (nm)) used were as follows: Cy5 (646/664), TRITC (544/570), and DAPI (358/463).

To measure the secretion of cytokine IL-12(p70), BMDCs (1×10^6 cells per well) were seeded in 6-well culture plates. Supernatants were collected and then analysed using OptEIA ELISA kits (BD Biosciences) as per manufacturer's instructions. The absorbance was measured at 450 nm with a microplate reader (VersaMax).

In vitro BMDCs and BMDMs activation and cytokine secretion. BMDCs and BMDMs (1×10^6 cells per well) were seeded and then treated with SE, SE+R848 (1 µg ml^{-1}, 3.18 µM) or SE(Trojan-TLR7/8a) (2.9 µg ml^{-1}, 3.18 µM), each mixed with OVA (10 µg ml^{-1}). The supernatants were collected after 24 h of incubation and concentrations of pro-inflammatory cytokine (IL-12(p70), IL-6, IL-10, and TNF-α) were analysed using OptEIA ELISA kits (BD

Biosciences). The absorbance was measured at 450 nm with a microplate reader (VersaMax). For analysis of BMDCs and BMDMs activation, BMDCs were stained with BV421 anti-mouse-CD11c (clone: N418), APC anti-mouse CD40 (clone: 3/23), and FITC anti-mouse CD80 (clone: 16-10A1) (BioLegend). BMDMs were stained with APC anti-mouse/human CD11b (clone: M1/70), BV510 anti-mouse F4/80 (clone: BM8), FITC anti-mouse CD80 (clone: 16-10A1), and PE anti-mouse CD206 (clone: C068C2) (BioLegend). The expression of activation markers was analysed using BD FACSCanto™ II (BD Biosciences).

In vitro 12 h-maturated BMDC activation. BMDCs (1×10^6 cells per well) were seeded and then treated with SE+R848 (1 μg ml^{-1}, 3.18 μM) or SE(Trojan-TLR7/8a) (2.9 μg ml^{-1}, 3.18 μM), each mixed with OVA (10 ug ml^{-1}). After 12 h incubation, the cells were washed once with fresh medium and then refed with fresh medium. The cell supernatants were collected at 1, 4, 6, 8, and 12 h and IL-12(p70) concentrations were analysed using OptEIA ELISA kits (BD Biosciences).

Histological analysis. C57BL/6 female mice (6 weeks old) were immunized intramuscularly with SE(Trojan-TLR7/8a) (25 μg, 79.5nmol) mixed with OVA (20 μg) in the hind leg. The injected thigh muscles were harvested on day 1 post-immunization. The samples were sectioned and stained with Harris Hematoxylin solution (Sigma-Aldrich) and Eosin Y solution (Sigma-Aldrich). The stained sections were observed using an inverted microscopy (AE2000, Motic) with a 20x objective.

In vivo serum toxicity.

Necropsy, hematology, and serum biochemistry Male and female mouse (n = 5 per sex and group) were immunized intramuscularly with SE(Trojan-TLR7/8a) at concentrations of 159, 318, and 636 nmol, respectively. The four groups were divided into: high dose (0.2 mg/head), medium dose (0.1 mg/head), low dose (0.05 mg/head), and vehicle control (PBS in same volume). General clinic observations were observed continuously for the first 2 h after administration. Until the day of necropsy, all animals were checked daily for the observation of any clinical signs and weighed on day 1 (before administration), 2, 7, and 15 (day of necropsy). For necropsy analysis, all animals were first euthanized using CO_2 gas, followed by exsanguinations via the posterior vena cava and abdominal aorta. Various tissues, including liver, spleen, both kidneys, lung, thymus, and nerves gastrocnemius (administration site) were examined for the macroscopic morphology, and then removed quickly, washed in PBS, and

preserved in 10% neutral buffered formalin (Sigma-Aldrich). For hematology analysis, Blood samples were collected and placed into a bottle containing EDTA for the hematological analyses. Standard hematological parameters were analysed using an automatic blood analyzer (ADVIA 2120i, SIEMENS). The indicators were White blood cell (WBC), Red blood cell (RBC), Hemoglobin (HGB), Hematocrit (HCT), Mean corpuscular volume (MCV), Mean corpuscular hemoglobin (MCH), Mean corpuscular hemoglobin concentration (MCHC), Red cell distribution width (RDW), HGB distribution width (HDW), Platelet (PLT), Mean platelet volume (MPV), PLT distribution width (PDW), Neutrophil (NEU), Lymphocyte (LYM), Monocyte (MONO), Basophils (BASO), Eosinophil (EOS), Large unstained cell (LUC), and Reticulocyte (Reti). For serum biochemistry analysis, blood samples were collected and placed in a serum separation tube (SST), centrifuged at 488g for 10 min at 4 °C, and stored at −20 °C. At the end of the drug administration period, the biochemical blood index of mouse in each group was analysed using. The biochemical parameters were Aspartate transaminase (AST), Total cholesterol (TCHO), Calcium (Ca^{2+}), Alanine transaminase (ALT), Triglyceride (TG), Sodium (Na^{+}), Alkaline phosphatase (ALP), Total protein (TPRO), Potassium (K^{+}), Blood urea nitrogen (BUN), Albumin (ALB), Chloride (Cl^{-}), Total bilirubin (TBIL), Creatinine (CREA), Inorganic phosphorus (IP), Glucose (GLU), Creatine kinase (CK), and Globulin (GLOB).

Preparation of single-cell suspensions. Inguinal lymph nodes (iLNs) were mechanically disrupted and resuspended in a medium containing Collagenase D (1 mg ml^{-1}, Sigma-Aldrich). The solutions were incubated in a shaking incubator for 40 min at 37 °C. Subsequently, cells were filtered through 70 µm cell strainers and washed twice with PBS. Spleens were mechanically homogenized with a glass rod and resuspended in RBC lysis buffer (BioLegend) to remove RBCs. These solutions were filtered through 70 µm cell strainers and medium was added. Splenocytes were obtained after centrifuging the suspensions at 488g for 3 min. For cell extraction from muscles or lungs, tissues were resuspended in a medium containing collagenase D (1 mg ml^{-1}, Sigma-Aldrich). Then, tissues were mechanically homogenized with a glass rod and filtered through 70 µm cell strainers to prepare single-cell suspensions.

In vivo cell migration to iLNs. C57BL/6 female mice (6 weeks old) were immunized intramuscularly with SE, SE+R848 (25 µg, 79.5 nmol), or SE(Trojan-TLR7/8a) (72.1 µg, 79.5 nmol), each mixed with OVA (20 µg) or Alexa Fluor 647 (AF647)-labelled OVA (40 µg, Invitrogen) in the hind leg.

For flow cytometry analysis to determine the phenotype of dendritic cells (DCs), iLNs were excised at day 0.5, 1, 3, and 5. Single cells from iLNs were labelled with fixable viability dye eFluor™ 780 (1:2000, eBioscience). Cells were stained with BV421 anti-mouse CD11c (clone: N418), PE anti-mouse CD8a (clone: 53-6.7), APC anti-mouse CD103 (clone: 2E7), BV510 anti-mouse CD3 (clone: 17A2), and FITC anti-mouse CD80 (clone: 16-10A1) (BioLegend) antibodies.

For flow cytometry analysis of the number of immune cells, including macrophages, DCs, germinal center (GC) B cells, and follicular T cells (T_{FH}), iLNs were excised at day 0.5, 1, 3, 5, 7, and 14. Single cells from iLNs were labelled with fixable viability dye eFluor™ 780 (1:2000, eBioscience). Cells were stained with PerCP/Cy5.5 anti-mouse/human CD11b (clone: M1/70), BV510 anti-mouse CD11c (clone: N418), PE anti-mouse/human CD45R/B220 (clone: RA3-6B2), FITC anti-mouse Fas (CD95) (clone: SA367H8), BV421 anti-mouse CD38 (clone: 90), BV421 anti-mouse CXCR5 (clone: L138D7), PE anti-mouse PD-1 (clone: 29F.1A12), BV510 anti-mouse CD3 (clone: 17A2) (BioLegend), and FITC anti-mouse CD4 (clone: RM4-5) (BD Biosciences) antibodies.

For the analysis of OVA-specific $CD8^+$ T cells, iLNs were excised at 7 days and prepared into single cells. Single cells (5×10^5 cells per well) were plated in a round-bottom 96-well plate. Then, the cells were restimulated with the $OVA_{257-264}$ peptide (SIINFEKL) (10 μg ml^{-1}, MIMOTOPES), IL-2 (30 ng ml^{-1}, PeproTech), and GolgiPlug (protein transport inhibitor, 0.6 μg ml^{-1}, BD Biosciences) for 12 h. After stimulation, cells were collected and washed once. Cells were labelled with fixable viability dye eFluor™ 780 (1:2000, eBioscience) and stained with surface markers, including BV510 anti-mouse-CD3 (clone: 17A2), and PE anti-mouse-CD8a (clone: 53-6.7) (BioLegend) antibodies for 30 min at 4 °C. Then, cells were washed and permeabilized with fixation/permeabilization solution for 20 min at 4 °C. For intracellular staining, fixed/permeabilized cells were washed once with BD Perm/Wash buffer (BD Biosciences) and stained with BV421 anti-human/mouse Granzyme B (clone: QA18A28), APC anti-mouse TNF-α (clone: MP6-XT22), and FITC anti-mouse IFN-γ (clone: XMG1.2) (BioLegend) antibodies for 30 min at 4 °C. Flow cytometry data were analysed using BD FACSCanto™ II (BD Biosciences) and quantified using FlowJo v.10 software. The gating strategy used is provided in Supplementary Fig.15.

OVA specific IgG titer ELISA assay

C57BL/6 female mice (6 weeks old) were immunized intramuscularly with SE (with the same volume used for SE(Trojan-TLR7/8a)) or SE(Trojan-TLR7/8a) (72.1 μg, 79.5 nmol), each

mixed with OVA (20 μg) in the hind leg at week 0 and boosted at week 3. After 1 week following boosting immunization, blood samples were collected and centrifuged at 10,000g for 10 min to separate serum for ELISA assays to evaluate OVA-specific antibodies. For IgG, IgG1, and IgG2c ELISA, 96-well Immunoplates were coated with OVA (2 μg ml^{-1}) overnight at RT. The plates were then blocked with a 5% (w/v) skim milk solution for 2 h at 37 °C, washed with 0.05% (v/v) Tween-20, and loaded with serum samples for 2 h at 37 °C. The plates were incubated with secondary antibodies: goat anti-mouse IgG(H+L)-HRP, rat anti-mouse IgG1-HRP, or goat anti-mouse IgG2c-HRP (1:6,000, Southern Biotech). After incubation for 1 h, detection was performed with TMB substrate solution. The absorbance was measured at 450 nm using a microplate reader (VersaMax).

Lymph node fluorescence imaging. C57BL/6 female mice (6 weeks old) were immunized intramuscularly with DiD (Invitrogen) in the soluble form, DiD-loaded SE, DiD-loaded SE+R848 or DiD-loaded SE(Trojan-TLR7/8a), each mixed with FITC-labelled OVA (40 μg, Invitrogen) in the hind leg. For immunofluorescence of iLNs, the iLNs were excised at 0.5, 1, 3, 5, and 7 days after immunization. iLN imaging was accomplished with an in vivo imaging system Lumina XR (PerkinElmer). Signals of DiD were measured at an excitation wavelength of 640 nm and an emission wavelength of 690 nm and signals of FITC were measured at an excitation wavelength of 465 nm and an emission wavelength of 535 nm.

Comparison with commercialized vaccine adjuvants. C57BL/6 female mice (6 weeks old) were immunized intramuscularly with Alum (200 μg, Alhydrogel 2%, InvivoGen), AS03 (with the same volume used for SE(Trojan-TLR7/8a), AddaS03™, InvivoGen), SE (with the same volume used for SE(Trojan-TLR7/8a)), or SE(Trojan-TLR7/8a) (72.1 μg, 79.5 nmol), each mixed with OVA (20 μg), or LNP(mRNA) (5 μg) in the hind leg at week 0 and boosted at week 3.

Analysis of long-lived plasma cells (LLPCs). C57BL/6 female mice (6 weeks old) were immunized intramuscularly with SE or SE(Trojan-TLR7/8a) (72.1 μg, 79.5 nmol), each mixed with OVA (20 μg) in the hind leg at week 0 and boosted at week 3. For analysis of LLPCs, cells from bone marrow were harvested at week 6. Cells were labelled with fixable viability dye eFluor™ 780 (1:2000, eBioscience) and stained with APC anti-mouse CD138 (clone: 281-2), and FITC anti-mouse/human CD45R/B220 (clone: RA3-6B2) (BioLegend) antibodies. Flow

cytometry data were using BD FACSCanto™ II (BD Biosciences) and quantified using FlowJo v.10 software.

Whole-mouse fluorescence imaging. To evaluate the retention time of SE(Trojan-TLR7/8a) at injection sites and antigen delivery capability, C57BL/6 female mice (6 weeks old) were immunized intramuscularly with DiD (Invitrogen) in the soluble form, DiD-loaded SE, DiD-loaded SE+R848 or DiD-loaded SE(Trojan-TLR7/8a), each mixed with Alexa Fluor 647 (AF647)-labelled OVA (40 μg, Invitrogen) in the hind leg. Signals of DiD or AF647 were measured with a whole-animal in vivo imaging system Lumina XR (PerkinElmer). Images were acquired using a 640nm emission filter from 6 h to 7 days post-immunization.

mRNA-Seq. C57BL/6 female mice (6 weeks old) were immunized intramuscularly with SE or SE(Trojan-TLR7/8a) (72.1 μg, 79.5 nmol), each mixed with OVA (20 μg) in the hind leg at week 0 and boosted at week 3. For sequencing, iLNs were excised on day 7 after boosting immunization.

RNA isolation

Total RNA was isolated from iLNs using Trizol reagent (Invitrogen). RNA quality was evaluated using TapeStation4000 System (Agilent Technologies), and quantification of RNA was performed using ND-2000 Spectrophotometer (Thermo Fisher).

Library preparation and sequencing

Libraries for sequencing were prepared from total RNA using the CORALL RNA-Seq V2 Library Prep Kit (LEXOGEN). The Poly(A) RNA Selection Kit (LEXOGEN) was used to isolate mRNA, and cDNA was prepared from the isolated mRNA according to the manufacturer's instructions. Differential gene analysis was carried out based on NovaSeq6000 (Illumina). Data mining and graphic visualization were performed using ExDEGA (Ebiogen).

Production of SARS-CoV-2 spike pseudovirus. Plasmids encoding the SARS-CoV-2 Spike protein (Wuhan-Hu-1 strain) were obtained from Sino Biological (pCMV3-SARS-CoV-2 Spike, VG40589-UT). Mutants of the SARS-CoV-2 Spike protein were generated through site-directed mutagenesis or by synthesizing mutant SARS-CoV-2 Spike DNA. SARS-CoV-2 pseudoviruses were produced by co-transfecting 293T cells with pMDLg/pRRE (Addgene plasmid, 12251), pRSV-Rev (Addgene plasmid, 12253), pCDH-CMV-Nluc-copGFP-Puro (Addgene plasmid, 73037), and plasmids encoding the SARS-CoV-2 Spike, using polyetherimide as the transfection agent. After transfection for 16 h, the culture supernatants

containing the SARS-CoV-2 Spike pseudoviruses were harvested and filtered through a 0.45 μm pore-size filter (S2HVU01RE, Millipore). The copy number of pseudoviruses in these supernatants was subsequently quantitated by quantitative PCR (qPCR) using reagents from Takara Bio, USA.

SARS-CoV-2 viruses and antigen preparation. SARS-CoV-2 spike stabilized trimers from B.1.1.529 (Omicron variant, product #REC32008) were purchased from the Native Antigen Company. SARS-CoV-2 variants (Wuhan strain; hCoV-19/South Korea/NMC-02/2020, Alpha (B.1.1.7); hCoV-19/South Korea/NMC-nCoV-07/2021, Beta (B.1.351); hCoV-19/South Korea/NMC-nCoV-08/2021, Delta (B.1.617.2); hCoV-19/South Korea/NMC-nCoV-11/2021, BA.2/omicron (B.1.1.529); hCoV-19/South Korea/CBNU-nCoV-55/2021) and mouse-adapted SARS-CoV-2 (originated from Wuhan strain) were used for serum neutralization assay and viral challenge experiment, respectively. The viruses were propagated in Vero-E6 monolayers using DMEM (Gibco) for 1 h at 37 °C in 5% CO_2. After incubation, the medium was changed to 2% FBS-supplemented DMEM. After infection for 3 days, viruses were harvested from the infected Vero-E6 cells, centrifuged at 1,000g for 20 min, and then stored at -80 °C until use. All experiments were performed under biosafety level 3 conditions. For inactivation of virion, SARS-CoV-2 in culture supernatant was treated with β-propiolactone for 72 h at 4 °C. The inactivated virions were purified using ultracentrifugation at 100,000g for 2 h in a 30% sucrose density gradient. BCA Protein Assay Kits (Thermo Fisher) were used for the quantification of purified virions.

Flow cytometry analysis for SARS-CoV-2 antigen model in spleens. For antigen-specific polyfunctional $CD8^+$ T cells, single cells were restimulated with S peptide pool (1 μg ml^{-1}, Sino Biological) or inactivated SARS-CoV-2 variants (2 μg ml^{-1}) in the presence of monensin for 12 h. After stimulation, cells were collected and washed once. Cells were stained with surface markers, including FITC anti-mouse CD8a (clone: 53-6.7), and PerCP/Cy5.5 anti-moue CD3 (clone: 145-2C11) (BD Biosciences) antibodies for 30 min at 4 °C. Then, cells were washed and permeabilized with fixation/permeabilization solution for 20 min at 4 °C. For intracellular staining, fixed/permeabilized cells were washed once with eBioscience™ Foxp3 / Transcription Factor Staining Buffer Set (Thermo Fisher) and stained with PE/Cy7 anti-mouse IL-2 (clone: JES6-5H4), APC anti-mouse TNF-α (clone: MP6-XT22), and PE anti-mouse IFN-γ (clone: XMG1.2) (BD Biosciences) antibodies for 30 min at 4 °C. To test $CD4^+$ T_{FH} cells, single cells were restimulated with S peptide pool (1 μg ml^{-1}, Sino Biological) or inactivated SARS-CoV-

2 variants (2 μg ml^{-1}) in the presence of monensin for 12 h. After stimulation, cells were collected and washed once. Cells were stained with surface markers, including APC/Cy7 anti-mouse CD4 (clone: GK1.5), PerCP/Cy5.5 anti-moue CD3 (clone: 145-2C11), and BV421 anti-mouse CD279 (PD-1) (clone: RMP1-30) (BD Biosciences) antibodies for 30 min at 4 °C. Then, cells were washed and permeabilized with fixation/permeabilization solution for 20 min at 4 °C. For intracellular staining, fixed/permeabilized cells were washed once with eBioscience™ Foxp3 / Transcription Factor Staining Buffer Set (Thermo Fisher) and stained with PE/Cy5 anti-mouse IL-21 (clone: 4A9) (BioLegend) antibodies for 30 min at 4 °C. To observe GC B cells, single cells were stained with BV711 anti-mouse CD19 (clone: 6D5, BioLegend) and Alexa Fluor 488 anti-human/mouse GL7 (clone: GL7, Invitrogen) antibodies for 30 min at 4 °C. Intracellular staining was performed using anti-activation-induced cytidine deaminase (AID)-biotin Ab (clone: mAID-2, Invitrogen) and streptavidin-PE (Invitrogen). Flow cytometry data were analysed using a FACSymphony™ A3 (BD Biosciences) and analysed using FlowJo v.10 software.

T and B cell immune responses for SARS-CoV-2 antigen model. For measuring the T cell immune response, the frequency of IFN-γ-producing T cells was evaluated using mouse IFN-γ ELISPOT kits (BD Biosciences). Briefly, the splenocytes were plated at 5×10^5 cells per well onto purified IFN-γ Ab-coated ELISPOT plates and stimulated with 1 μg ml^{-1} of S peptide pool (Sino Biological) and 1 μg ml^{-1} of BPL-inactivated SARS-CoV-2 variants for 60 h at 37 °C. The SFUs were enumerated using an ELISPOT plate reader (iSpot SPECTRUM System, AID Diagnostika). For measurement of the levels of antigen-specific B cell responses, ELISA plates were coated with 0.5 μg ml^{-1} SARS-CoV-2 spike stabilized trimers, washed with PBS-T (PBS containing 0.05% Tween 20), and blocked with 5% skim milk in PBS-T. The sera from each group were reacted to the antigen-coated plates followed by incubation with the anti-mouse IgG-HRP (Abcam). The plates were washed and developed with the chromogenic tetramethylbenzidine substrate (Merck), and the reactions were terminated with 2 N H_2SO_4. The absorbance was measured at 450 nm using an Infinite M plex microplate reader (Tecan). To examine neutralizing antibodies in serum for immunized mice, a neutralizing antibody (NAb) assay against SARS-CoV-2 variants was carried out using a serum-neutralization assay in Vero-E6 cells. Collected serum were inactivated for 30 min at 56 °C. Initial 1:10 serum dilutions were prepared using serum-free RPMI1640 medium (Hyclone), and two-fold serial dilutions of all samples were made to achieve final serum dilution ranging from 1:10 to 1:12800. For each well, serially diluted serum was mixed with 100 $TCID_{50}$ of SARS-CoV-2 and

incubated at 37 °C for 1 h to neutralize the infectious virus. The mixtures were transferred to the Vero-E6 cell monolayers, followed by incubation for 1 h at 37 °C in 5% CO_2, and then changed to 2% FBS-supplemented DMEM medium. After 3 days, neutralization of the infectious virus was monitored for in cytopathic effect (CPE).

ELISA for antibodies specific to sM2, HA2 and sM2HA2. 96-well plate immunosorbent plates (Coatar) were coated with 200 ng per well of sM2, HA2, and sM2HA2 in a coating buffer (pH 9.6) and incubated overnight at 4 ºC for serum IgG, IgG1 and IgG2a ELISA. After washing with PBS-T, the plates were blocked with 200 µl of 10% skim milk at room temperature for 2 h and serial twofold dilution (1:50 to 1:1600) of serum samples were added to the wells and incubated further for 2 h at 37 ºC, followed by treatment of 1:3000 diluted goat anti-mouse IgG (GeneTex), IgG1 and IgG2a-HRP (Invitrogen) at 37 ºC for 2h. Then, 100 µl of tetramethylbenzidine and H_2O_2-containing substrate solution (BD Biosciences) was added for 10 min in the dark, and the reaction was stopped by adding 2N-H_2SO_4. Finally, the OD was measured at 450 nm using a scanning multi-well spectrophotometer (ELISA reader, Molecular Devices).

ELISPOT assay for specific to sM2, HA2 and sM2HA2. IFN- γ and IL-4 ELISPOT assays were performed on splenocytes using mouse IFN- γ and IL-4 ELISPOT kits (BD Biosciences). Briefly, BD science 96-well plates were coated with anti-mouse IFN-γ, or IL-4 capture antibodies (5 µg ml^{-1}) in PBS and incubated at 4 ºC for overnight. After discarding the antibodies, plates were blocked with complete RPMI1640 medium (Hyclone) containing 10% FBS (Gibco) and incubated for 2 h at room temperature. Subsequently aseptically isolated splenocytes from immunized mice were added at 1×10^6 cells per well in media containing 1 µg per well of M2 or HA2 peptide or sM2HA2 purified protein or medium only (negative control) or 0.5 µg per well of phytohemagglutinin (Positive control, Invitrogen). Then plates were incubated for 24 h for IFN-γ and 48 h for IL-4 at 37 ºC in 5% CO_2, followed by sequentially adding of biotinylated anti-mouse IFN-γ and IL-4 antibodies, streptavidin-HRP and substrate solution (BD Biosciences). Finally, plates were washed with deionized water after visualizing the IFN-γ and IL-4 secreting T cells, and plates were dried in a dark condition before counting the spots automatically using an Immuno Scan Entry analyzer (Cellular Technology Ltd., Shaker Heights). Peptide sequence information was mentioned in Supplementary Table 6.

Lung influenza viral titers analysis. The virus titer was determined by 50% tissue culture infective dose ($TCID_{50}$). Immunized mice were challenged with H1N1 and H5N2 and sacrificed at 3 and 5 days after infection to harvest the lungs. The harvested lungs were homogenized in PBS containing antibiotic and antimycotic agents (Hyclone), and the cell debris was removed by centrifugation at 12,000g. Ten-fold serially diluted samples were prepared and incubated with confluent Madin–Darby canine kidney (MDCK) cells at 37°C for 1 h. Subsequently, cells were incubated with an overlay medium containing L-1-tosylamide-2-phenylethyl chloromethyl ketone (TPCK) trypsin (Sigma-Aldrich) for 72 h. The hemagglutinin (HA) test was performed after observing the cytopathic effect (CPE), and the virus titer was calculated by the Reed and Muench method and expressed as $\log_{10}$ $TCID_{50}$/lung tissue.

Preparation of SFTSV and cells. For viral propagation, the SFTSV (CB1/2014) was propagated in Vero-E6 cell monolayers (ATCC no. CRL-1586; American Type Culture Collection) using DMEM (Gibco) for 1 h at 37 °C in 5% CO_2. After incubation, the medium was changed to 2% FBS-supplemented DMEM. After infection for 5 days, viruses were harvested from the infected Vero-E6 cells, and then stored at −80 °C to serve as the working virus stocks for this study. Viral infectivity titers were determined by an immunostaining assay, with the $TCID_{50}$ calculated using an in-house-generated monoclonal NP antibody against SFTSV in an immunofluorescence assay.

Serum-neutralization assay. To evaluate neutralizing antibodies in serum for immunized ferrets, NAb assay against SFTSV was carried out using a serum-neutralization assay in Vero-E6 cells. Collected serum were inactivated for 30 min at 56 °C. Initial 1:10 serum dilutions were prepared using serum-free DMEM medium, and two-fold serial dilutions of all samples were made to achieve final serum dilution ranging from 1:10 to 1:2560. For each well, serially diluted serum was mixed with 100 $TCID_{50}$ of SFTSV (CB1/2014) and incubated at 37 °C for 1 h to neutralize the infectious virus. The mixtures were transferred to the Vero-E6 cell monolayers, followed by incubation for 1 h at 37 °C in 5% CO_2, and then changed to 2% FBS-supplemented DMEM medium. After 5 days, neutralization of the infectious virus was confirmed through immunofluorescence staining using SFTSV NP antibody.

Quantification of viral copy numbers by quantitative reverse transcription (qRT-PCR). RNA extraction was carried out using TRIzol reagent (Thermo Fisher) or the RNeasy kit (Qiagen). Subsequently, cDNA synthesis was performed using SuperiorScript III Reverse

Transcriptase (Enzynomics) with a specific primer set designed to target the M segment of SFTSV. The forward primer used was SFTSV-M-F: AATTCACATTTGAGGGTAGTT, and the reverse primer used was SFTSV-M-R: TATCCAAGGAGGATGACAATAAT. Real-time PCR was performed using SYBR Green Supermix (Bio-Rad) and the CFX96 real-time PCR detection system (Bio-Rad). The copy numbers determined through experimentation were calculated as a ratio based on the standard control (PMID: 25817401).

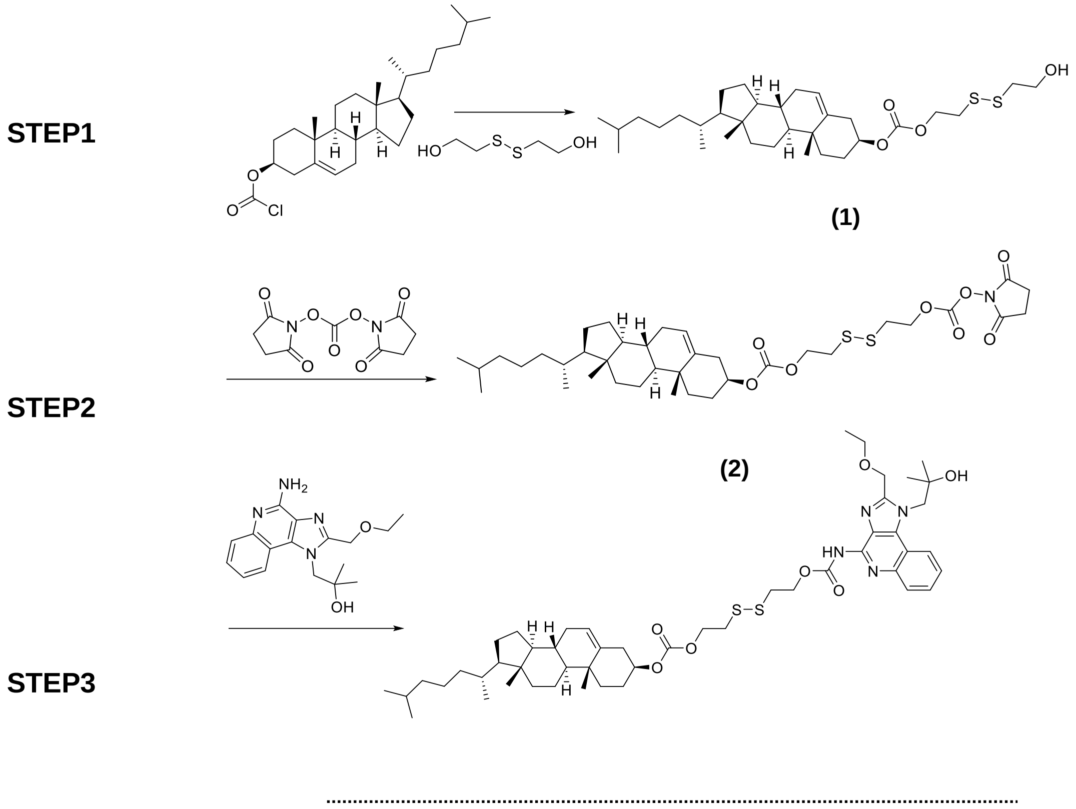

Trojan-TLR7/8a

Supplementary Figure 1. Synthesis and characterization of Trojan-TLR7/8a.

STEP1: Cholesteryl chloroformate (40 g) was reacted with bis(2-hydroxyethyl) disulfide in DCM (100 ml) and pyridine at 10-15 °C, then stirred at room temperature (RT) for 2 h. Water was added to terminate the reaction, and the organic phase was extracted with DCM, washed with brine, and dried over Na_2SO_4. The product was purified by gel chromatography to give a yellow gel with a 39.6% yield.

STEP2: The intermediate (1) (20 g) was dissolved in DCM (200 ml), reacted with bis(2,5-dioxopyrrolidin-1-yl) carbonate and TEA (10.7 g), and stirred at RT for 3 hours. Post-reaction, the mixture was washed and dried similar to the previous step and purified using silica gel column chromatography, eluting with 5-20% ethyl acetate (EtOAc) in n-hexane. This produced a yellow gel with a 72% yield.

STEP3: R848 was dissolved in DCM and reacted with compound (2) and TEA at 10-20 °C. The reaction was stirred overnight at 20-25 °C. Following the reaction, the process of extraction, washing, and drying was repeated, and the product was purified using silica gel column chromatography with 10-50% EtOAc in n-hexane. The final product, Trojan-TLR7/8a was a white solid with a 37.4% yield.

^{1}H NMR (Bruker Avance III 700 MHz, $CDCl_3$): δ 8.15-8.17 (m, 2H), 7.60-7.64 (m, 1H)), 7.47-7.51 (m, 1H), 5.39-5.40 (m, 1H), 4.93 (s, 2H), 4.81 (s, 2H), 4.56 (t, *J* = 6.4 Hz, 2H), 4.45-4.54 (m, 1H), 4.41 (t, *J* = 6.4 Hz, 2H), 3.68 (q, *J* = 6.8 Hz, 2H), 3.13 (s, 1H), 3.09 (t, *41 (t, 4 Hz* = 6.4 Hz, 2H), 3.68 (q, *J* = 6.8 Hz, 2H), 2.34-2.47 (m, 2H), 1.92-2.06 (m, 3H), 1.79-1.90 (m, 2H), 1.23-1.72 (m, 21H), 1.06-1.21 (m, 7H), 0.96-1.05 (m, 5H), 0.93 (d, *J* = 6.4 Hz, 3H), 0.88 (dd, *J* = 1.6, 6.4 Hz, 6H), and 0.69 (s, 3H).

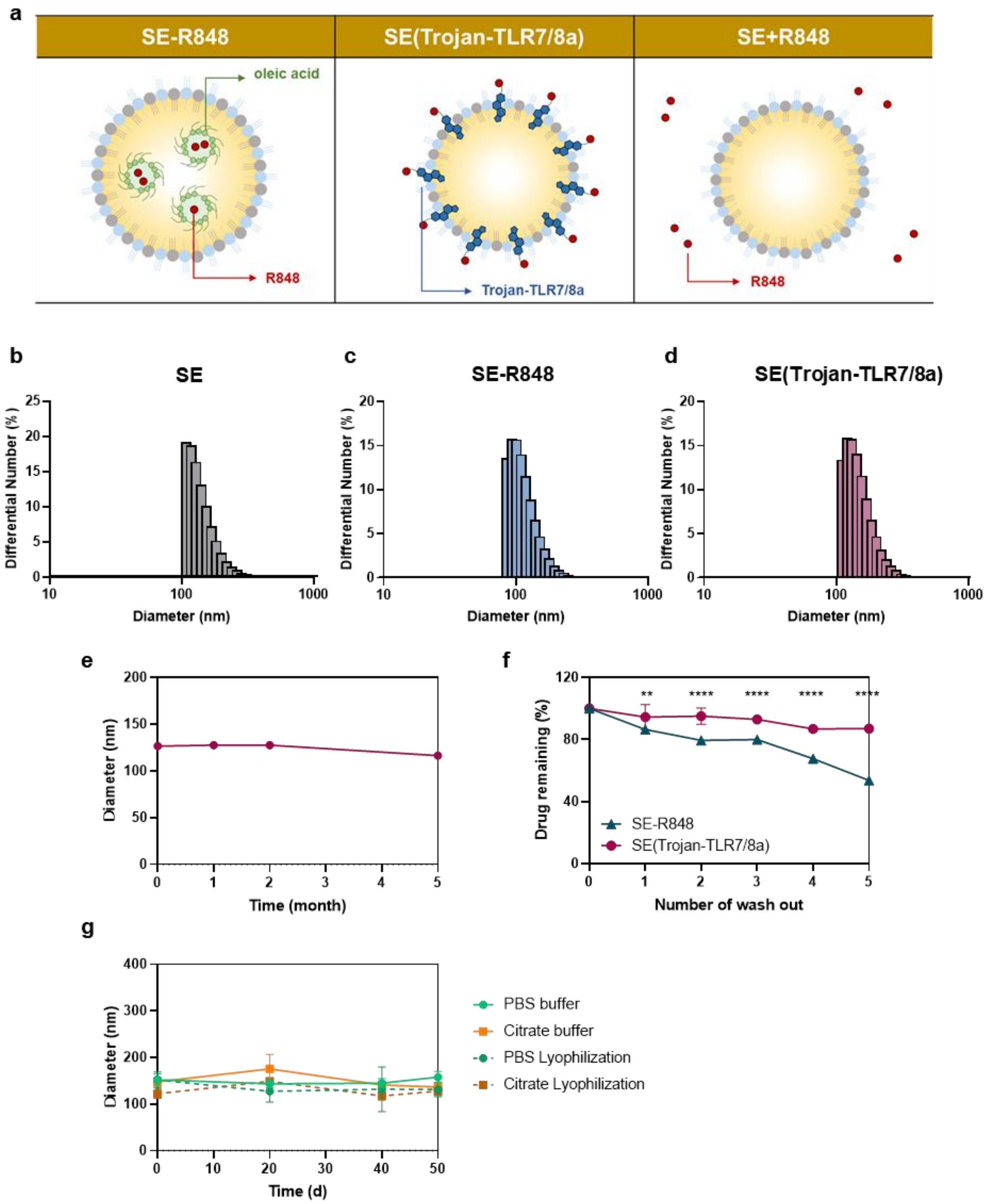

Supplementary Figure 2. Characterization of squalene-based nanoemulsion. a, Composition of SE-based adjuvants. **b-d,** Representative size distribution of SE (**b**), SE-R848 (**c**), and SE(Trojan-TLR7/8a) (**d**). **e,** Average size of SE(Trojan-TLR7/8a) in 4 °C for long period. **f,** Percentage of the cumulative remaining drug after number of wash out (n = 4). **g,** Average size of SE(Trojan-TLR7/8a) based on buffer type (PBS or citrate buffer) and the presence of lyophilization (n = 4). The data are presented as mean ± standard deviation (s.d). In **f,** analysis was performed by two-way ANOVA with Tukey's multiple comparison test. P values are indicated (n.s., not significant; * $P < 0.05$, ** $P < 0.01$, *** $P < 0.001$, **** $P < 0.0001$).

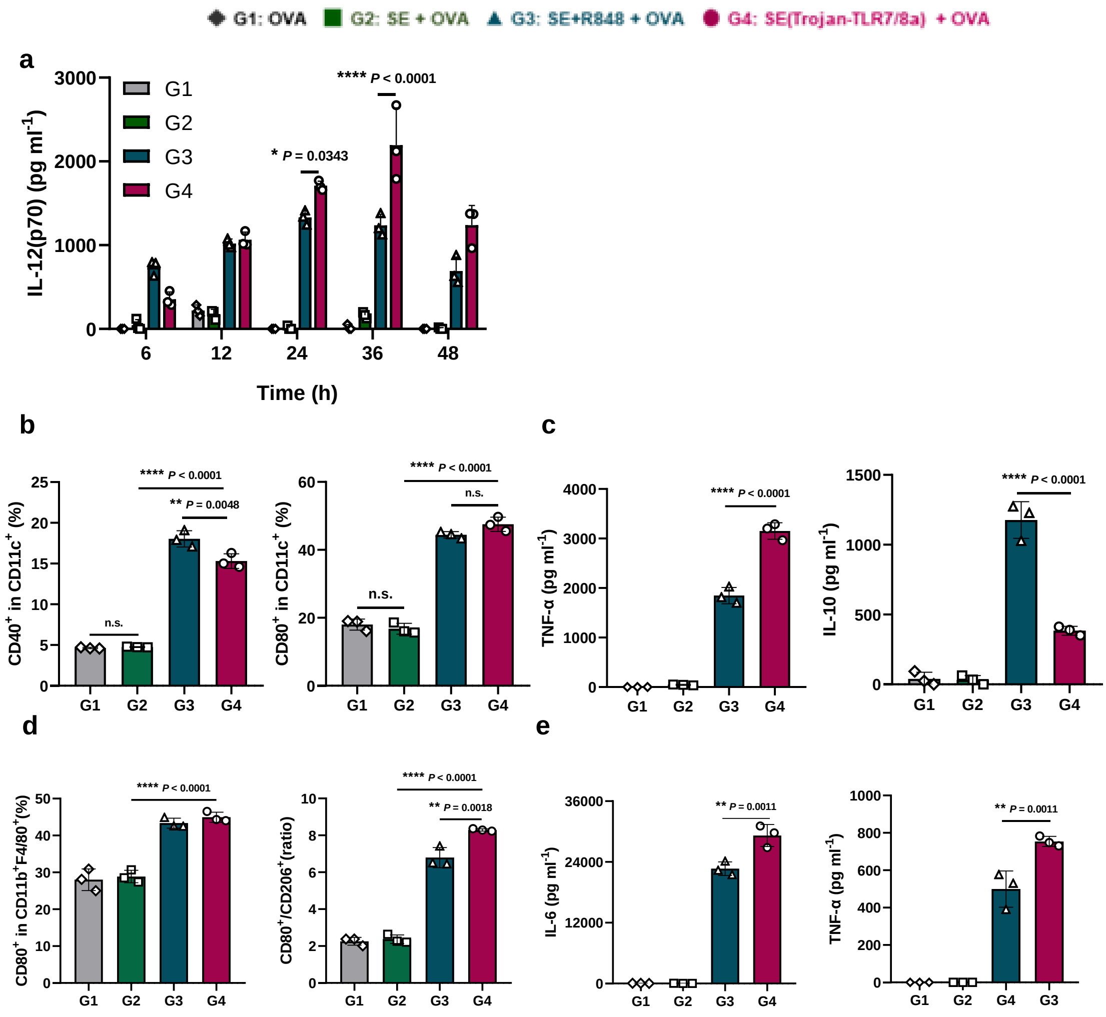

Supplementary Figure 3. *In vitro* analysis of activation markers and pro-inflammatory cytokines of BMDCs or BMDMs. a-e, BMDCs (**a-c**) or BMDMs (**d-e**) were treated with SE, SE+R848 (1 μg ml^{-1}, 3.18μM), or SE(Trojan-TLR7/8a) (2.9 μg ml^{-1}, 3.18 μM), each mixed with OVA (10 μg ml^{-1}) for 24 h. **a,** The cell culture supernatants were collected at 6, 12, 24, 36, and 48 h and the kinetics of IL-12(p70) secretion were measured by ELISA (n = 3). **b,** Percentage of CD40$^+$ and CD80$^+$ in CD11c$^+$ BMDCs were analysed using flow cytometry (n = 3). **c,** The cell culture supernatant was collected 24 h after treatment, and concentration of TNF-α and IL-10 were measured by ELISA (n = 3). **d,** Percentage of CD80$^+$ and ratio of CD80$^+$ (M1)/CD206$^+$(M2) in CD11b$^+$ F4/80$^+$ cells were analysed using flow cytometry (n = 3). **e,** The cell culture supernatant was collected 24 h after treatment, and concentration of IL-6, and TNF-α was measured by ELISA (n = 3). Data are presented as mean ± s.d. In **a,** analysis was performed by two-way ANOVA with Tukey's multiple comparison test. In **b-e,** analysis was performed by one-way ANOVA with Tukey's multiple comparison test. P values are as indicated (n.s. not significant, * P < 0.05, ** P < 0.01, *** P < 0.001, **** P < 0.0001).

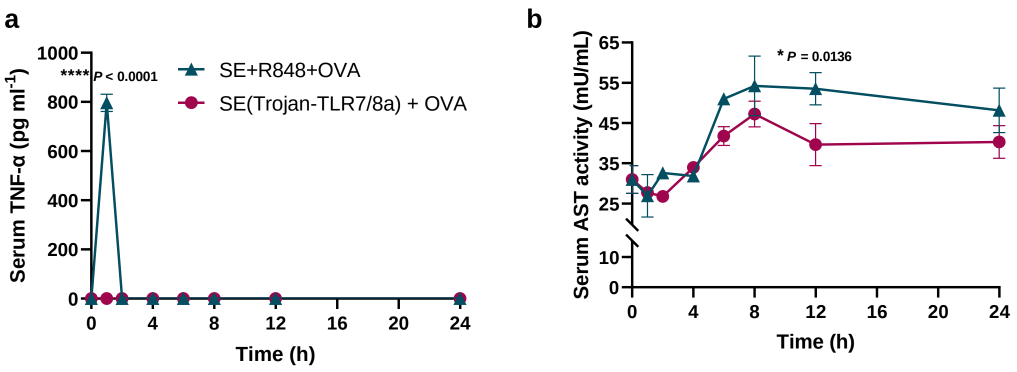

Supplementary Figure 4. Serum IL-6 and AST activity test after intramuscular injection of SE+R848 or SE(Trojan-TLR7/8a). a,b, C57BL/6 mice were immunized intramuscularly with SE+R848 (25 µg, 79.5 nmol), or SE(Trojan-TLR7/8a) (72.1 µg, 79.5 nmol), each mixed with OVA (20 µg). Blood was collected at 1, 2, 4, 6, 8, 12, and 24 h following immunization. Serum was obtained after centrifugation at 10,000g for 10 min at 4 °C. **a,** Concentration of TNF-α (n = 2 mice per group) in serum were quantified by ELISA. **b,** Serum AST activity (n = 2 mice per group). The data are presented as mean ± s.d. In **a,** and **b,** analysis was performed by two-way ANOVA with Tukey's multiple comparison test. P values are indicated (n.s., not significant; * $P < 0.05$, ** $P < 0.01$, *** $P < 0.001$, **** $P < 0.0001$).

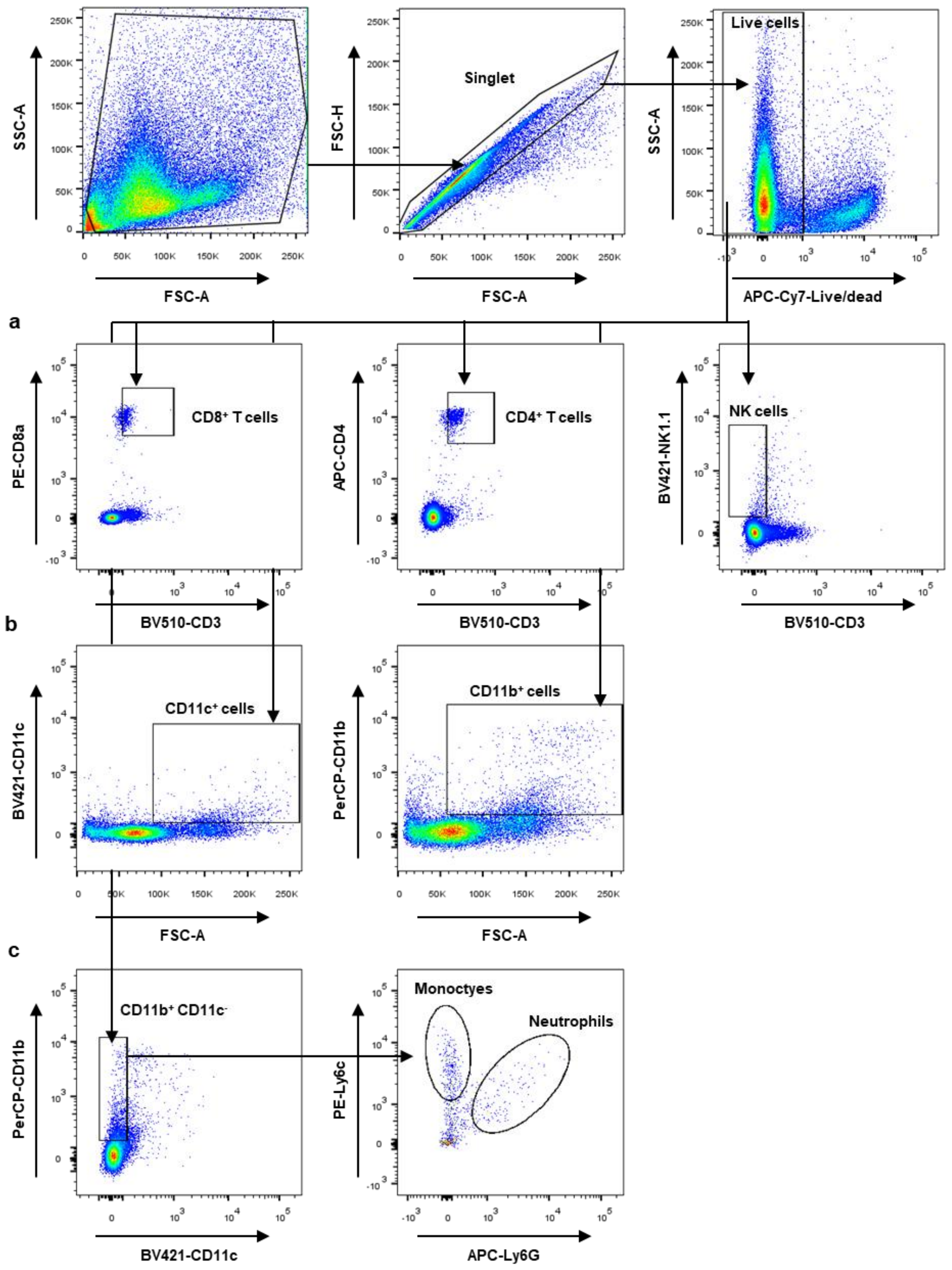

Supplementary Figure 5. Flow cytometry gating strategy for the analysis of immune cells *in vivo* in muscle tissues. **a,** Populations of $CD8^+$ T cells ($CD3^+$ $CD8^+$), $CD4^+$ T cells ($CD3^+$ $CD4^+$), and NK cells ($CD3^–$ $NK1.1^+$). **b,** Populations of $CD11c^+$ and $CD11b^+$ cells. **c,** Populations of neutrophils ($Ly6G^+$ $Ly6C^–$) and monocytes ($Ly6G^–$ $Ly6C^+$) in $CD11c^+$ $CD11b^–$ cells. The gating strategy for all samples was established on single cells after excluding large clumps, cell aggregates, and cell debris (FSC-H and FSC-A gating), and dead cells (live/dead gating).

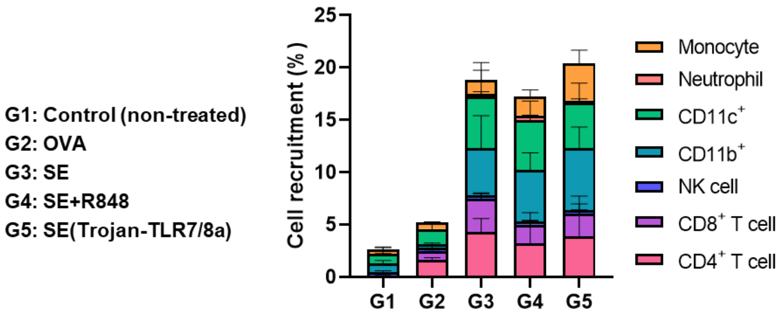

Supplementary Figure 6. Immune cell infiltration to injection site (muscle tissue). C57BL/6 mice were immunized intramuscularly with SE, SE+R848 (25 µg, 79.5 nmol), or SE(Trojan-TLR7/8a) (72.1 µg, 79.5 nmol), each mixed with OVA (20 µg). Muscle tissues were excised at day 1 (*n* = 3 mice per group). Proportions of monocytes (Ly6G$^-$ Ly6C$^+$), neutrophils (Ly6G$^+$ Ly6C$^-$), CD11c$^+$, CD11b$^+$, NK cells (CD3$^-$ NK1.1$^+$), CD8$^+$ T cells (CD3$^+$ CD8$^+$), and CD4$^+$ T cells (CD3$^+$ CD4$^+$) recruited into the muscle at 24 h (*n* = 3 mice per group). The data are presented as mean ± s.d. Analysis was performed by two-way ANOVA with Tukey's multiple comparison test. *P* values are indicated (n.s., not significant; * $P < 0.05$, ** $P < 0.01$, *** $P < 0.001$, **** $P < 0.0001$).

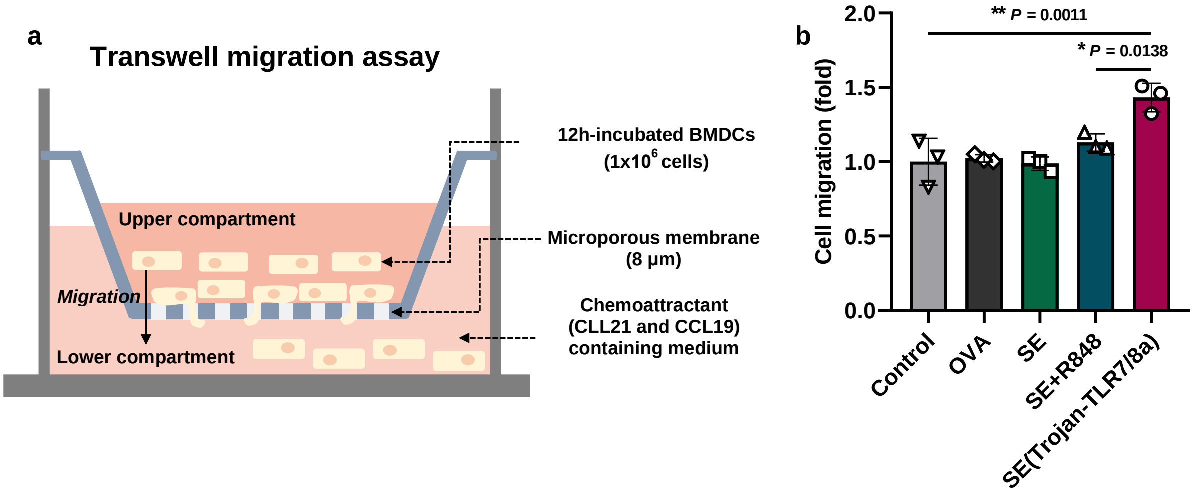

Supplementary Figure 7. DC migration induced by SE(Trojan-TLR7/8a) under chemoattractant. BMDCs were treated with SE, SE+R848 (1 µg ml^{-1}, 3.18 µM), or SE(Trojan-TLR7/8a) (2.9 µg ml^{-1}, 3.18 µM), each mixed with OVA (10 µg ml^{-1}) for 24 h. **a,** Schematic illustration of Transwell migration assay. **b,** The migratory ability of treated BMDCs was assessed through transwell migration assay. BMDCs were harvested 12 h after treatment and plated into Transwell inserts. Chemoattractants CCL21 (50 ng ml^{-1}) and CCL19 (50 ng ml^{-1}) were added to the bottom chamber. Following a 24 h incubation, the migratory ability of treated BMDCs was assessed in comparison to the control group through transwell migration assay (*n* = 3). The data are presented as mean ± s.d. In **b,** analysis was performed by one-way ANOVA with Tukey's

multiple comparison test. *P* values are indicated (n.s., not significant; * $P < 0.05$, ** $P < 0.01$, *** $P < 0.001$, **** $P < 0.0001$).

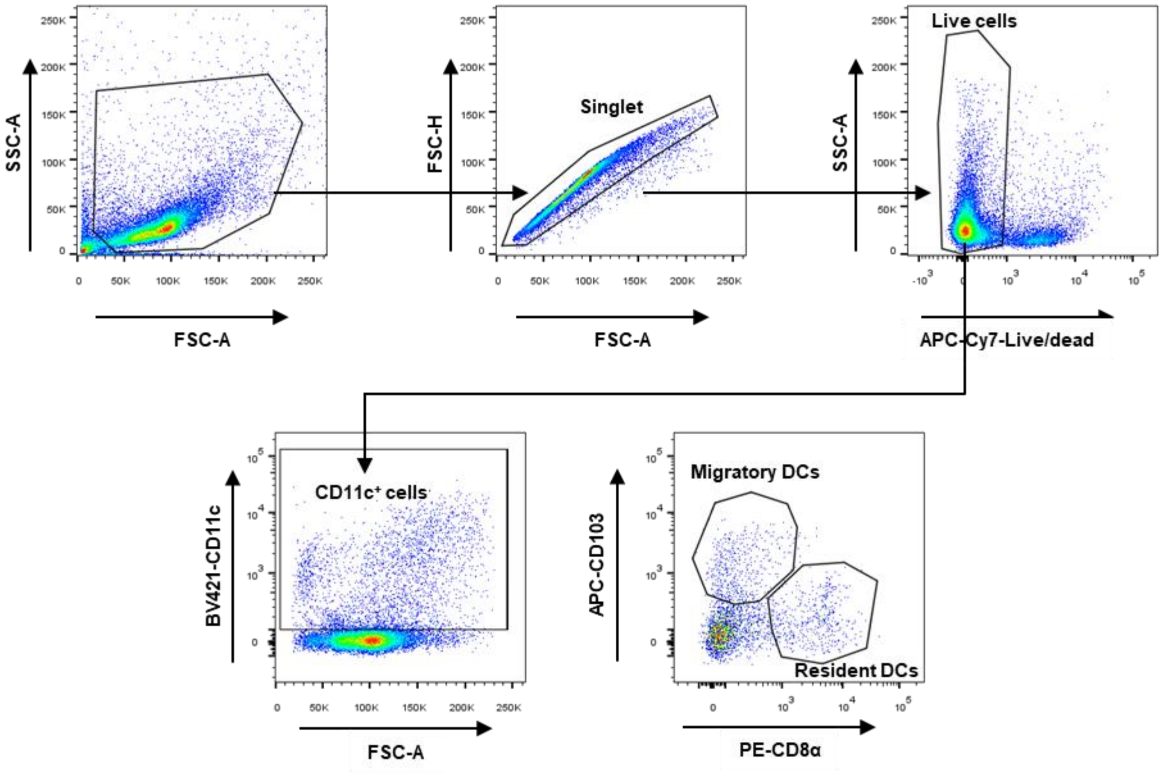

Supplementary Figure 8. Flow cytometry gating strategy for the analysis of immune cells *in vivo* in iLNs. Populations of migratory DCs (CD8α^{-} CD103^{+} in CD11c^{+}) and resident DCs (CD8α^{+} CD103^{-} in CD11c^{+}). The gating strategy for all samples was established on single cells after excluding large clumps, cell aggregates, and cell debris (FSC-H and FSC-A gating), and dead cells (live/dead gating).

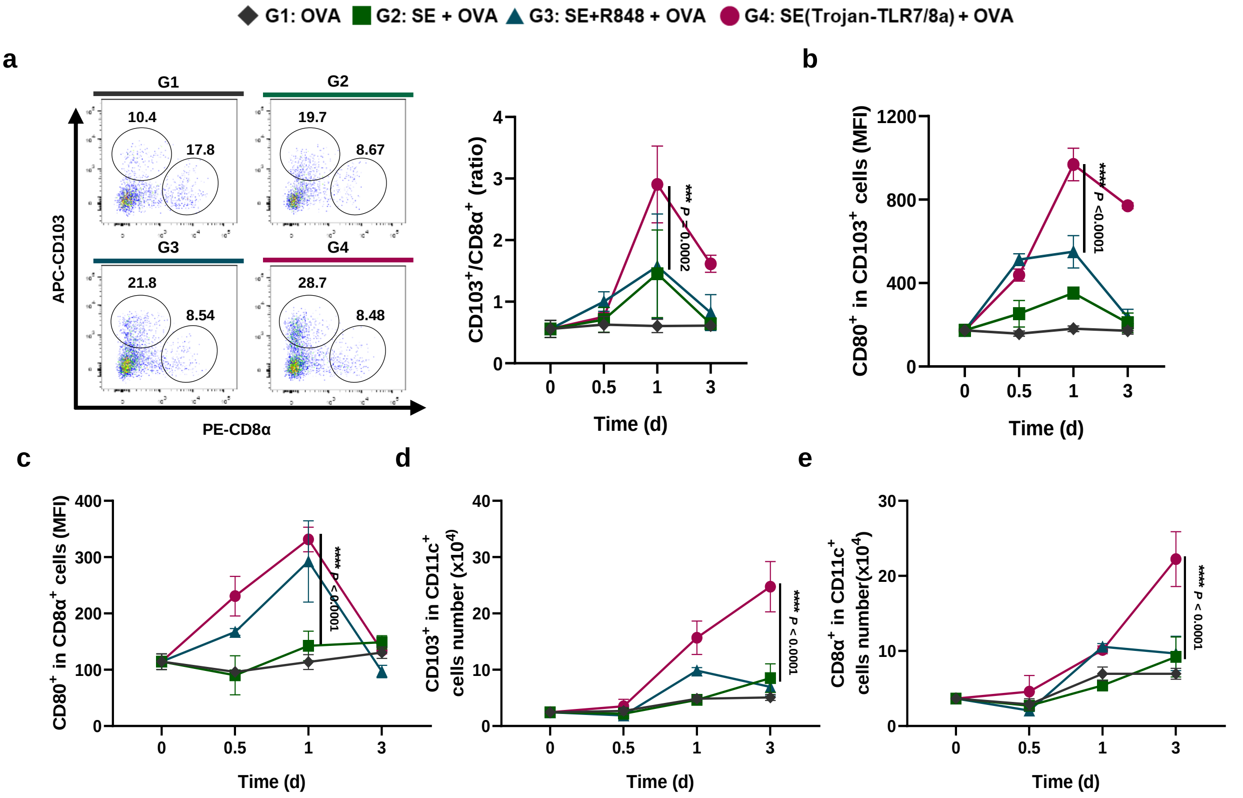

Supplementary Figure 9. Characterization of migratory and resident DCs in inguinal lymph nodes (iLNs). C57BL/6 mice were immunized intramuscularly with SE, SE+R848 (25 μg, 79.5 nmol), or SE(Trojan-TLR7/8a) (72.1 μg, 79.5 nmol), each mixed with OVA (20 μg). iLNs were excised at day 0.5, 1, and 3 (n = 3 mice per group). **a,** Representative flow cytometry plots from Day 1 and the proportion of migratory DCs ($CD103^+$) in iLNs at day 0.5, 1, and 3. **b,** The MFI of CD80 expression on migratory DCs in iLNs at day 0.5, 1, and 3. **c,** The MFI of CD80 expression on resident DCs ($CD8\alpha^+$ in $CD11c^+$) in iLNs at day 0.5, 1, and 3. **d,e,** The number of migratory DCs ($CD103^+$ in $CD11c^+$) (**d**) and resident DCs (**e**) in iLNs at day 0.5, 1, and 3. The data are presented as mean ± s.d. These data represent the same experimental results as Figure 2i; however, the addition of group 3 result in different statistical values. In **a-e,** analysis was performed by two-way ANOVA with Tukey's multiple comparison test. P values are indicated (n.s., not significant; * $P < 0.05$, ** $P < 0.01$, *** $P < 0.001$, **** $P < 0.0001$).

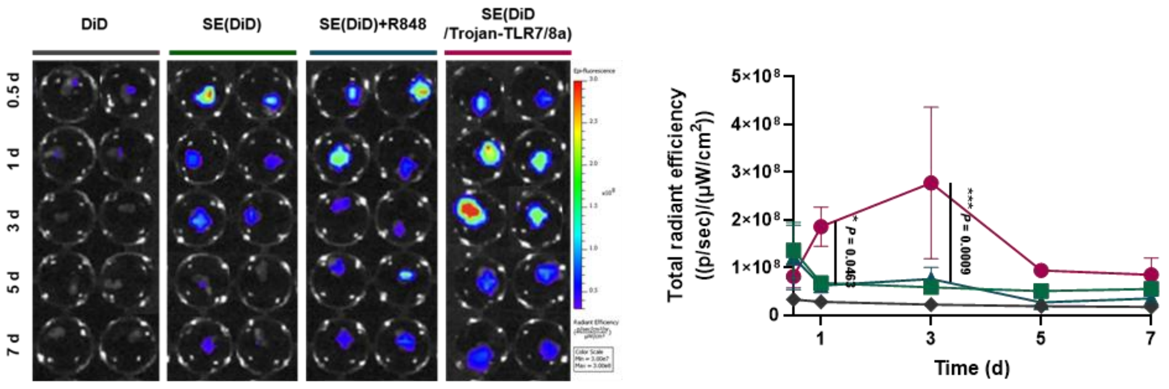

Supplementary Figure 10. Fluorescence imaging of squalene-based nanoemulsion in iLNs. C57BL/6 mice were immunized intramuscularly with DiD-loaded SE, DiD-loaded SE+R848, or DiD-loaded SE(Trojan-TLR7/8a), each mixed with OVA-FITC (40 μg). The iLNs were excised at day 0.5, 1, 3, 5, and 7. The squalene-based nanoemulsion fluorescence image of excised iLNs (left) and total radiant efficiency (left) (*n* = 2 mice per group) were measured by In Vivo Imaging System (IVIS). The data are presented as mean ± s.d. Analysis was performed by two-way ANOVA with Tukey's multiple comparison test. *P* values are indicated (n.s., not significant; * *P* < 0.05, ** *P* < 0.01, *** *P* < 0.001, **** *P* < 0.0001).

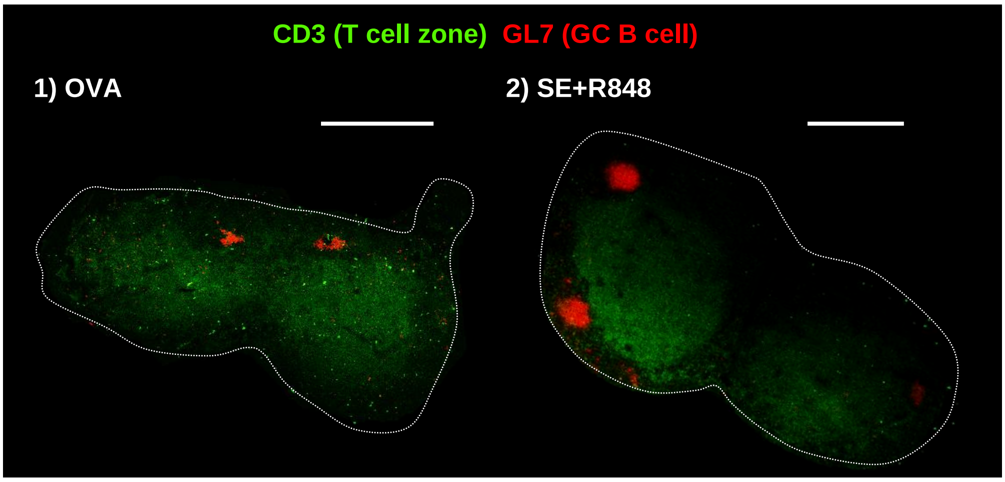

Supplementary Figure 11. Representative fluorescence imaging of iLN section showing germinal center (GC) response. C57BL/6 mice were immunized intramuscularly with OVA (20 µg) or OVA in combination with SE+R848 (25 µg, 79.5 nmol). The immunofluorescence image of sectioned iLNs at day 7, as observed with confocal laser scanning microscopy, showed the distribution T cells (CD3, green) and GC B cells (GL7, red). Scale bar, 500 µm.

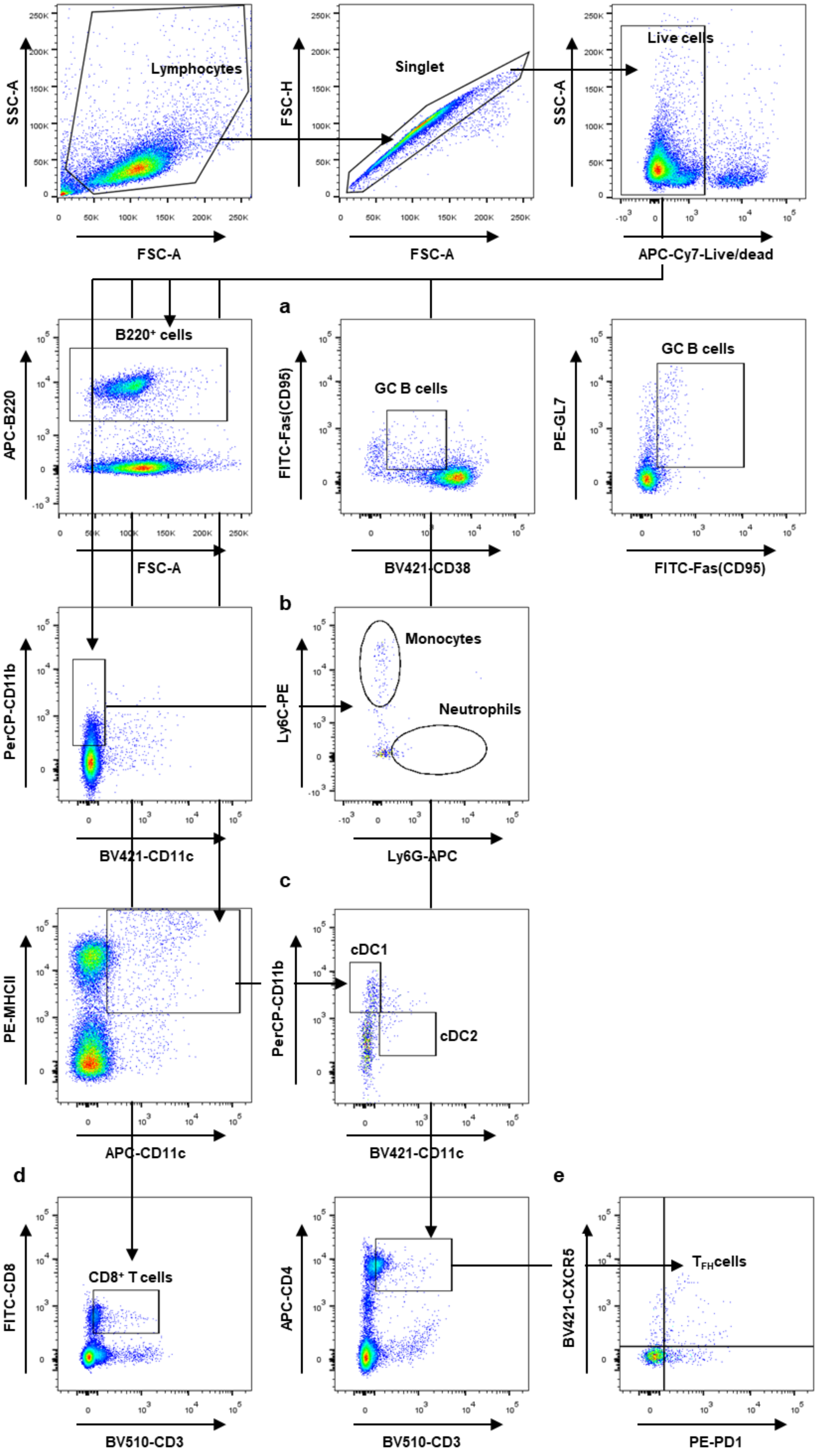
Lymphocytes
SSC-A
FSC-A
Singlet
FSC-H
Live cells
APC-Cy7-Live/dead
a
B220⁺ cells
APC-B220
GC B cells
FITC-Fas(CD95)
BV421-CD38
PE-GL7
b
Monocytes
Neutrophils
PerCP-CD11b
BV421-CD11c
Ly6C-PE
Ly6G-APC
c
PE-MHCII
APC-CD11c
cDC1
cDC2
d
CD8⁺ T cells
FITC-CD8
BV510-CD3
APC-CD4
e
TFH cells
BV421-CXCR5
PE-PD1

Supplementary Figure 12. Flow cytometry gating strategy for the analysis of immune cells *in vivo* in iLNs. **a,** Populations of GC B cells (CD38$^-$ Fas$^+$ or Fas$^+$ GL7$^+$). **b,** Populations of neutrophils (Ly6G$^+$ Ly6C$^-$) and monocytes (Ly6G$^-$ Ly6C$^+$) in CD11c$^+$ CD11b$^-$ cells. **c,** Populations of cDC2 (XCR1$^-$ CD11b$^+$ in MHCII$^+$ CD11c$^+$) or cDC1 (XCR1$^+$ CD11b$^-$ in MHCII$^+$ CD11c$^+$). **d,** Populations of CD8$^+$ T cells (CD3$^+$ CD8$^+$). **e,** Populations of T_{FH} cells (CXCR5$^+$ PD1$^+$ in CD3$^+$ CD4$^+$). The gating strategy for all samples was established on single cells after excluding large clumps, cell aggregates, and cell debris (FSC-H and FSC-A gating), and dead cells (live/dead gating).

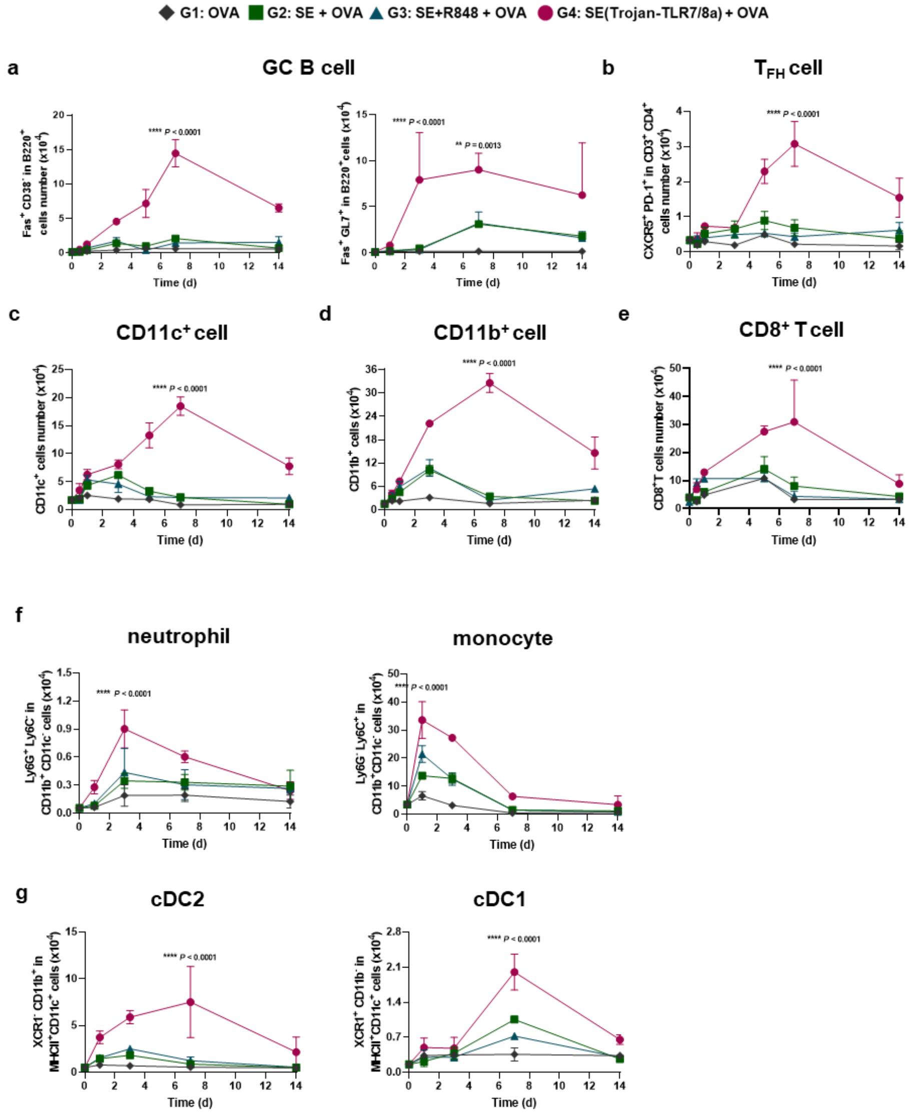

Supplementary Figure 13. The total number of various immune cells kinetics in iLNs after immunization. C57BL/6 mice were immunized intramuscularly with SE, SE+R848 (25 µg, 79.5 nmol), or SE(Trojan-TLR7/8a) (72.1 µg, 79.5 nmol), each mixed with OVA (20 µg). **a-g,** Total number of GC B cells (Fas$^+$ CD38$^-$ in B220$^+$ or Fas$^+$ GL7$^+$ in B220$^+$) (**a**), T$_{FH}$ cells (CXCR5$^+$ PD-1$^+$ in CD3$^+$ CD4$^+$) (**b**), DCs (CD11c$^+$) (**c**), macrophages (CD11b$^+$) (**d**), CD8$^+$ T cells (**e**), neutrophil (Ly6C$^-$ Ly6G$^+$ in CD11b$^+$ CD11c$^-$) or monocyte (Ly6C$^+$ Ly6G$^-$ in CD11b$^+$ CD11c$^-$) (**f**), and cDC2 (XCR1$^-$ CD11b$^+$ in MHCII$^+$ CD11c$^+$) or cDC1 (XCR1$^+$ CD11b$^-$ in MHCII$^+$ CD11c$^+$) (**g**) are measured in iLNs at day 0.5, 1, 3, 7, and 14 after immunization (n = 3 mice per group). The data are presented as mean ± s.d. These data represent the same experimental results as Figure 2o-p; however, the addition of group 3 result in different statistical values. In **a-g,** analysis was performed by two-way ANOVA with Tukey's multiple comparison test. P values are indicated (n.s., not significant; * $P < 0.05$, ** $P < 0.01$, *** $P < 0.001$, **** $P < 0.0001$) The values displayed in the graph represent the statistical comparison between SE and SE (Trojan-TLR7/8a).

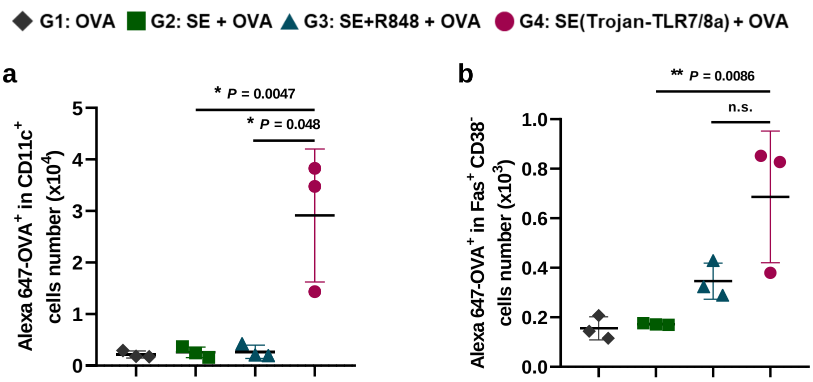

Supplementary Figure 14. The total number of Alexa-647-OVA^{+} DCs and GC B cells in iLNs after immunization. C57BL/6 mice were immunized intramuscularly with SE, SE+R848 (25 µg, 79.5 nmol), or SE(Trojan-TLR7/8a) (72.1 µg, 79.5 nmol), each mixed with Alexa 647-OVA (20 µg). **a,b,** The total number of Alexa 647-OVA^{+} DCs (**a**), and GC B cells (**b**) were evaluated at day 5, respectively (n = 3 mice per group). The data are presented as mean ± s.d. In **a,** and **b,** analysis was performed by one-way ANOVA with Tukey's multiple comparison test. P values are indicated (n.s., not significant; * $P < 0.05$, ** $P < 0.01$, *** $P < 0.001$, **** $P < 0.0001$).

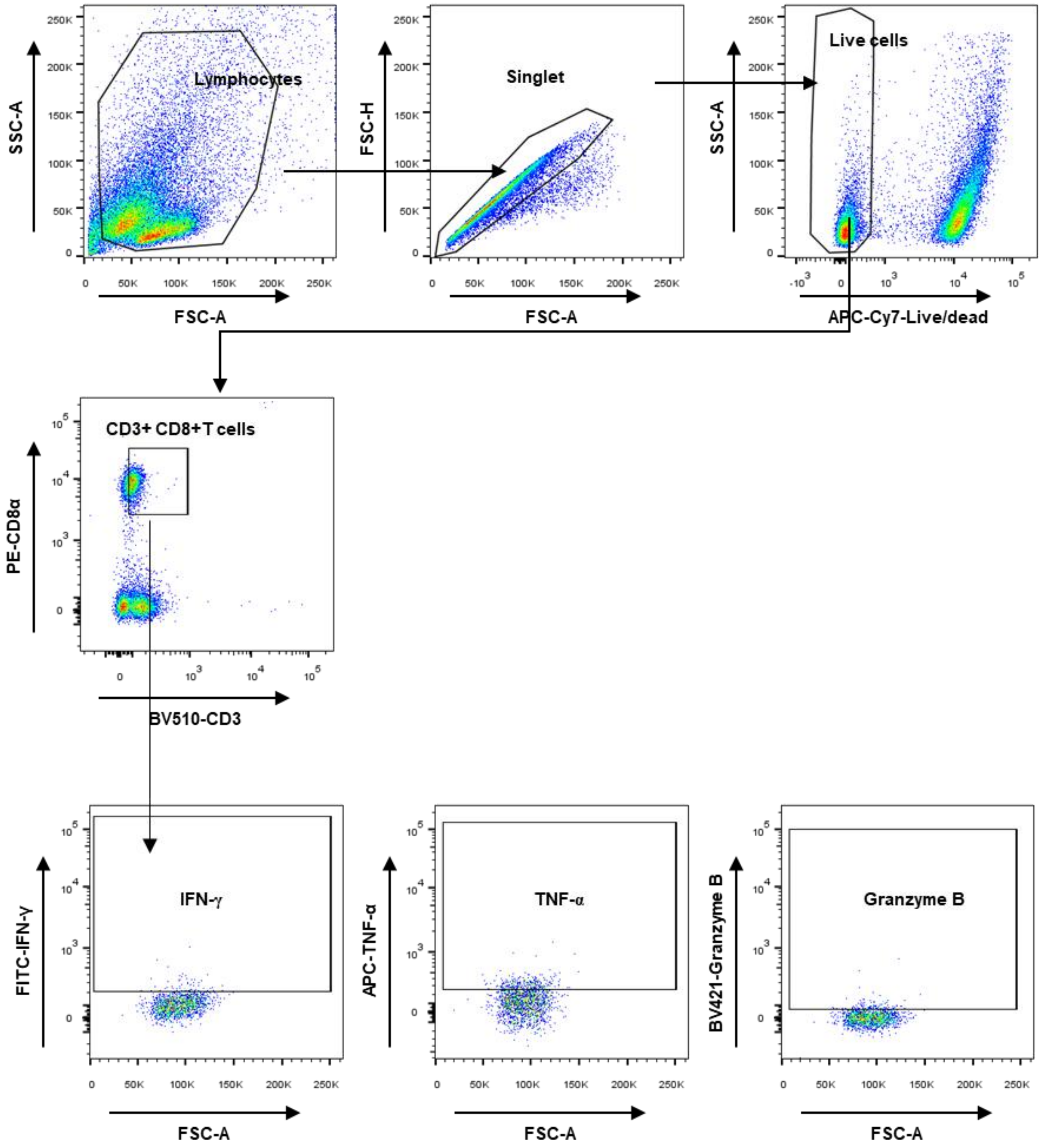

Supplementary Figure 15. Flow cytometry gating strategy for the analysis of immune cells in iLNs. Population of cytokine secreting CD8$^+$ T cells (IFN-γ$^+$, TNF-α$^+$, or granzyme B$^+$ in CD3$^+$ CD8$^+$). The gating strategy for all samples was established on single cells after excluding large clumps, cell aggregates, cell debris (FSC-H and FSC-A gating), and dead cells (live/dead gating).

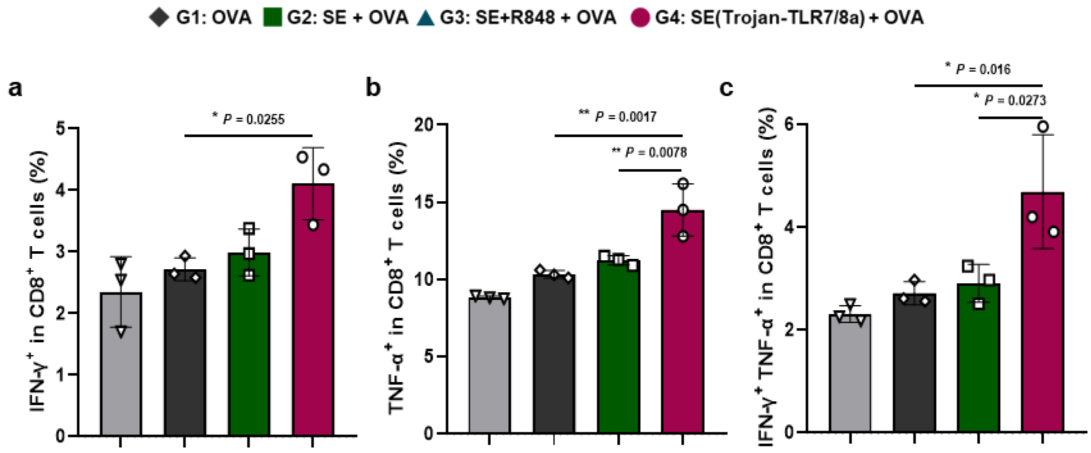

Supplementary Figure 16. Population of antigen-specific cytokine-secreting CD8⁺ T cells in iLNs after immunization. C57BL/6 mice were immunized intramuscularly with SE, SE+R848 (25 µg, 79.5 nmol), or SE(Trojan-TLR7/8a) (72.1 µg, 79.5 nmol), each mixed with Alexa 647-OVA (20 µg). **a-c,** The total number of IFN-γ (**a**), TNF-α (**b**), and polyfunctional (doble positive for IFN-γ and TNF-α) (**c**) CD8⁺ T cells were evaluated at day 7 (*n* = 3 mice per group). The data are presented as mean ± s.d. The data are presented as mean ± s.d. These data represent the same experimental results as Figure 2q; however, the addition of group 3 result in different statistical values. In **a-c,** analysis was performed by one-way ANOVA with Tukey's multiple comparison test. *P* values are indicated (n.s., not significant; * $P < 0.05$, ** $P < 0.01$, *** $P < 0.001$, **** $P < 0.0001$).

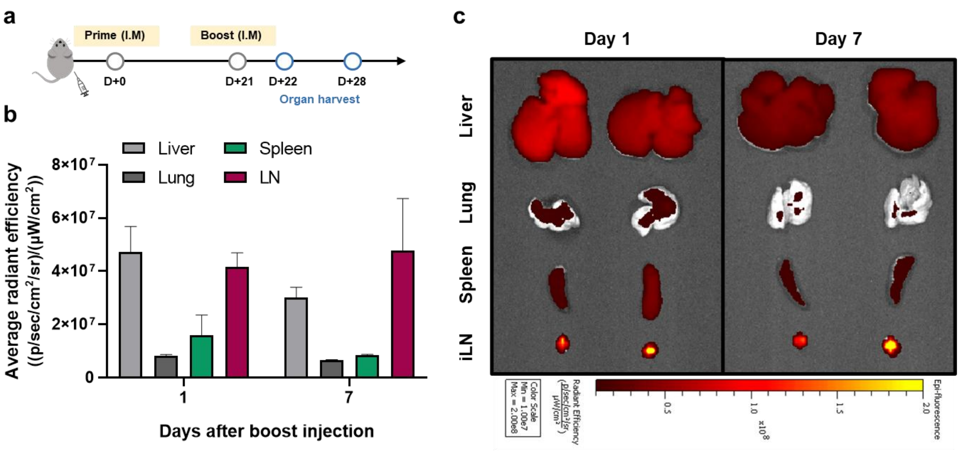

Supplementary Figure 17. Organ biodistribution after prime-boost injection of SE(DiD/Trojan-TLR7/8a). a, C57BL/6 mice were immunized intramuscularly with OVA in combination with SE(DiD/Trojan-TLR7/8a) (72.1 μg, 79.5 nmol) in the hind leg twice at 3-week intervals. On day 22 and 28, various organs (liver, spleen, lung, liver) were dissected. **b,** Average radiant efficiency (right) and **c,** fluorescence imaging of SE(DiD/Trojan-TLR7/8a) in each excised organs (right) (n = 2 mice per group) obtained using In Vivo Imaging System (IVIS).

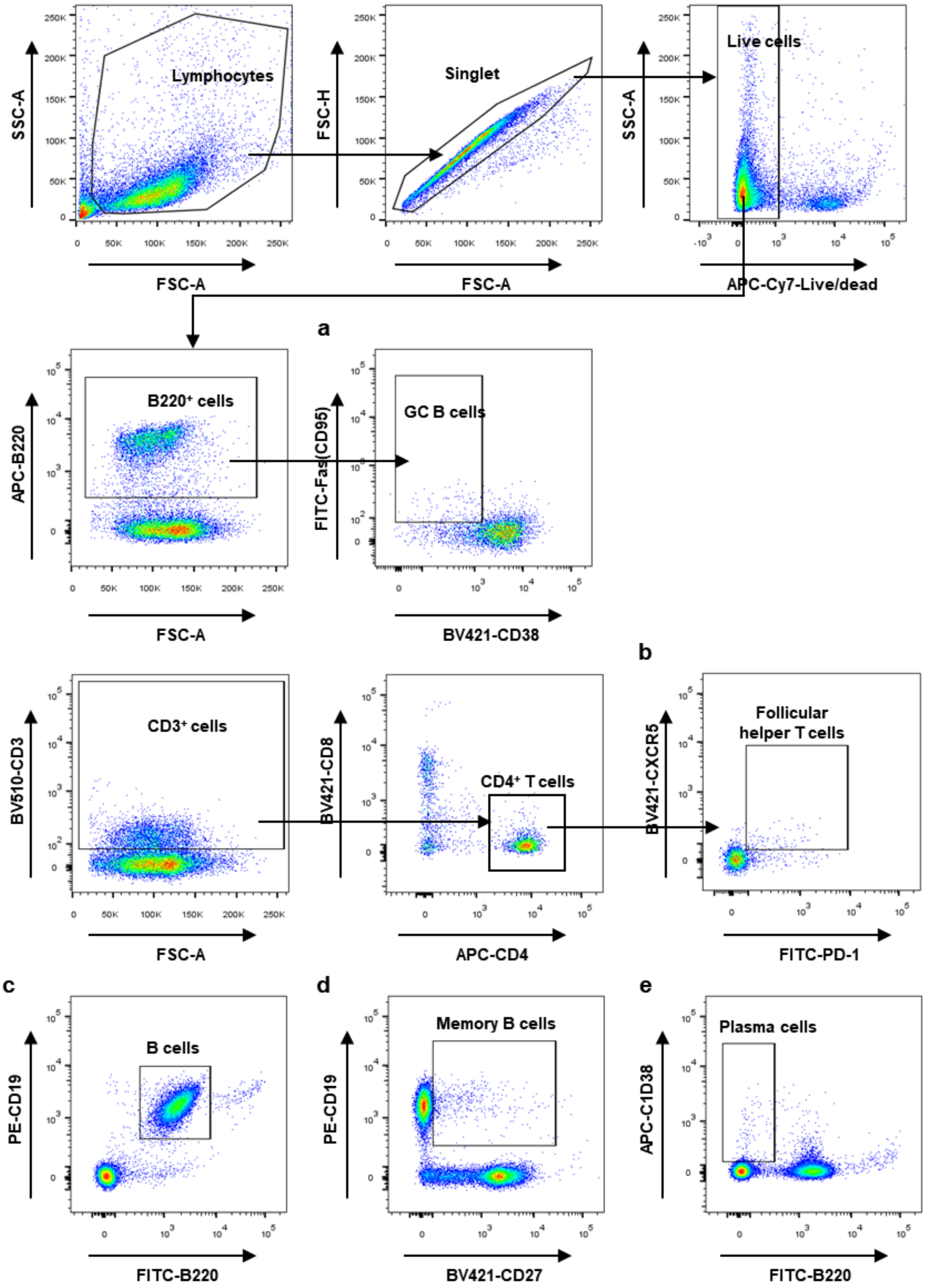

Supplementary Figure 18. Flow cytometry gating strategy for the analysis of immune cells. **a,** Population of GC B cells (CD38$^-$ CD95$^+$ in B220$^+$). **b,** Population of follicular helper T cells (T_{FH}, PD-1$^+$ CXCR5$^+$ in CD3$^+$ CD4$^+$). **c-e,** Populations of B cells (CD19$^+$ B220$^+$) (**c**), memory B cells (CD19$^+$ CD27$^+$) (**d**), and plasma cells (B220$^-$ CD138$^+$) (**e**). The gating strategy for all samples was established on single cells after excluding large clumps, cell aggregates, cell debris (FSC-H and FSC-A gating), and dead cells (live/dead gating).

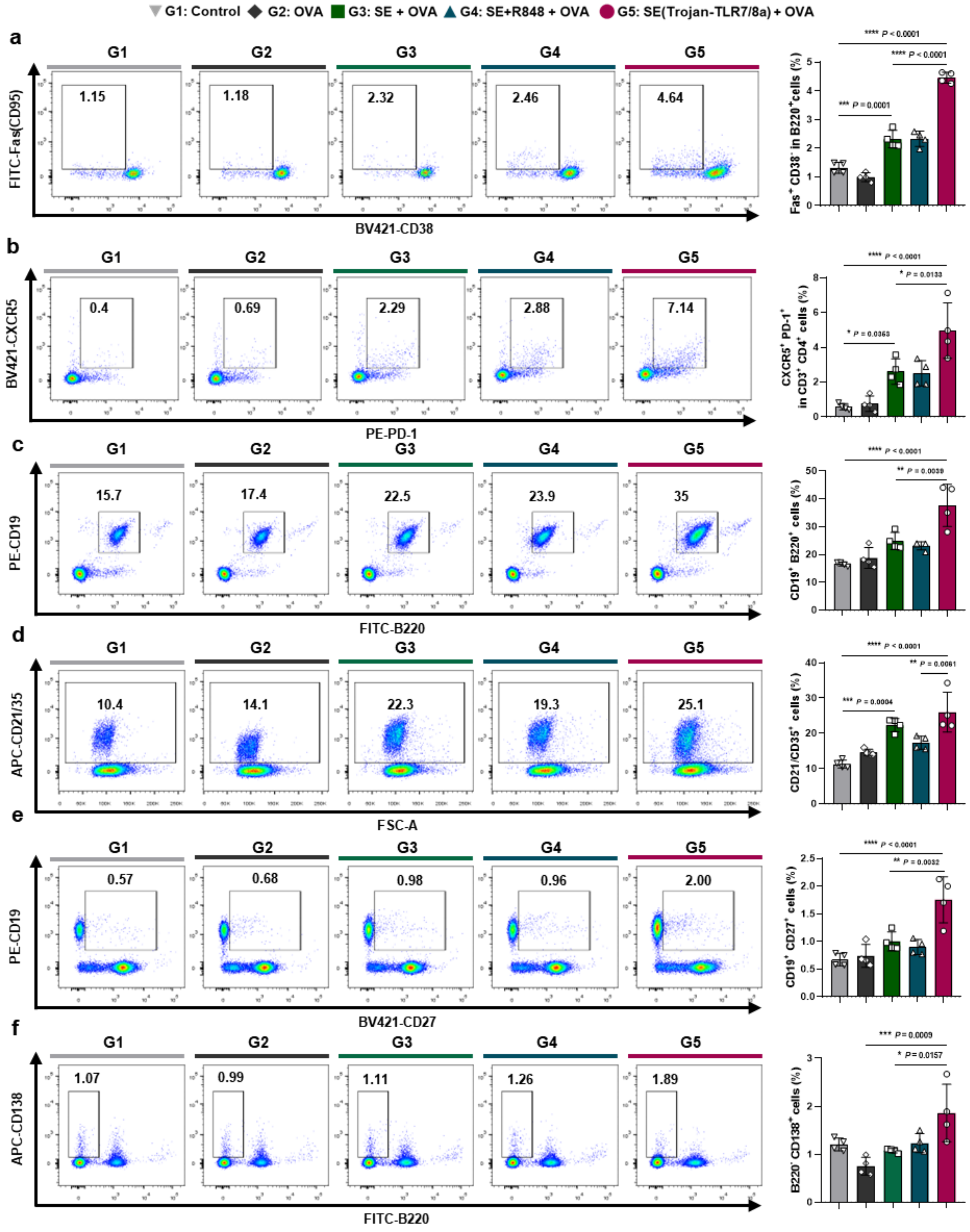

Supplementary Figure 19. SE(Trojan-TLR7/8a) enhances the magnitude of humoral responses in iLNs. C57BL/6 mice were immunized intramuscularly with OVA (20 μg) or OVA in combination with SE, SE+R848 (25 μg, 79.5 nmol), or SE(Trojan-TLR7/8a) (72.1 μg, 79.5 nmol) in the hind leg twice at 3-week intervals. On day 28, iLNs were harvested and processed to generate single-cell suspensions. **a-f**, Representative flow cytometry plots and percentage of GC B cells ($CD38^{-}$ Fas^{+} in $B220^{+}$) (**a**), TFH cells ($CXCR5^{+}$ $PD\text{-}1^{+}$ in $CD3^{+}$ $CD4^{+}$) (**b**), B cells ($CD19^{+}$ $B220^{+}$) (**c**), FDCs ($CD21/CD35^{+}$) (**d**), memory B cells

(CD19⁺ CD27⁺) (**e**), and plasma cells (B220⁻ CD138⁺) (**f**) (*n* = 4 mice per group). The data are presented as mean ± s.d. These data represent the same experimental results as Figure 3b; however, the addition of group 4 may result in different statistical values. In **a-f,** analysis was performed by one-way ANOVA with Tukey's multiple comparison test. *P* values are indicated (n.s., not significant; * $P < 0.05$, ** $P < 0.01$, *** $P < 0.001$, **** $P < 0.0001$).

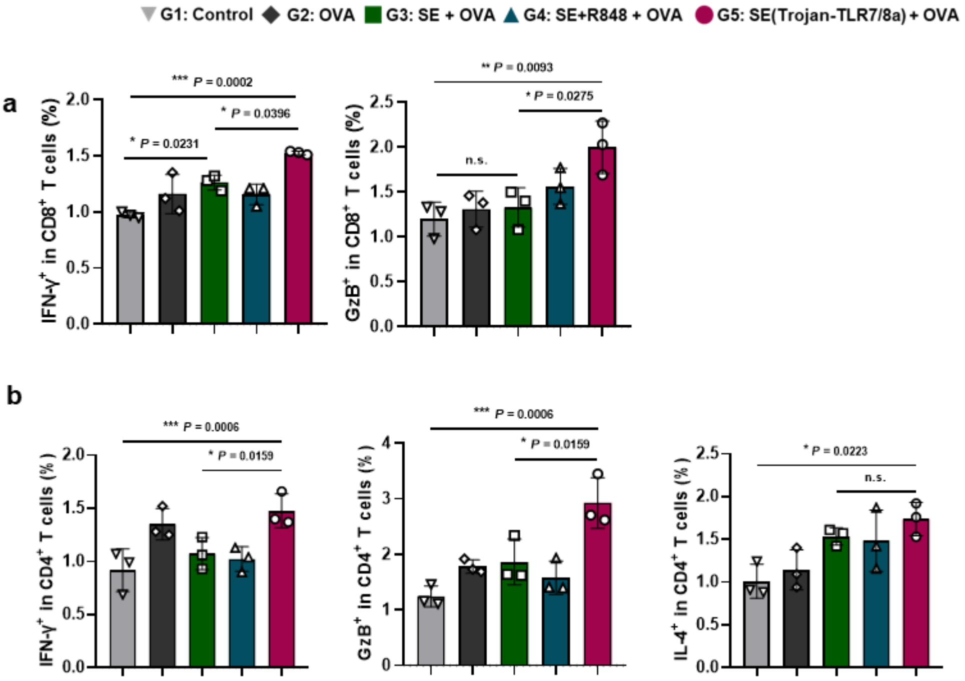

Supplementary Figure 20. SE(Trojan-TLR7/8a) enhances the magnitude of cellular responses in iLNs. C57BL/6 mice were immunized intramuscularly with OVA (20 μg) or OVA in combination with SE, SE+R848 (25 μg, 79.5 nmol), or SE(Trojan-TLR7/8a) (72.1 μg, 79.5 nmol) in the hind leg twice at 3-week intervals. On day 28, iLNs were harvested and processed to generate single-cell suspensions. Cells were restimulated with $OVA_{323\text{-}339}$ peptide (10 μg ml^{-1}) and incubated with GolgiPlug in the presence of IL-2 (30 ng ml^{-1}) for 12 h. **a,b,** Percentage of IFN-γ⁺ or GzB⁺ in antigen-specific CD8⁺ T cells (**a**) and percentage of IFN-γ⁺, GzB⁺, or IL-4⁺ in antigen-specific CD4⁺ T cells (**b**) (*n* = 3 mice per group). The data are presented as mean ± s.d. These data represent the same experimental results as Figure 3c; however, the addition of group 3 result in different statistical values. In **a,** and **b,** analysis was performed by one-way ANOVA with Tukey's multiple comparison test. *P* values are indicated (n.s., not significant; * $P < 0.05$, ** $P < 0.01$, *** $P < 0.001$, **** $P < 0.0001$).

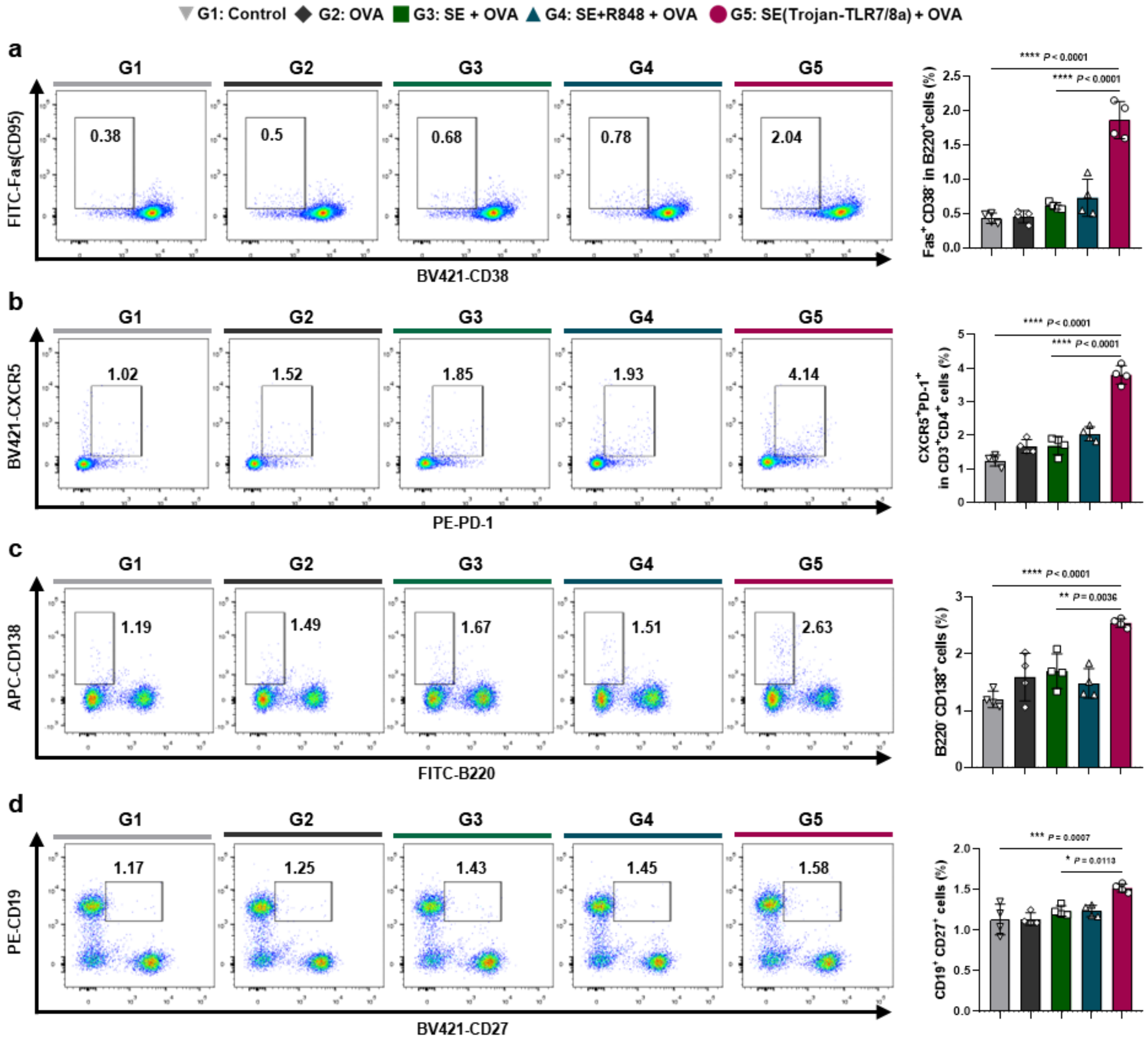

Supplementary Figure 21. SE(Trojan-TLR7/8a) enhances the magnitude of humoral responses in spleens. C57BL/6 mice were immunized intramuscularly with OVA (20 μg) or OVA in combination with SE, SE+R848 (25 μg, 79.5 nmol), SE(Trojan-TLR7/8a) (72.1 μg, 79.5 nmol) in the hind leg twice at 3-week intervals. On day 28, spleens were harvested and processed to generate single-cell suspensions. **a-d,** Representative flow cytometry plots and percentage of GC B cells ($CD38^{-}$ Fas^{+} in $B220^{+}$) (**a**), TFH cells ($CXCR5^{+}$ $PD\text{-}1^{+}$ in $CD3^{+}$ $CD4^{+}$) (**b**), plasma cells ($B220^{-}$ $CD138^{+}$) (**c**), and memory B cells ($CD19^{+}$ $CD27^{+}$) (**d**) ($n = 4$). The data are presented as mean ± s.d. These data represent the same experimental results as Figure 3e; however, the addition of group 3 result in different statistical values. In **a-d,** analysis was performed by one-way ANOVA with Tukey's multiple comparison test. *P* values are indicated (n.s., not significant; * $P < 0.05$, ** $P < 0.01$, *** $P < 0.001$, **** $P < 0.0001$).

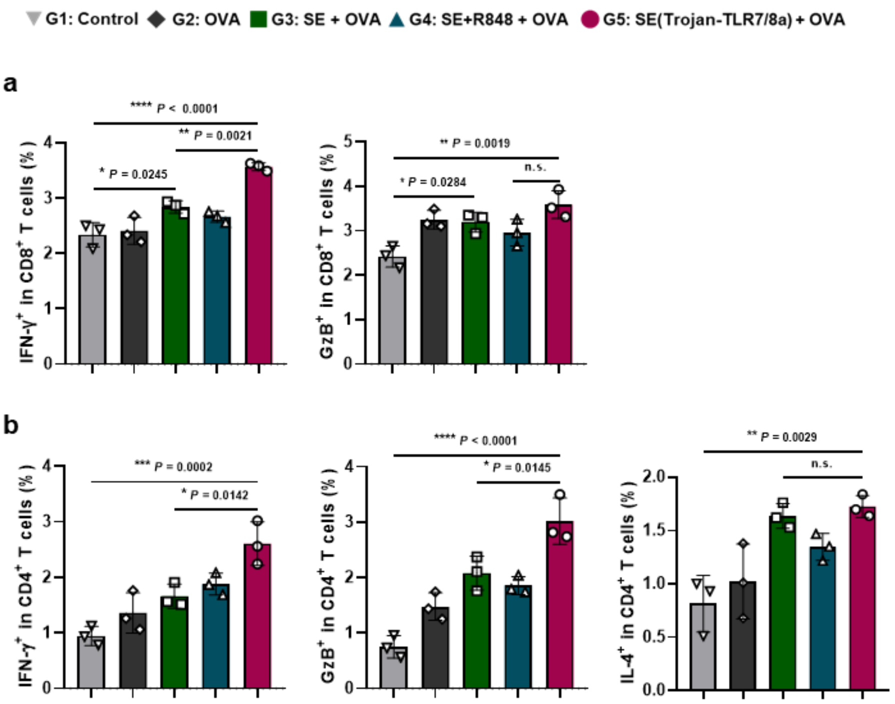

Supplementary Figure 22. SE(Trojan-TLR7/8a) enhances the magnitude of cellular responses in spleens. C57BL/6 mice were immunized intramuscularly with OVA (20 µg) or OVA in combination with SE, SE+R848 (25 µg, 79.5 nmol), SE(Trojan-TLR7/8a) (72.1 µg, 79.5 nmol) in the hind leg twice at 3-week intervals. On day 28, spleens were harvested and processed to generate single-cell suspensions. Single cells were restimulated with $OVA_{257-264}$ peptide or $OVA_{323-339}$ peptide (10 µg ml^{-1}) and incubated with GolgiPlug in the presence of IL-2 (30 ng ml^{-1}) for 12 h. **a,b,** Percentage of IFN-γ^+ or GzB^+ in antigen-specific $CD8^+$ T cells (**a**) and percentage of IFN-γ^+, GzB^+, or IL-4^+ in antigen-specific $CD4^+$ T cells (**b**) (n = 3 mice per group). The data are presented as mean ± s.d. These data represent the same experimental results as Figure 3e; however, the addition of group 3 result in different statistical values. In **a,** and **b,** analysis was performed by one-way ANOVA with Tukey's multiple comparison test. *P* values are indicated (n.s., not significant; * $P < 0.05$, ** $P < 0.01$, *** $P < 0.001$, **** $P < 0.0001$).

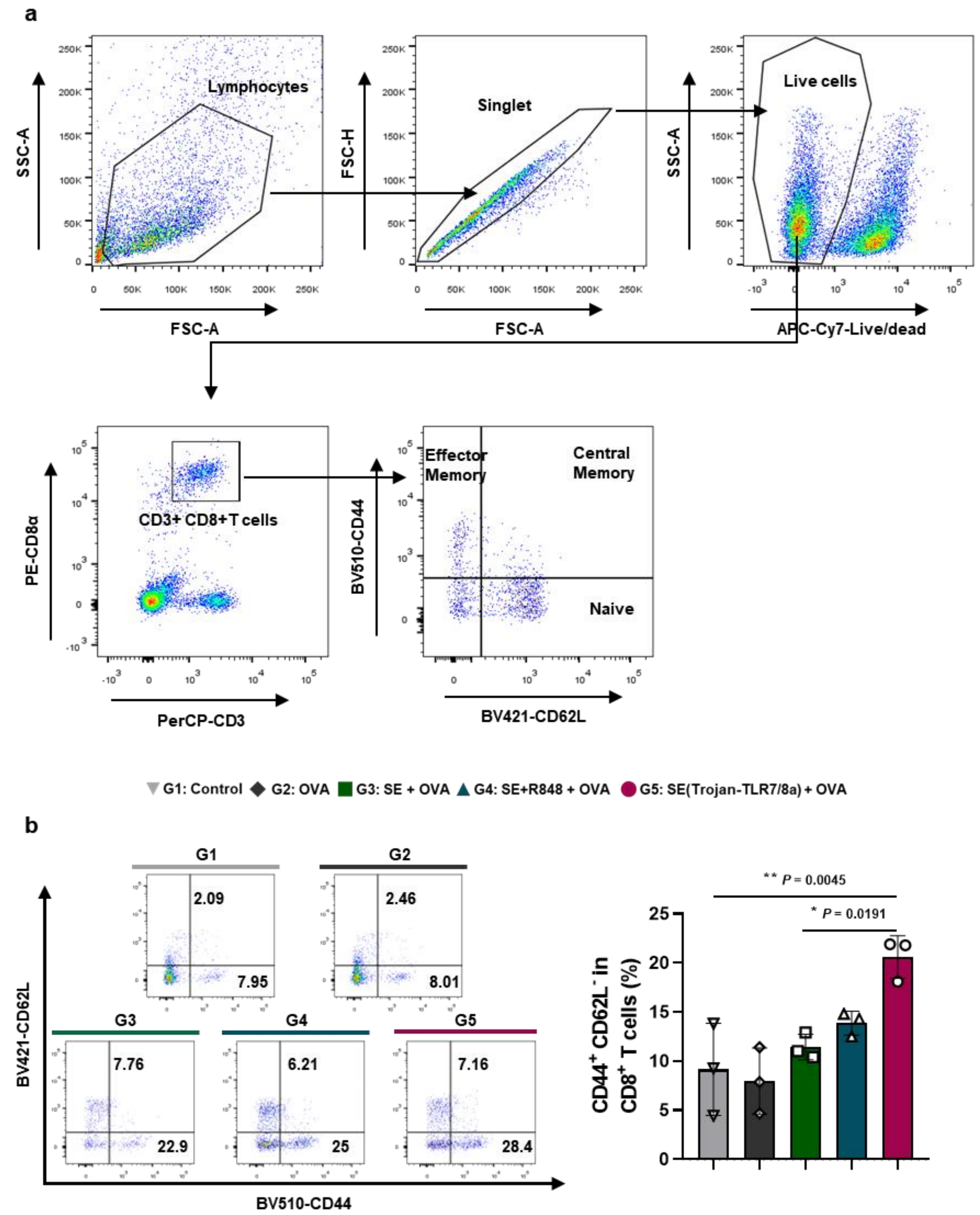

Supplementary Figure 23. Flow cytometry gating strategy for the analysis of immune cells in lungs. **a,** Populations of central memory CD8$^+$ T cells (CD44$^+$ CD62L$^+$ in CD3$^+$ CD8$^+$) and effector memory CD8$^+$ T cells (CD44$^+$ CD62L$^-$ in CD3$^+$ CD8$^+$). The gating strategy for all samples was established on single cells after excluding large clumps, cell aggregates, cell debris (FSC-H and FSC-A gating), and dead cells (live/dead gating). **b,** Representative flow cytometry plots and percentage of effector memory CD8$^+$ T cells (CD44$^+$ CD62L$^-$ in CD3$^+$ CD8$^+$) in lungs at 4-week (n = 3 mice per group). The data are presented as mean ± s.d. These data represent the same experimental results as Figure 3g; however, the addition of group 3 result in different statistical values. In **b,** analysis was performed by one-way ANOVA with Tukey's multiple

comparison test. *P* values are indicated (n.s., not significant; * $P < 0.05$, ** $P < 0.01$, *** $P < 0.001$, **** $P < 0.0001$).

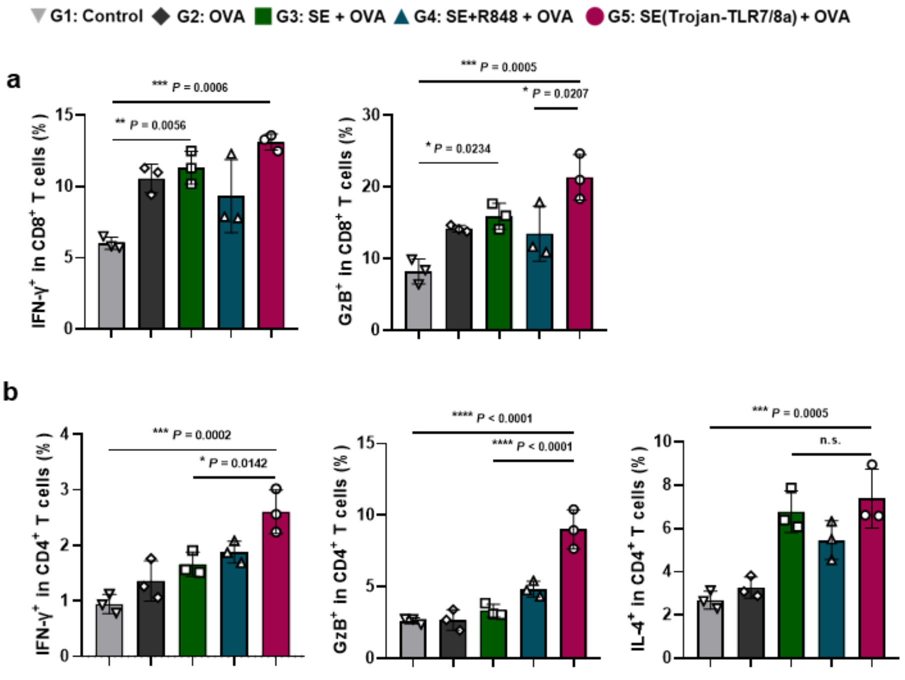

Supplementary Figure 24. SE(Trojan-TLR7/8a) enhances the magnitude and quality of cellular responses in lungs. C57BL/6 mice were immunized intramuscularly with OVA (20 μg) or OVA in combination with SE, SE+R848 (25 μg, 79.5 nmol), or SE(Trojan-TLR7/8a) (72.1 μg, 79.5 nmol) in the hind leg twice at 3-week intervals. On day 28, lungs were harvested and processed to generate single-cell suspensions. Single cells were restimulated $OVA_{257-264}$ peptide or $OVA_{323-339}$ peptide (10 μg ml^{-1}) and incubated with GolgiPlug in the presence of IL-2 (30 ng ml^{-1}) for 12 h. **a,b,** Percentage of IFN-γ^+ or GzB^+ in antigen-specific $CD8^+$ T cells (**a**) and percentage of IFN-γ^+, GzB^+, or IL-4^+ in antigen-specific $CD4^+$ T cells (**b**) (*n* = 3 mice per group). The data are presented as mean ± s.d. These data represent the same experimental results as Figure 3h; however, the addition of group 3 result in different statistical values. In **a,** and **b,** analysis was performed by one-way ANOVA with Tukey's multiple comparison test. *P* values are indicated (n.s., not significant; * $P < 0.05$, ** $P < 0.01$, *** $P < 0.001$, **** $P < 0.0001$).

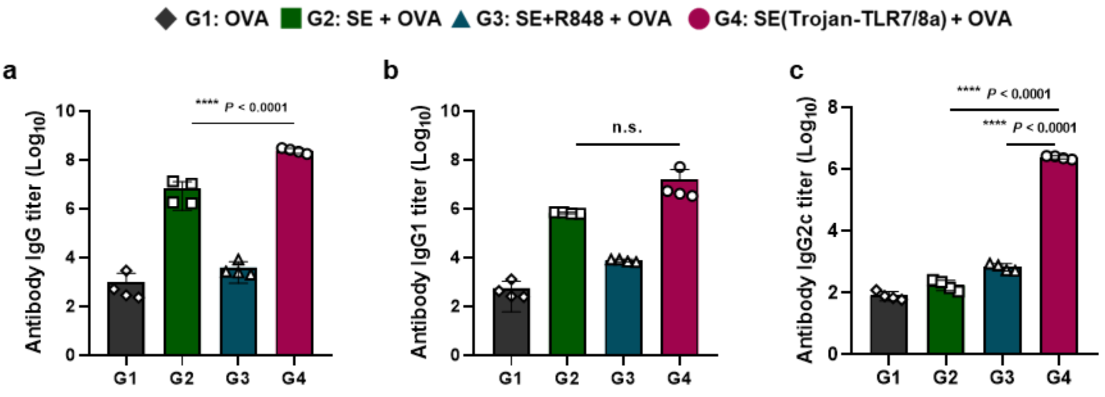

Supplementary Figure 25. Serum OVA-specific ELISA analysis after immunization. a-c, C57BL/6 mice were immunized intramuscularly with OVA (20 µg) or OVA in combination with SE , SE+R848 (25 µg, 79.5 nmol), SE(Trojan-TLR7/8a) (72.1 µg, 79.5 nmol) in the hind leg twice at 3-week intervals. On day 28, blood was collected. Serum was obtained after being centrifuged at 10,000g for 10 min at 4 °C and analysed by ELISA for OVA-specific IgG (**a**), IgG1 (**b**), and IgG2c (**c**) antibodies (n = 4 mice per group). The data are presented as mean ± s.d. These data represent the same experimental results as Figure 3i; however, the addition of group 3 result in different statistical values. In **a-c,** analysis was performed by one-way ANOVA with Tukey's multiple comparison test. P values are indicated (n.s., not significant; * $P < 0.05$, ** $P < 0.01$, *** $P < 0.001$, **** $P < 0.0001$).

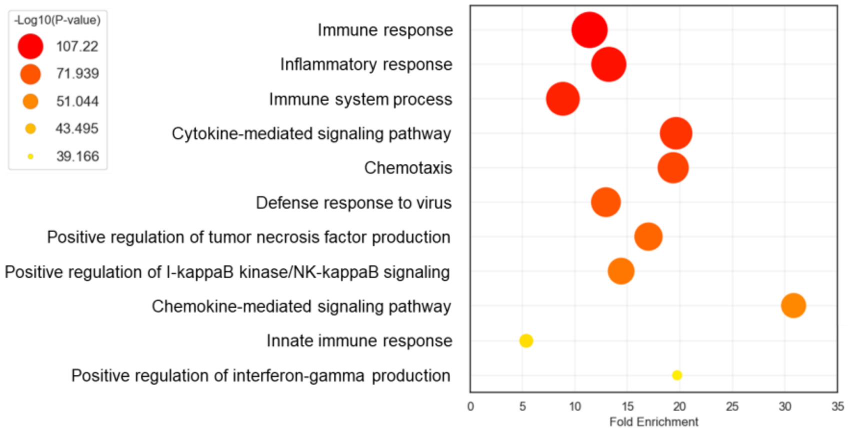

Supplementary Figure 26. DAVID functional GO analysis of KEGG protein enrichment. C57BL/6 mice were immunized intramuscularly with OVA in combination with SE(Trojan-TLR7/8a) (72.1 μg, 79.5 nmol) in the hind leg twice at 3-week intervals. LNs are harvester for mRNA analysis on day 28. Gene ontologies (GO) for genes significantly upregulated in SE(Trojan-TLR7/8a) versus control (n = 3 mice per group).

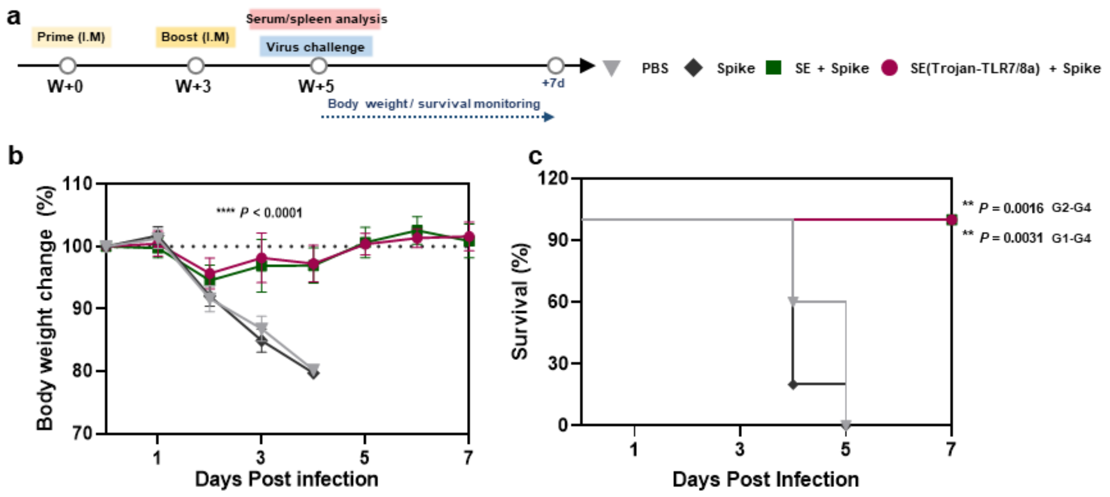

Supplementary Figure 27. The body weight changes and survival of mouse-adapted SARS-CoV-2 virus-infected mice. a, BALB/c mice were administered spike protein alone or combined with SE or SE(Trojan-TLR7/8a) on weeks 0 and 3. **b,c,** Two weeks after the last vaccination, to observe the protective efficacy of the SE(Trojan-TLR7/8a), mice were infected with 100 LD50 of mouse-adapted SARS-CoV-2 virus and their body weight (**b**) and survival rate (**c**) were monitored up to 7 days post-infection (dpi) (n = 5 mice per group). The data are presented as mean ± s.d. In **b,** analysis was performed by two-way ANOVA with Tukey's multiple comparison test. In **c,** analysis was performed by Log-rank (Mantel-Cox) test. P values are indicated (n.s., not significant; * $P < 0.05$, ** $P < 0.01$, *** $P < 0.001$, **** $P < 0.0001$).

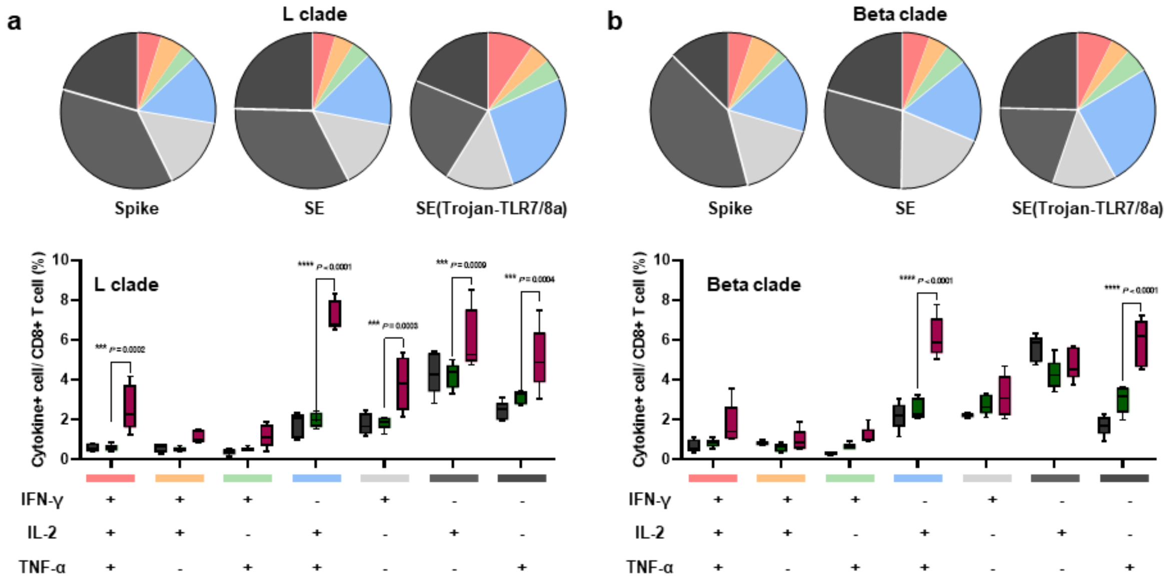

Supplementary Figure 28. Polyfunctional capacity of cytotoxicity T lymphocyte (CTL) in splenocytes. Two weeks after the last vaccination, spleens were collected from all the immunized mice. **a,b,** Flow cytometry was performed by stimulating splenocytes with L clade (**a**) or Beta clade (**b**) in the presence of monensin for 12 h. CTL response was characterized by an increase in IFN-γ, TNF-α, and IL-2 production (*n* = 5 mice per group). The data are presented as mean ± s.d. In **a,** and **b,** analysis was performed by two-way ANOVA with Tukey's multiple comparison test. *P* values are indicated (n.s., not significant; * $P < 0.05$, ** $P < 0.01$, *** $P < 0.001$, **** $P < 0.0001$).

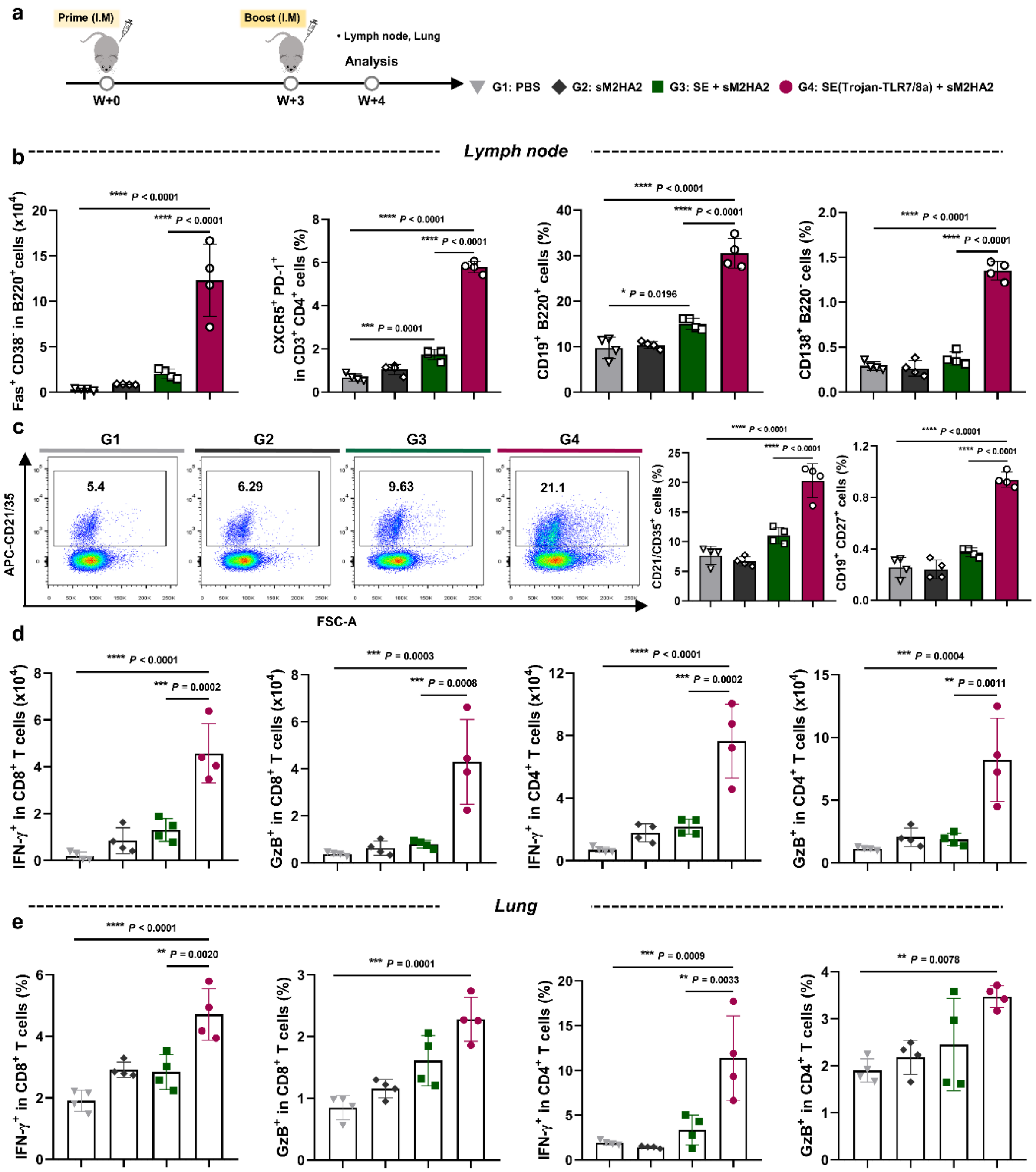

Supplementary Figure 29. Humoral and cellular responses after vaccination with sM2HA2. a, BALB/c mice were intramuscularly immunized with sM2HA2 (15 µg) alone or in combination with SE (the same volume used for SE(Trojan-TLR7/8a)) or SE(Trojan-TLR7/8a) (79.5 nmol) twice in weeks 0 and 3, after which the iLNs and lungs were analysed according to the schedule (*n* = 4 mice per group). **b-e,** Vaccines included sM2HA2 (15 µg) alone and sM2HA2 in combination with SE or SE(Trojan-TLR7/8a) (79.5 nmol). **b,** Total number of GC B cells (Fas$^+$ CD38$^-$ in B220$^+$) and populations of T_{FH} cells, B cells, plasma cells (CD138$^+$ B220$^-$), and memory B cells in the iLNs. **c,** Representative flow cytometry plots and population of FDCs. Total numbers of sM2HA2-specific CD8$^+$ or CD4$^+$ T cells secreting IFN-γ or GzB in the iLNs (**d**) and lungs (**e**). The data are presented as the mean ± s.d. In **b-e,** analysis was performed by one-way ANOVA with

Tukey's multiple comparison test. P values are indicated (n.s., not significant; * $P < 0.05$, ** $P < 0.01$, *** $P < 0.001$, **** $P < 0.0001$).

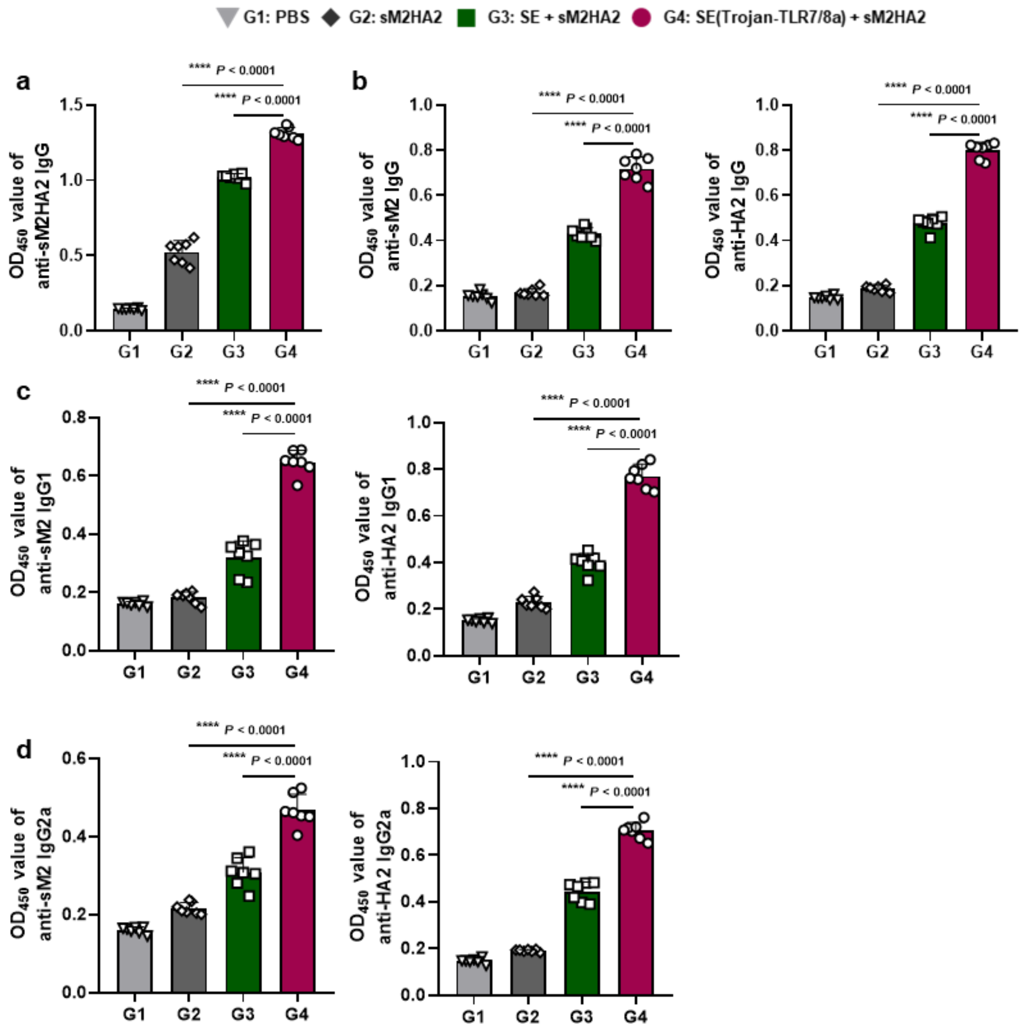

Supplementary Figure 30. Serum ELISA analysis after vaccination with sM2HA2. a-d, BALB/c mice were immunized intramuscularly with SE or SE(Trojan-TLR7/8a) (72.1 μg, 79.5 nmol), each mixed with sM2HA2 (15 μg) in the hind leg twice at 3-week intervals. sM2HA2 specific IgG antibody titers at 1:100 serum dilution ratio (**a**). Serum IgG antibody titers specific to sM2 and HA2 at 1:100 serum dilution ratio (**b**). Systemic IgG1 antibody titers specific to sM2 and HA2 at 1:100 serum dilution ratio (**c**). Systemic IgG2a antibody titers specific to sM2 and HA2 at 1:100 serum dilution ratio (**d**) ($n = 7$ mice per group). The data are presented as mean ± s.d. In **a-d,** analysis was performed by one-way ANOVA with Tukey's multiple comparison test. P values are indicated (n.s., not significant; * $P < 0.05$, ** $P < 0.01$, *** $P < 0.001$, **** $P < 0.0001$).

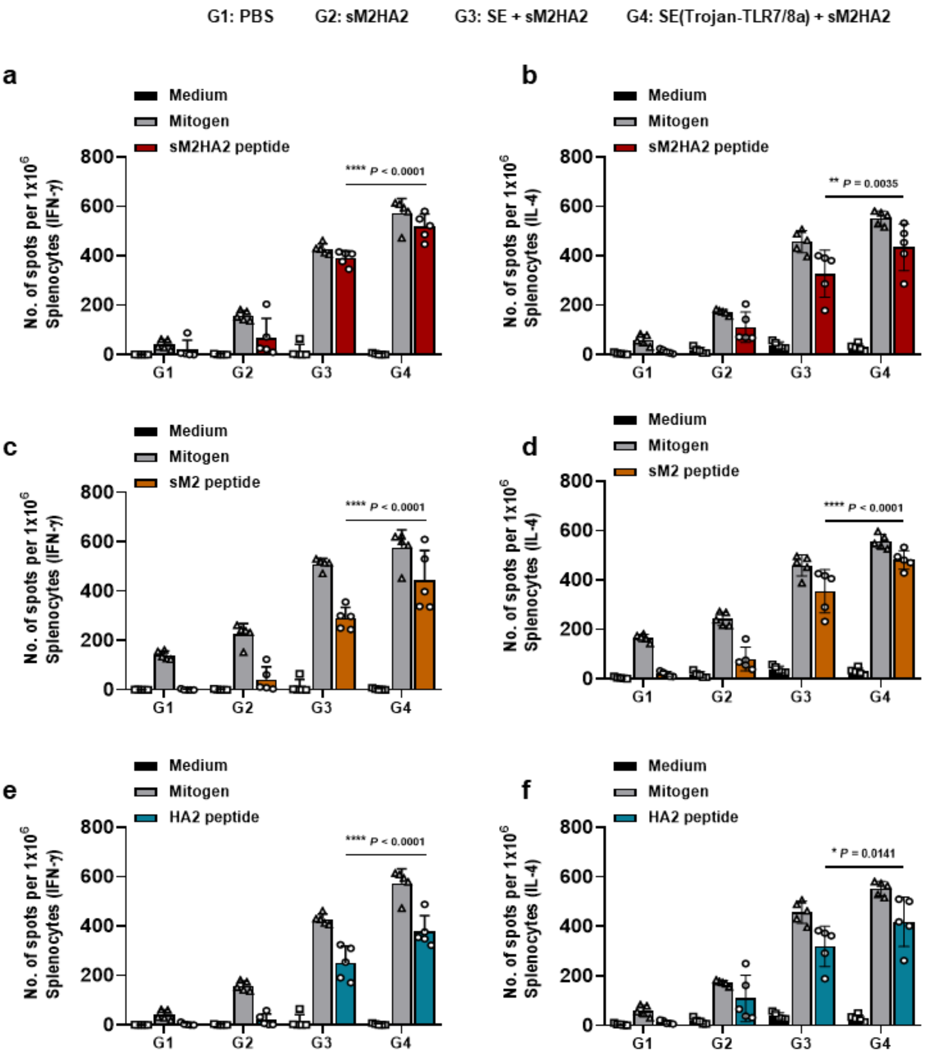

Supplementary Figure 31. T cell response SE(Trojan-TLR7/8a) adjuvanted sM2HA2. a-f, Mice were immunized intramuscularly with sM2HA2 (15 µg) or sM2HA2 in combination with SE, SE(Trojan-TLR7/8a) (72.1 µg, 79.5 nmol) in the hind leg twice at 3-week intervals. The number of sM2HA2, sM2 or HA2 specific IFN-γ spot forming units (**a, c, e**) and IL-4 spot forming units (**b, d, f**) were measured by enzyme-linked immunosorbent spot (ELISPOT) assay after collecting splenocytes at 28 post-immunization (n = 5 mice per group). The data are presented as mean ± s.d. In **a-f,** analysis was performed by two-way ANOVA with Tukey's multiple comparison test. P values are indicated (n.s., not significant; * $P < 0.05$, ** $P < 0.01$, *** $P < 0.001$, **** $P < 0.0001$).

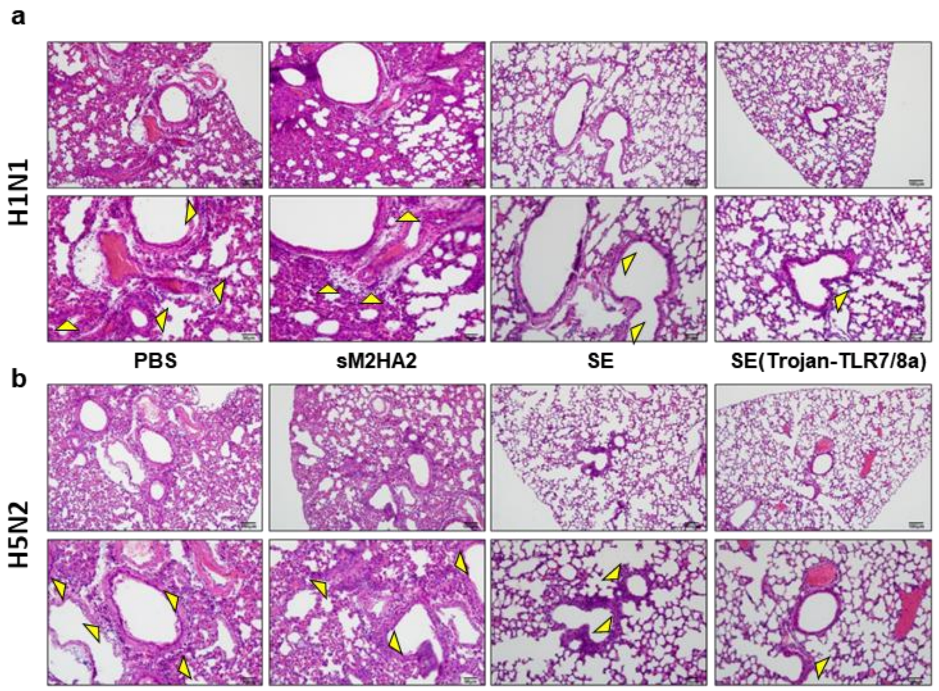

Supplementary Figure 32. Lung hematoxylin and eosin (H&E) staining after post-H1N1 and H5N2 infection. a,b, BALB/c mice were immunized intramuscularly with sM2HA2 (15 µg) or sM2HA2 in combination with SE or SE(Trojan-TLR7/8a) (72.1 µg, 79.5 nmol) in the hind leg twice at 3-week intervals and challenged with 10 LD50 of mouse-adapted influenza subtypes one week after the second immunization. H&E staining for the lung sections was collected at 5 days post-H1N1 (**a**) and H5N2 (**b**) infection. The arrows indicate the inflammatory cell infiltration.

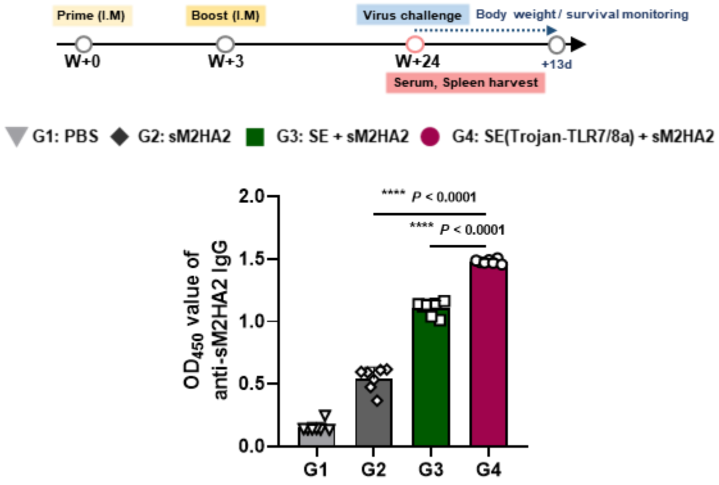

Supplementary Figure 33. Long-lasting serum sM2HA2-specific ELISA analysis after vaccination with sM2HA2. BALB/c mice were immunized intramuscularly with SE or SE(Trojan-TLR7/8a) (72.1 μg, 79.5 nmol), each mixed with sM2HA2 (15 μg) in the hind leg twice at 3-week intervals. Comparative serum IgG antibody titers at 168 days postimmunization. sM2HA2 specific IgG antibody titers at 1:100 serum dilution ratio after 168 days of first immunization (n = 7 mice per group). The data are presented as mean ± s.d. Analysis was performed by one-way ANOVA with Tukey's multiple comparison test. P values are indicated (n.s., not significant; * P < 0.05, ** P < 0.01, *** P < 0.001, **** P < 0.0001).

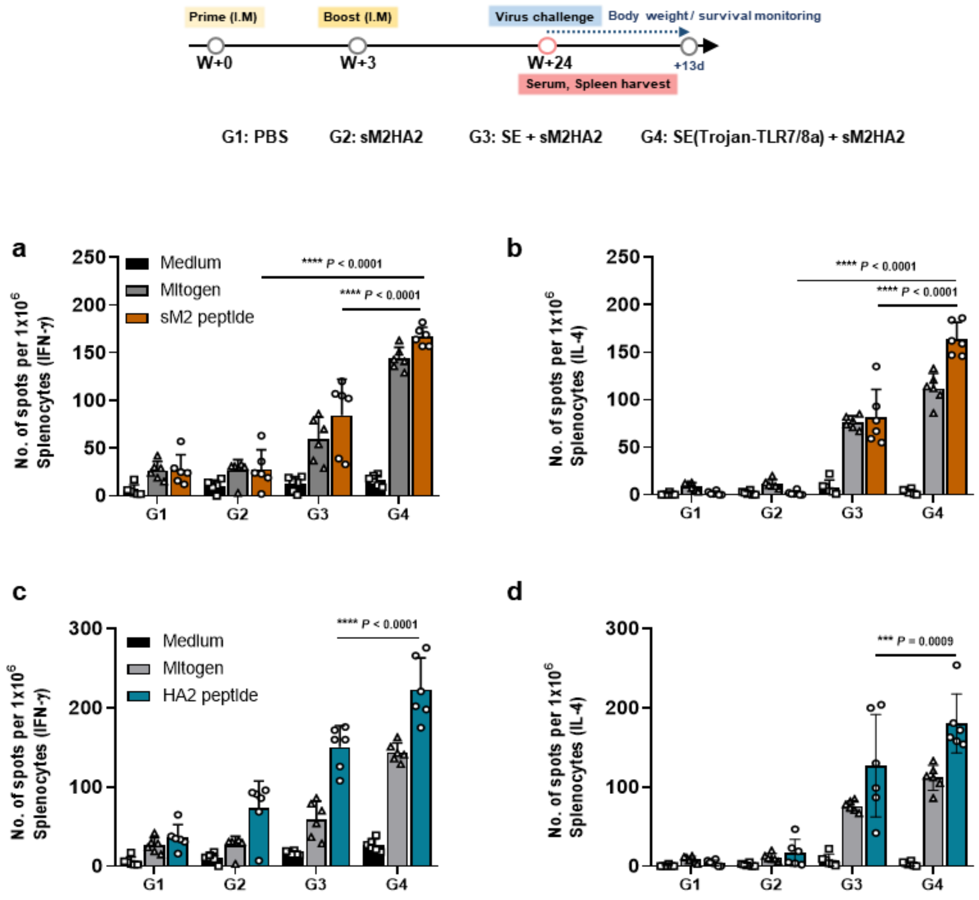

Supplementary Figure 34. Long-lasting T cell response SE(Trojan-TLR7/8a) adjuvanted sM2HA2. a-d, Mice were immunized intramuscularly with sM2HA2 (15 μg) or sM2HA2 in combination with SE, SE(Trojan-TLR7/8a) (72.1 μg, 79.5 nmol) in the hind leg twice at 3-week intervals. The number of sM2 or HA2 specific IFN-γ spot forming units (**a, c**) and IL-4 spot forming units (**b, d**) were measured by ELISPOT assay after collecting splenocytes at 168 days post-immunization (*n* = 6 mice per group). The data are presented as mean ± s.d. In **a-d,** analysis was performed by two-way ANOVA with Tukey's multiple comparison test. *P* values are indicated (n.s., not significant; * $P < 0.05$, ** $P < 0.01$, *** $P < 0.001$, **** $P < 0.0001$).

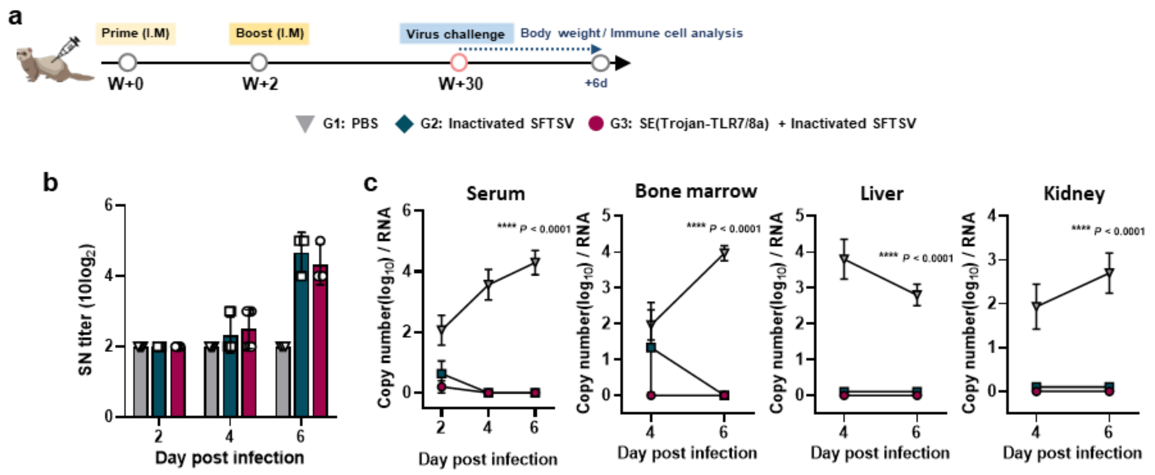

Supplementary 35. Evaluation of the efficacy of the SE(Trojan-TLR7/8a) vaccine against SFTSV-infected ferrets. a, Aged ferrets were intramuscularly immunized with inactivated SFTSV alone or in combination with SE(Trojan-TLR7/8a) (79.5 nmol) twice in weeks 0 and 2 and challenged with SFTSV 30 weeks after the initial immunization according to the schedule. **b,c,** On days 2, 4, and 6 after SFTSV infection, serum (**b,c**) and organ tissues (bone marrow, liver, and kidney) (**c**) from three ferrets per group were collected. Serum neutralizing titers were analysed (**b**) and the viral load was quantified by qRT–PCR (**c**). The data are presented as the mean ± s.d. In **b,** analysis was performed by two-way ANOVA with Tukey's multiple comparison test. *P* values are indicated (n.s., not significant; * $P < 0.05$, ** $P < 0.01$, *** $P < 0.001$, **** $P < 0.0001$).

Formulation	Mean diameter ± SD (nm)	PDI	Zeta potential (mV)	Encapsulation Efficiency (%)
**SE**	143.4 ± 40.5	0.239	-4.88 ± 1.81	
**SE-R848**	117.1 ± 32.2	0.173		N/A
**SE (Trojan-TLR7/8a)**	153.2 ± 43.4	0.178	-3.97 ± 2.97	98.9

Supplementary Table 1. Squalene-based nanoemulsion formulations. The size, PDI, zeta potential, and adjuvant contents of formulations. The size and zeta potential of the formulation was measured using dynamic light scattering (DLS). The concentration of TLR7/8a both in SE-R848 and SE(Trojan-TLR7/8a) was 1mg/ml. The encapsulation efficiency of SE-R848 was not available (N/A) due to fast release of encapsulated R848 from SE droplets during measurement. The data are presented as mean ± s.d.

Formulation	Lyophilization	Mean diameter ± SD (nm)	PDI
**SE (Trojan-TLR7/8a)**	Before	108.8 ± 30.9	0.241
	After	108.7 ± 31.9	0.292

Supplementary Table 2. Characterization of SE(Trojan-TLR7/8a) before and after lyophilization. The lyophilized SE(Trojan-TLR7/8a) was reconstituted with distilled water. The size of the formulation was measured using dynamic light scattering (DLS). The data are presented as mean ± s.d.

Group (mg/head)	Sex	Day1	Day2	Day7	Day15[§]	Gain	n
G1 (0.00)	Male	22.32±1.13	22.32±1.09	22.34±0.96	24.45±1.37	2.14±0.48	5
G2 (0.05)		22.56±0.59	21.92±0.44	23.44±0.68	24.87±0.92	2.31±0.77	5
G3 (0.10)		22.85±1.04	21.56±1.09	23.52±1.24	24.59±1.16	2.64±0.45	5
G4 (0.20)		22.72±0.44	20.88±0.32	23.57±0.68	25.61±1.37	2.89±1.29	5
G1 (0.00)	Female	19.33±0.82	19.23±0.57	19.76±0.62	20.91±0.97	1.58±0.32	5
G2 (0.05)		19.64±0.7	17.85±0.66**	19.46±0.43	20.60±0.78	0.97±0.64	5
G3 (0.10)		19.52±0.14	17.69±0.20**	19.74±0.34	21.22±0.76	1.70±0.70	5
G4 (0.20)		19.74±0.63	17.86±0.50**	20.06±0.56	21.97±0.37	2.23±0.85	5

Supplementary Table 3. Body weight gain of the experimental animals. Groups G1 to G4 received intramuscular injections of PBS and SE(Trojan-TLR7/8a) at concentrations of 159, 318, and 636 nmol, respectively (*n* = 5 mice per group). Body weight changes were monitored from day 1 to 15, with a slight decrease observed on day 2 in the female groups, which subsequently returned to baseline level. Body weights were measured before fasting (§).

Test	Unit	G1 (0.00)	G2 (0.05)	G3 (0.10)	G4 (0.20)	G1 (0.00)	G2 (0.05)	G3 (0.10)	G4 (0.20)
WBC	x10^3/μL	7.04±1.97	6.01±0.73	5.79±1.13	3.99±0.53	5.67±1.55	4.65±0.69	4.45±0.56	3.98±0.72
RBC	x10^6/μL	9.50±0.28	9.72±0.16	9.79±0.33	9.17±0.70	9.64±0.35	9.20±0.10*	8.98±0.20**	8.79±0.29**
HGB	g/dL	14.38±0.52	14.60±0.17	14.94±0.59	13.88±0.83	14.46±0.25	13.72±0.08**	13.56±0.21**	13.34±0.44*
HCT	%	51.76±2.14	51.84±0.47	52.52±2.36	49.88±2.39	51.32±1.62	48.72±0.74**	47.80±0.90**	46.86±1.58**
MCV	fL	54.52±0.72	53.36±0.60	53.68±1.00	54.50±2.18	53.28±0.73	52.98±0.56	53.22±0.38	53.34±1.13
MCH	Pg	15.16±0.13	15.04±0.18	15.26±0.23	15.14±0.28	15.02±0.33	14.94±0.11	15.12±0.28	15.14±0.34
MCHC	g/dL	27.78±0.38	28.16±0.26	28.44±0.48	27.80±0.60	28.22±0.55	28.16±0.40	28.38±0.37	28.42±0.30
RDW	%	11.30±0.29	11.68±0.11	12.24±0.31**	13.34±1.71	11.70±0.23	12.42±0.13**	12.32±0.51*	12.92±0.44**
HDW	g/dL	1.47±0.06	1.65±0.05**	1.63±0.03**	1.73±0.18**	1.68±0.04	1.79±0.05**	1.73±0.05	1.77±0.07*
PLT§	X10^3/μL	1162.80±30.95	1199.00±52.99	1181.75±127.01	1266.40±82.24	1003.40±56.84	1064.80±56.49	1074.40±63.23	890.20±188.00
MPV§	fL	8.04±0.15	8.14±0.11	7.80±0.41	8.10±0.07	8.12±0.26	7.86±0.15	8.02±0.31	7.92±0.39
PDW§	%	44.68±1.22	44.98±1.60	44.00±1.87	44.52±0.95	43.86±1.11	41.72±1.26	42.16±0.62	42.48±3.13
PCT§	%	0.93±0.04	0.98±0.04	0.93±0.14	1.03±0.07	0.81±0.03	0.84±0.04	0.86±0.04	0.71±0.14
Neu	%	5.22±1.58	4.86±0.78	5.26±1.44	7.16±1.40	4.86±1.22	6.84±0.45	6.20±1.28	6.42±1.73
Lym	%	90.80±2.19	90.14±1.23	89.56±2.03	85.72±1.73	90.60±1.02	87.24±1.44**	88.78±1.33	85.70±1.67**
Mono	%	1.46±0.32	1.76±0.46	1.74±0.27	2.10±0.50	1.68±0.31	2.06±0.66	1.34±0.21	1.68±0.33
Eos	%	1.32±0.70	1.84±0.55	2.32±0.75	4.00±2.30*	1.90±0.50	2.60±1.20	2.32±0.69	4.46±0.68**
Baso	%	0.28±0.13	0.38±0.18	0.20±0.07	0.22±0.13	0.26±0.09	0.28±0.08	0.26±0.09	0.28±0.11
Luc	%	0.90±0.28	1.02±0.28	0.90±0.27	0.84±0.23	0.70±0.25	0.96±0.09	1.10±0.41	1.46±0.59
Neu	x10^3/μL	0.35±0.05	0.29±0.02	0.31±0.11	0.29±0.07	0.27±0.11	0.32±0.06	0.28±0.07	0.26±0.08
Lym	x10^3/μL	6.42±1.90	5.42±0.73	5.19±0.99	3.42±0.49**	5.13±1.38	4.06±0.60	3.95±0.51	3.40±0.58
Mono	x10^3/μL	0.10±0.03	0.11±0.03	0.10±0.03	0.08±0.03	0.10±0.03	0.09±0.03	0.06±0.01	0.07±0.03
Eos	x10^3/μL	0.09±0.04	0.11±0.02	0.13±0.04	0.15±0.06	0.11±0.05	0.12±0.06	0.10±0.03	0.18±0.05
Baso	x10^3/μL	0.02±0.01	0.02±0.01	0.01±0.01	0.01±0.01	0.01±0.01	0.01±0.01	0.01±0.01	0.01±0.01
Luc	x10^3/μL	0.06±0.02	0.06±0.02	0.05±0.03	0.03±0.01	0.04±0.02	0.04±0.01	0.05±0.02	0.06±0.04
Reti	%	3.75±0.14	4.00±0.30	4.65±0.20	9.09±8.88	4.26±0.88	5.02±0.54	4.95±0.35	6.41±0.87**
Reti	x10^9/μL	356.74±21.96	388.38±27.91	454.54±20.24	789.48±683.84	408.70±73.85	461.66±47.34	443.66±24.47	560.80±60.13**
n		5	5	4 or 5	5	5	5	5	5
Sex		M				F			

Supplementary Table 4. Evaluation of drug toxicity in blood through haematological tests following intramuscular immunization. Groups G1 to G4 received intramuscular injections of PBS and SE(Trojan-TLR7/8a) at concentrations of 159, 318, and 636 nmol, respectively (*n* = 5 mice per group). Before blood collection, animals were fasted for more than 4 hours (water was provided). Blood samples were collected during necropsy and then transferred into tubes treated with EDTA. The samples were subsequently analysed using an automated haematology analyzer (ADVIA 2120i, SIEMENS, Germany) for the following parameters. Mean values were calculated except the sample with the microclots (§).

Test	Unit	G1 (0.00)	G2 (0.05)	G3 (0.10)	G4 (0.20)	G1 (0.00)	G2 (0.05)	G3 (0.10)	G4 (0.20)
AST	U/L	51.88±10.12	87.82±75.82	62.28±23.54	73.36±16.98	89.26±43.53	75.60±12.77	69.98±28.39	86.84±36.78
ALT	U/L	26.78±5.06	83.06±100.47*	33.34±2.47*	43.40±11.39*	35.12±12.58	26.30±5.41	31.32±10.55	28.38±6.02
ALP	U/L	404.24±27.26	401.78±20.39	421.80±12.72	424.86±31.47	524.90±43.10	561.46±36.47	555.32±39.31	514.14±40.08
TBIL	mg/dL	0.01±0.02	0.01±0.01	0.00±0.00	0.00±0.00	0.03±0.02	0.02±0.02	0.02±0.01	0.05±0.07
TPRO	g/dL	4.60±0.00	4.58±0.13	4.58±0.15	4.46±0.11	4.56±0.21	4.48±0.13	4.36±0.15	4.26±0.15
ALB	g/dL	1.64±0.05	1.58±0.04	1.60±0.07	1.50±0.07*	1.66±0.05	1.54±0.05*	1.54±0.05*	1.40±0.0[illegible]
GLOB	g/dL	2.96±0.05	3.00±0.10	2.98±0.08	2.96±0.05	2.90±0.16	2.94±0.09	2.82±0.11	2.86±0.09
BUN	mg/dL	18.32±1.63	18.34±2.88	17.78±1.25	16.28±2.37	16.88±0.98	14.10±1.73	18.16±1.92	16.32±3.66
CREA	mg/dL	0.32±0.02	0.32±0.03	0.34±0.03	0.32±0.03	0.33±0.03	0.35±0.03	0.31±0.03	0.31±0.02
TCHO	mg/dL	107.20±4.02	108.20±8.79	109.20±8.58	116.60±5.77	92.20±11.52	85.00±7.97	83.40±4.28	74.60±4.72
TG	mg/dL	44.60±12.86	52.40±9.99	59.60±17.30	58.00±15.28	51.40±14.05	39.00 ± 14.82	39.00±9.92	28.80±16.48
GLU	mg/dL	244.00±24.18	253.40±40.65	282.20±30.14	288.20±52.54	239.80±30.28	232.80±27.42	228.60±33.11	206.80±38.30
IP	mg/dL	7.65±1.24	7.44±1.32	8.07±0.71	8.93±0.89	8.73±1.96	8.33±1.83	8.38±1.36	8.90±1.51
Ca^{2+}	mg/dL	9.36±0.26	9.08±0.34	9.16±0.11	9.20±0.21	9.08±0.22	8.52±0.97	9.24±0.21	9.12 ±0.18
Na^{+}	mmol/L	148.52zt0.83	147.08±0.97	146.58±0.83	146.42±1.64	147.54±1.38	148.18±1.45	148.04±2.01	146.82±0.56
K^{+}	mmol/L	4.56±0.31	5.05±0.51	5.17±0.32	5.23±0.27	4.22±0.32	4.36±0.76	4.36±0.47	4.68±0.13
cr	mmol/L	109.56±0.17	108.94±1.16	107.76±1.16	108.24±1.42	110.86±2.09	111.82±1.13	112.30zL0.56	112.44±0.91
CK	U/L	2407.80±1391.96	1428.60±1011.30	2613.00±3264.19	3323.40±l 178.83	3571.20±3212.19	3557.00±l 132.62	2793.80±2082.34	3440.40±2477.72
n		5	5	5	5	5	5	5	5
Sex		M				F			

Supplementary Table 5. Evaluation of drug toxicity in blood through clinical biochemistry test following intramuscular immunization. Groups G1 to G4 received intramuscular injections of PBS and SE(Trojan-TLR7/8a) at concentrations of 159, 318, and 636 nmol, respectively (*n* = 5 mice per group). Blood samples collected at necropsy were transferred into serum separation tubes (SST) and then left to clot at room temperature for 15-20 min. The samples were centrifuged at 488g for 10 min at 4 °C to separate the serum, which was then analysed for the following indicators.

Protein	aa position	aa Sequence
HA2	19-48	GYAADLKSTQNAIDEITNKVNSVIEKMNTQ
M2	2-16	SLLTEVETPTRNEWE

Supplementary Table 6. Peptide used for ELISPOT. [a]*Proteins of the A/EM/Korea/W149/06 (H5N1) virus. aa, Amino acid.*

Antibody	Manufacturer	Clone	Catalog number
LN immunohistochemistry			
Alexa Fluor® 594 anti-mouse CD21/CD35 (CR2/CR1)	BioLegend	7E9	123426
Alexa Fluor® 647 anti-mouse/human GL7	BioLegend	GL7	144606
Flow Cytometry			
PerCP/Cyanine5.5 anti-mouse/human CD11b	BioLegend	M1/70	101228
Brilliant Violet 421™ anti-mouse CD11c	BioLegend	N418	117329
Alexa Fluor 647 anti-mouse Ly6G	BioLegend	1A8	127610
PE Rat anti-mouse Ly6C	BD Biosciences	AL-21	560592
PE anti-mouse CD8a	BioLegend	53-6.7	100708
APC anti-mouse CD103	BioLegend	2E7	121414
Brilliant Violet 510™ anti-mouse CD3	BioLegend	17A2	100233
FITC anti-mouse CD80	BioLegend	16-10A1	104705
PE anti-mouse/human CD45R/B220	BioLegend	RA3-6B2	103208
FITC anti-mouse CD95 (Fas)	BioLegend	SA367H8	152606
Brilliant Violet 421™ anti-mouse CD38	BioLegend	90	102732
Brilliant Violet 421™ anti-mouse CD185 (CXCR5)	BioLegend	L138D7	145511
PE anti-mouse CD279 (PD-1)	BioLegend	29F.1A12	135206
FITC Rat anti-mouse CD4	BD Biosciences	RM4-5	553047
Brilliant Violet 421™ anti-human/mouse Granzyme B	BioLegend	QA18A28	396414
APC anti-mouse TNF-α	BioLegend	MP6-XT22	506308
FITC anti-mouse IFN-γ	BioLegend	XMG1.2	505806
PE anti-mouse CD19	BioLegend	1D3/CD19	152408
APC anti-mouse/human CD45R/B220	BioLegend	RA3-6B2	103212
APC anti-mouse CD138 (Syndecan-1)	BioLegend	281-2	142501
Brilliant Violet 421™ ani-mouse/rat/human CD27	BioLegend	LG.3A10	124223
APC anti-mouse CD4	BioLegend	GK1.5	100412
PE anti-mouse IL-4	BioLegend	11B11	504104
PerCP anti-mouse CD3ε	BioLegend	145-2C11	100326
Brilliant Violet 510™ anti-mouse/human CD44	BioLegend	IM7	103043
Brilliant Violet 421™ anti-mouse CD62L	BioLegend	MEL-14	104435
APC anti-mouse CD40	BioLegend	3/23	124612
APC anti-mouse/human CD11b	BioLegend	M1/70	101212
Brilliant Violet 510™ anti-mouse F4/80	BioLegend	BM8	123135
PE anti-mouse CD206	BioLegend	C068C2	141706

Antibody	Manufacturer	Clone	Catalog number
Flow Cytometry			
FITC Rat anti-mouse CD8a	BD Biosciences	53-6.7	553030
PerCP-Cy™ 5.5 Hamster anti-mouse CD3	BD Biosciences	145-2C11	551163
PE Rat anti-mouse IFN-γ	BD Biosciences	XMG1.2	554412
PE-Cy™ 7 Rat anti-mouse IL-2	BD Biosciences	JES6-5H4	560538
Brilliant Violet 711™ ani-mouse CD19	BioLegend	6D5	115555
Alexa Fluor™ 488 anti-mouse/human GL7	Invitrogen	GL7	53-5902-82
AID Monoclonal Antibody (mAID-2), Biotin	Invitrogen	mAID-2	13-5959-82
Streptavidin PE conjugate	Invitrogen		12-4317-87
APC-Cy™ Rat anti-mouse CD4	BD Biosciences	GK1.5	561830
BV421 Rat anti-mouse CD279 (PD-1)	BD Biosciences	RMP1-30	569780
PE/Cyanine5 anti-mouse IL-21R	BioLegend	4A9	131908

Supplementary Table 7. List of antibodies used fluorescence imaging, and flow cytometry analysis.
